# Supramolecular “sergeants”: *in situ* and multi-level induction of chirality in helical assemblies of triarylamine trisamide monomers†

**DOI:** 10.1039/d5sc02159f

**Published:** 2025-07-10

**Authors:** Antoine Perennes, Quentin Sallembien, Weiwei Fang, Stéphane Grass, Jérôme Lacour, Laurent Bouteiller, Matthieu Raynal

**Affiliations:** a Sorbonne Université, CNRS, Institut Parisien de Chimie Moléculaire, Equipe Chimie des Polymères 4 Place Jussieu 75005 Paris France matthieu.raynal@sorbonne-universite.fr; b Department of Organic Chemistry, University of Geneva Quai Ernest Ansermet 30 1211 Geneva 4 Switzerland

## Abstract

The induction and transmission of chirality across multiple length scales is fundamental to many (bio)chemical processes. For the majority of macromolecular and supramolecular structures adopting a helical configuration, this is harnessed by means of a monomer embedding a stereogenic element, also called a “sergeant” because of its ability to transfer its chirality preference to achiral monomers. Herein, we devise a triarylamine trisamide (TATA) monomer embedding a (thio)urea unit able to interact with a chiral phosphate anion through hydrogen bonding. Thanks to the orthogonal nature of the amide and (thio)urea functions, the anion specifically binds to the (thio)urea unit, thus yielding a supramolecular monomer acting as a “sergeant” *i.e.* allowing efficient chirality induction in amide-bonded TATA helical copolymers composed of various types of achiral TATA monomers. Unlike covalent “sergeants”, chirality can be induced *in situ* by binding of the chiral anion to pre-formed coassemblies. In addition, the catalytic performance of TATA coassemblies embedding intrinsically achiral phosphine-functionalized TATA monomers has been evaluated: higher enantioselectivities are reached with the supramolecular *versus* covalent “sergeant”. Our work may facilitate the design and development of supramolecular “sergeants” as a modular approach to induce chirality in supramolecular helical copolymers and catalysts.

## Abstract 2

Triarylamine trisamide copolymers and catalysts are formed by chirality induction between a supramolecular “sergeant”, combining a (thio)urea-functionalized monomer (in grey) with a chiral anion (in blue), and various types of “soldiers” (in red).

## Graphical abstract

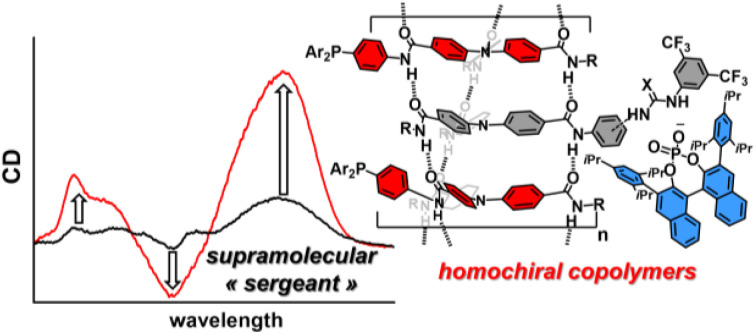

## Introduction

**Scheme 1:**
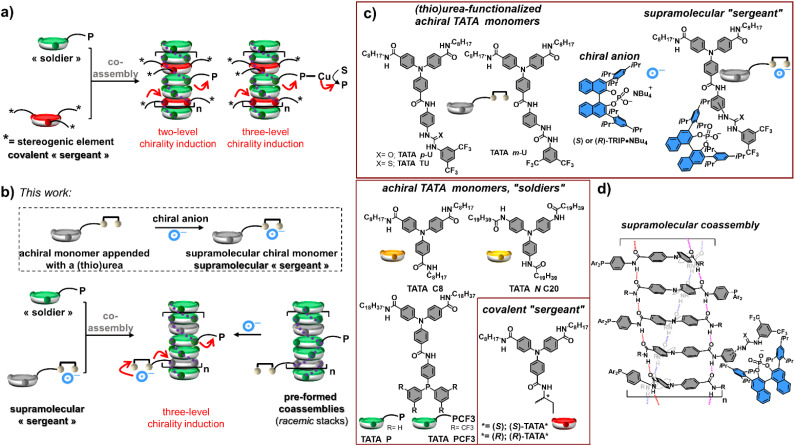
(a) Induction of chirality in supramolecular helices of disc-like monomers by means of conventionally used covalent “sergeants”. Red arrows: multi-level induction of chirality; from the stereogenic unit of the “sergeant” to the main chain of the polymer (1st level) to the phosphine ligand (2nd level) to the product of the catalytic reaction (3rd level). S = substrate, P = product. (b) Concept of this work: induction of chirality in supramolecular helices by means of a supramolecular “sergeant”. Specific binding of the chiral anion to the (thio)urea side chain constitutes an additional level of chirality induction. (c) Chemical structures of the different TATA monomers and of the chiral phosphate anions evaluated in this study. (d) Schematic representation of the hydrogen-bonded TATA helices with specific binding of the chiral anion to the (thio)urea side chain. For the sake of simplicity, only the assembly of *C*-centred TATA monomers, with amide functions connected to TA through their carbon atoms, is represented.

The transmission of chiral information at various length scales is fundamental for the operation of biological systems and is pivotal for the development of the chiroptical and catalytic properties of synthetic materials.^1–5^ Chemical systems adopting a dynamic helical configuration are unique in that context because of the possibility to directly probe the efficiency of the chirality induction process. This is particularly true for dynamic helical systems such as foldamers and polymers for which a chiral bias can be applied to control the handedness and screw-sense excess of the main chain.^6–8^ Mixing chiral and achiral monomers is a convenient approach provided that the former (also called the “sergeants”) are able to transfer their chiral preference to the latter (the “soldiers”) through the well-established “sergeants-and-soldiers” effect.^9^ Previous examples also demonstrated that the chiral information can be transmitted over a large distance (up to 4 nm) through a “domino effect” *i.e.* by positioning a stereogenic centre at the terminal position of a foldamer.^10^ Alternatively, very strong “sergeants-and-soldiers” (S&S) effect has been demonstrated for supramolecular polymers: single-handed polymers being obtained that contain as low as a few tenths of a percent of “sergeants”.^11–13^ Chirality information can be transmitted to an additional scale when intrinsically achiral catalytic groups are anchored to the macro- or supramolecular helices; enantioselectivity in the catalytic reaction is observed only when the chirality located at a remote position is efficiently transferred to the catalytic centre through multi-level chirality induction (Scheme 1a).^13–19^

Conventional “sergeants”, *i.e.* covalent “sergeants”, embed a stereogenic element next to the reactive or interacting groups, for covalent and supramolecular polymers, respectively.^6^ Given that the nature of the stereogenic group may strongly influence the structure and chiral properties of the polymers, a modular approach for the induction of chirality in these systems is highly desirable. This is quite well-established for covalent helical polymers for which non-covalent or reversible covalent interactions are harnessed between the polymer side chain and chiral “guests” in order to form single handed structures.^20^ Relevant applications include the possibility to memorize the helical chirality of the polymer backbone^21–25^ and the development of modular chiroptical sensors^26^ and asymmetric catalysts.^27–30^ The strategy is far less developed for supramolecular polymers. This is likely due to the fact that the binding of the chiral inducer must not compete with the formation of assemblies. In other words, orthogonality^31^ is required between the different types of interactions involved in the assembly process. Chiral solvents are potent chiral inducers for a certain number of supramolecular helical systems.^32–34^ A few strategies have been devised which involved organic molecules used as chiral additives, yet with different roles. The additive can both bring the chiral information and promote stacking, thus acting as a co-monomer or a template.^35–40^ Chiral amines,^41–43^ cations,^44,45^ or “seeds”^46,47^ were added to select the handedness of specific helical systems, formed through a mirror-symmetry breaking phenomenon, *i.e.* supramolecular assemblies and gels that are known to be sensitive to tiny chiral imbalances. Finally, only two studies dealt with supramolecular helical polymers embedding specifically designed receptors or binding sites. Schenning, Meijer and co-workers induced a preferential handedness by combining a chiral acid with π-stacked dimers of a ureidotriazine derivative; limited dynamicity did not allow chirality induction to occur at room temperature.^48^ Fenniri^49^ elegantly demonstrated chirality induction in helical rosette nanotubes, thanks to a specific binding of chiral amino acid to crown ether moieties located at the periphery of the nanotubes; chirality induction has an “all-or-none” nature since homochiral nanotubes were obtained provided that most of the binding sites were occupied by the chiral promoter.^50^ These examples were restricted to homopolymers, *i.e.* assembly of monomers embedding the receptor site.

Triarylamine trisamide (TATA) monomers belong to the family of disk-like monomers that stack upon each other in apolar solvents, thanks to hydrogen bonding interactions between their amide functions and aromatic interactions between the TA units.^51^ Chirality induction was previously achieved, thanks to the integration of covalent “sergeants” in the resulting supramolecular TATA copolymers.^11^ Herein, we develop a new strategy to induce chirality into TATA coassemblies by devising a supramolecular “sergeant” composed of a chiral phosphate anion bonded to the (thio)urea unit of an achiral TATA monomer (Scheme 1b and c). Despite the fact that both binding of the anion and stacking of the monomer rely on hydrogen bonding interactions, orthogonality between the (thio)urea and amide functions allows efficient control of the helical chirality in supramolecular coassemblies between this supramolecular “sergeant” and various achiral TATA monomers (“soldiers”). The design of the supramolecular “sergeant”, through specific interaction of the chiral inducer with the side chain of the TATA monomer, enables *in situ* control of the copolymer helicity at room temperature; homochirality being obtained with *ca.* 35% of “sergeants” in the copolymers. In addition, helical coassemblies embedding an achiral phosphine-functionalized TATA monomer proved to be enantioselective, thanks to the presence of the chiral anion at the remote position of the catalytic centre.

## Results and discussion

### Design and synthesis of the TATA monomers and the chiral anion

Thiourea- and urea-functionalized TATA monomers have been designed as follows (Scheme 1c): (i) *C*_2_-symmetry with two linear alkyl chains to facilitate solubilization in apolar solvents required for the assembly through hydrogen bonding and aromatic interactions, (ii) a rigid 1,3-phenylene (for TATA *m*-U) or 1,4-phenylene (for TATA *p*-U and TATA TU) linker between the (thio)urea function and the TATA core to ensure efficient transfer of stereochemical information from the chiral anion to the TATA core, (iii) CF_3_ groups at the 3 and 5 positions of the terminal aryl to strengthen the binding of the chiral anion.^52^ The followed synthetic route of these monomers includes the desymmetrization of the TATA core through partial hydrolysis of the triarylamine trimethyl ester starting material followed by successive hydrolysis and amide coupling reactions. TATA *m*-U, TATA *p*-U and TATA TU have been isolated in 20% (5 steps), 19% (5 steps) and 9% (6 steps) overall yield, respectively; synthetic protocols had not been specifically optimized. TATA ligands (TATA P and TATA PCF_3_) and covalent “sergeants” ((*S*) and (*R*)-TATA*) have been prepared similarly, also benefiting from our experience in the synthesis of benzene-1,3,5-tricarboxamide (BTA) ligands and “sergeants”.^53,54^*C*3-symmetrical non-functionalized TATA monomers (TATA C8 and TATA *N* C20) have been accessed in one or two steps from readily available precursors. All these monomers have been characterized by conventional techniques (see the ESI†).

The strategy for chirality induction presented in Scheme 1b is highly modular as in principle any chiral anions with an affinity for (thio)urea function could have been selected. For the purpose of demonstrating the feasibility of the concept, we have selected 3,3′-bis(2,4,6-triisopropyl-phenyl)-1,1′-binaphthyl-2,2′-diyl hydrogen phosphate (abbreviated as TRIP) given its ability to bind strongly to dual hydrogen-bond donors such as (thio)ureas.^55,56^ Both enantiomers of the tetrabutylammonium salt of TRIP have been prepared in one pot from the commercially available phosphoric acids (see the ESI†).^57^ These chiral salts proved to be well soluble in toluene and MCH, suitable solvents for enhancing both anion binding to the (thio)urea and polymerization of the TATA monomers through hydrogen bond and aromatic interactions.

### Characterization of the supramolecular “sergeant”

**Fig. 1:**
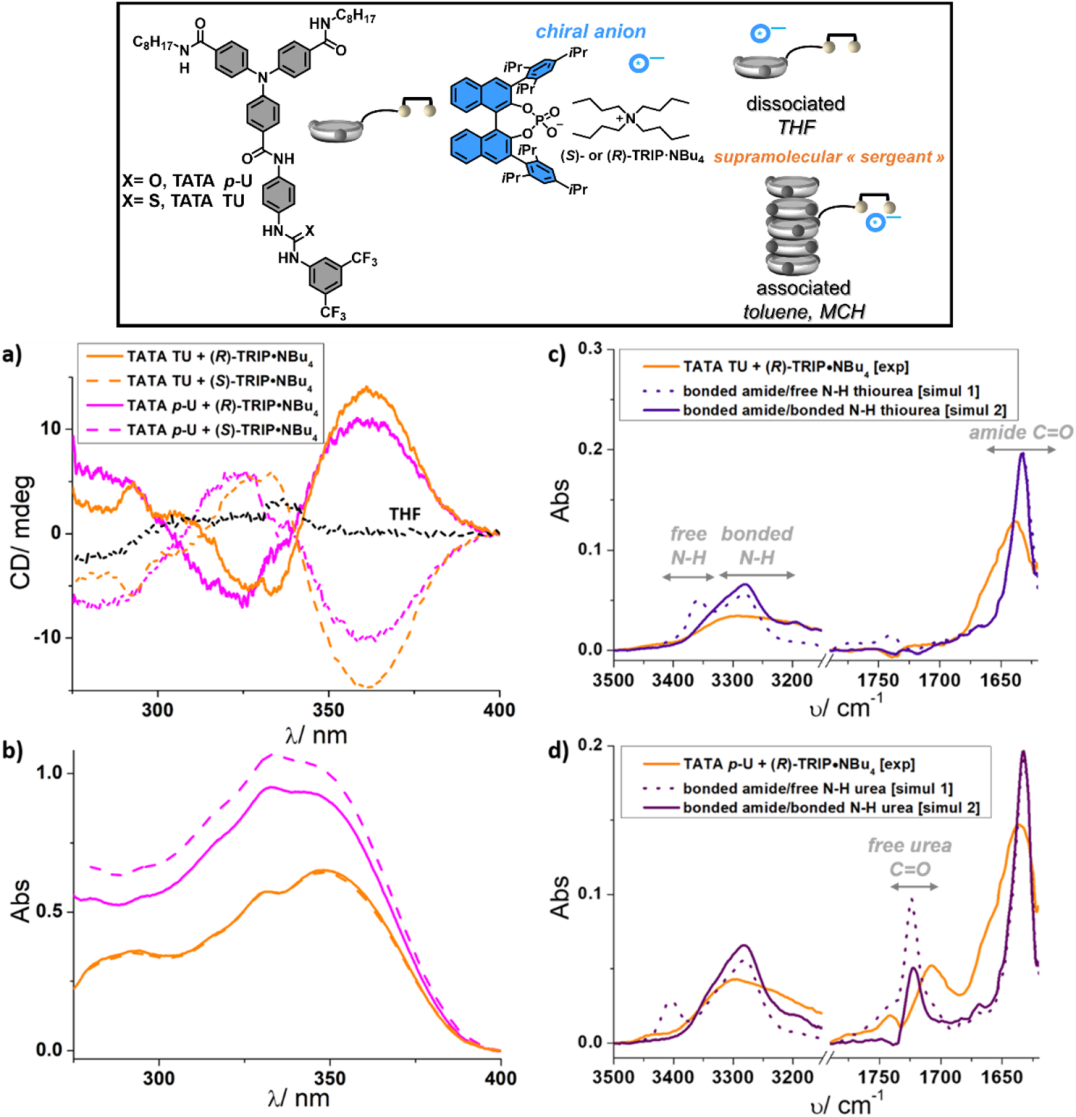
Characterization of the supramolecular “sergeant”. CD (a) and UV-Vis analyses (b) of the supramolecular “sergeants” formed by mixing TATA TU or TATA *p*-U (2.9 mM) with (*R*) or (*S*)-TRIP·NBu_4_ (2.9 mM) in toluene (1 : 1 mixture). The spectrum in THF is also shown for the mixture between TATA TU and (*S*)-TRIP·NBu_4_. The contribution of the signal coming from the chiral salt is subtracted (see Fig. S2 and S4† for non-subtracted spectra). (c) FT-IR analyses of the “supramolecular sergeant” formed by mixing TATA TU with (*R*)-TRIP·NBu_4_ in toluene (1 : 1 mixture, 5.8 mM total concentration). (d) FT-IR analysis of the “supramolecular sergeant” formed by mixing TATA *p*-U with (*R*)-TRIP·NBu_4_ in toluene (1 : 1 mixture, 5.8 mM total concentration). Zoom on the N–H and C=O regions. FT-IR spectra for supramolecular stacks with free (thio)urea functions [simul. 1] and (thio)urea functions bonded to (*R*)-TRIP·NBu_4_ [simul. 2] have been simulated as indicated in the ESI.†

(Thio)urea-functionalized TATA monomers, TATA *m*-U, TATA *p*-U and TATA TU, are not soluble on their own in toluene and MCH. Fourier-transform infrared (FT-IR) analyses indicate that both amide and (thio)urea functions participate in the hydrogen-bonding network present in the solid state (Fig. S1†); the strong interactions between monomers probably preclude their dissociation by these solvents. However, TATA TU readily solubilizes in toluene in the presence of, at least, 0.3 equivalent of TRIP·NBu_4_. Circular Dichroism (CD) analyses of the mixtures with different amounts of (*S*)-TRIP·NBu_4_ reveal (Fig. S2a†): (i) the presence of CD bands that do not belong to TRIP·NBu_4_, and (ii) an increase in the CD intensity of these bands with the proportion of TRIP·NBu_4_ relative to TATA TU. Subtracting the intrinsic contribution of (*S*)-TRIP·NBu_4_ to the CD spectra of the mixtures (Fig. 1a dotted orange curve, Fig. S2c†) shows an alternation of positive/negative/positive signals in the 275–400 nm region. Similar signals are detected in MCH but additional bands can be observed in this solvent (Fig. S3a and c†): notably a bisignate CD signal in the 250 nm region with an intense negative band at 238 nm. All these induced CD bands are absent in THF (Fig. 1a and S3a†); solvation of both the TATA monomer and the chiral salt precludes their interaction in this medium. Similar observations are made with TATA *m*-U and TATA *p*-U, except that in those cases one equivalent of TRIP·NBu_4_ is required for solubilization (this point will be further commented below). CD and UV-Vis spectra of equimolar mixtures between these monomers and the enantiomers of TRIP·NBu_4_ are shown in Fig. 1a, b and S4† (5.8 mM total concentration). Relative to the CD spectra obtained by titration with (*S*)-TRIP·NBu_4_ of a thiourea compound lacking the triarylamine trisamide core used as model, CD spectra of these TATA complexes exhibit well-defined CD signals in the 275–400 nm range (Fig. S5†). All these analyses indicate that the interaction between the TRIP anion and (thio)urea-functionalized TATA monomers leads to an efficient induction of chirality, which is not restricted to the (thio)urea function but is also communicated to the TATA core of the monomers.

We next conducted experiments to better apprehend the structure of the adduct formed between TATA TU or TATA *p*-U and TRIP·NBu_4_. First, a TATA monomer, named the TATA model, was studied which is similar to TATA TU or TATA *p*-U but lacks the terminal (thio)urea function (Fig. S6†). This monomer fully assembles into (racemic) helical stacks in toluene, as deduced from its FT-IR spectrum showing characteristic bands for associated amide functions, as well as its ability to form a gel, as a probable result of the formation of entangled fibres. Upon addition of one equivalent of (*R*)-TRIP·NBu_4_, the gel is weaker but the viscosity of the solution remains high (Fig. S6a†). In addition, no significant change and induced CD band can be detected from FT-IR and CD analyses, respectively (Fig. S6b and c†). This indicates that the interaction between the chiral anion and the amide functions of TATA assemblies is not very strong; thus TRIP·NBu_4_ is not a good chain stopper of the supramolecular chains formed by TATA monomers.^58^ Second, the equimolar mixtures between TATA TU or TATA *p*-U and (*R*)-TRIP·NBu_4_, identical to those characterized by CD, have also been analysed by FT-IR (Fig. 1c and d). The obtained experimental spectra were compared with simulated ones for supramolecular amide-bonded stacks of TATA TU or TATA *p*-U with (thio)urea functions free or bonded to (*R*)-TRIP·NBu_4_, corresponding to simulations 1 and 2, respectively (see Fig. S7 and the ESI† for the procedures). Experimental spectra of the mixtures are closer to simulation 2 since no (or few) free N–H group can be detected (above 3350 cm^−1^). For TATA *p*-U, free C=O (1707 cm^−1^) belonging to the urea functions can be observed which is consistent with urea acting as a dual hydrogen-bond donor of the TRIP anion. The band attributed to amide C=O (maximum at 1640 cm^−1^) is rather broad for both mixtures, this can be due to the fact that the supramolecular stacks are either short or contain some structural defects, probably because of the steric hindrance generated by the TRIP anion located in the side chain. These experiments support the orthogonality of the interaction of the anion which is selective for the thiourea and urea functions, consistent with previous reports in the literature for which the association constant of the dihydrogen phosphate anion for (thio)urea was two orders of magnitude greater than that for amide.^59^ Based on these analyses, the assembled two-component aggregate composed of the TATA monomer and the chiral anion embeds the TRIP anion that is non-covalently bonded to the lateral (thio)urea group; it can thus be considered as a supramolecular “sergeant”.

The stability of the supramolecular “sergeants” was subsequently probed by CD analyses conducted between 293 K and 393 K (Fig. S8†). These analyses are consistent with the dissociation of the (thio)urea – chiral anion supramolecular couple upon increasing the temperature. This disassembly process is gradual for both TATA *p*-U and TATA TU. Even though the different assembly behaviour between these two monomers is difficult to assess quantitatively, we noticed that: (i) a higher fraction of the chiral anion is necessary to dissolve TATA *p*-U, (ii) the intensity of the induced CD signal above 250 nm for TATA *p*-U is lower than that observed for TATA TU (Fig. 1a, S4 and S8†) and, (iii) the solution containing the supramolecular “sergeant” composed of TATA *p*-U tends to precipitate over time (Fig. S8†). All these observations suggest that the interaction between TATA TU and TRIP·NBu_4_ is stronger than that of TATA *p*-U with the same anion, as a probable result of the higher acidity of the thiourea function.^52^ The next experiments will be conducted at 293 K, a temperature for which the chiral anion is strongly bound to the (thio)urea function.

### Transfer of chirality to different “soldiers”

**Fig. 2:**
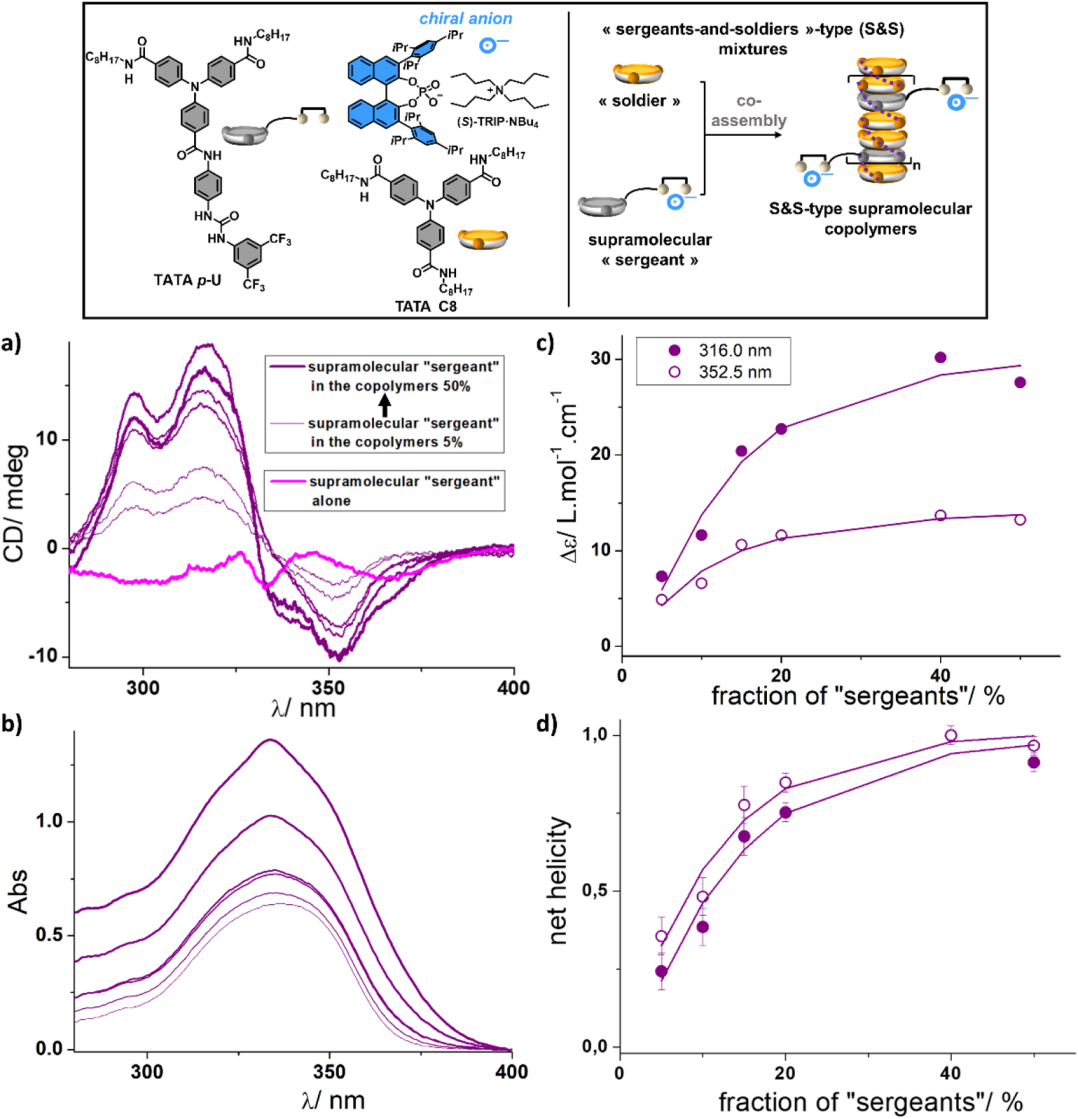
Transfer of chirality of the supramolecular “sergeant” to the “soldier”, TATA C8. (Top) Schematic representation of the copolymerization between the supramolecular “sergeant” and TATA C8. (a) CD analyses of the mixtures containing the supramolecular “sergeant” (1 : 1 mixture of TATA *p*-U and (*S*)-TRIP·NBu_4_) and TATA C8 (0.20 mM) in toluene. The amount of supramolecular “sergeant” in the mixtures is equal to 0.01 mM, 0.02 mM, 0.04 mM, 0.05 mM, 0.13 mM and 0.20 mM corresponding to fractions of supramolecular “sergeant” of 5%, 10%, 15%, 20%, 40%, and 50%, respectively. The fraction of “sergeant” is defined as the ratio of the concentration in TATA *p*-U to the total concentration in TATA monomers (TATA *p*-U + TATA C8) in the mixtures. The spectrum of the supramolecular “sergeant” alone composed of TATA *p*-U and (*S*)-TRIP·NBu_4_ (0.2 mM + 0.2 mM) is shown for comparison. (b) UV-Vis spectra corresponding to data in (a). (c) Plot of the molar CD values (Δ*ε*) as a function of the fraction of supramolecular “sergeant” in the mixtures. The molar CD values are reported at two different wavelengths after subtracting the contribution of the supramolecular “sergeant” and by considering only the concentration of TATA C8 for the remaining CD signal (Fig. S9†). Δ*ε* = *θ*/(32 982 × [TATA C8] × *l*), with *θ* = ellipticity (in mdeg), [TATA C8] = concentration in TATA C8 (in mol L^−1^), and *l* = cell pathlength (in cm). (d) Plot of the net helicity as a function of the fraction of supramolecular “sergeant” in the mixtures. The net helicity corresponds to the ratio between Δ*ε* of the mixture and Δ*ε* max (maximal Δ*ε* values measured for the different mixtures). For (c) and (d), the lines are a guide to the eye.

In order to probe the possibility to induce chirality with the newly designed supramolecular “sergeants” in supramolecular copolymers, we first selected TATA C8 as the “soldier”, which is devoid of any chiral anion binding group but has the same TATA core necessary for coassembly through hydrogen bonding and aromatic interactions. The couple composed of TATA *p*-U and (*S*)-TRIP·NBu_4_ (1 : 1 ratio) was selected as the supramolecular “sergeant”. CD analyses of mixtures containing various fractions of the “sergeant” have been performed; the concentration in TATA C8 being set constant to 0.2 mM (Fig. 2a and S9†). Mixtures were prepared sequentially; the solution containing the supramolecular “sergeant” was prepared independently and added to the “soldier”. Comparison of the CD spectra of the mixtures with that obtained for the supramolecular “sergeant” alone shows major differences: CD patterns are different and more strikingly, the intensity of the CD bands is drastically higher for the mixtures (Fig. 2a). The CD signals in the 275–400 nm region for the mixtures with three maxima at *λ* ≈ 353 nm, *λ* ≈ 319 nm and *λ* ≈ 298 nm and a crossover point at *ca.* 332 nm are characteristic of helical stacks of TATA monomers with a preferred handedness.^11,60^ In addition, mixtures embedding 40% and 50% of the supramolecular “sergeant” exhibit a CD spectrum which is virtually identical to that of (*S*)-TATA*, a covalent “sergeant” (Fig. S9†). The molar CD values actually plateau for a fraction of “sergeants” in the mixtures being equal or greater than 40% (Fig. 2c). All these analyses are consistent with the ability of the supramolecular “sergeant”, composed of TATA *p*-U and (*S*)-TRIP·NBu_4_, to coassemble with TATA C8 and impose its preferred handedness, eventually leading to homochiral stacks when at least 40% of sergeants are present in the coassemblies (Fig. 2d, schematic representation of the coassemblies in the upper part of Fig. 2).

**Fig. 3:**
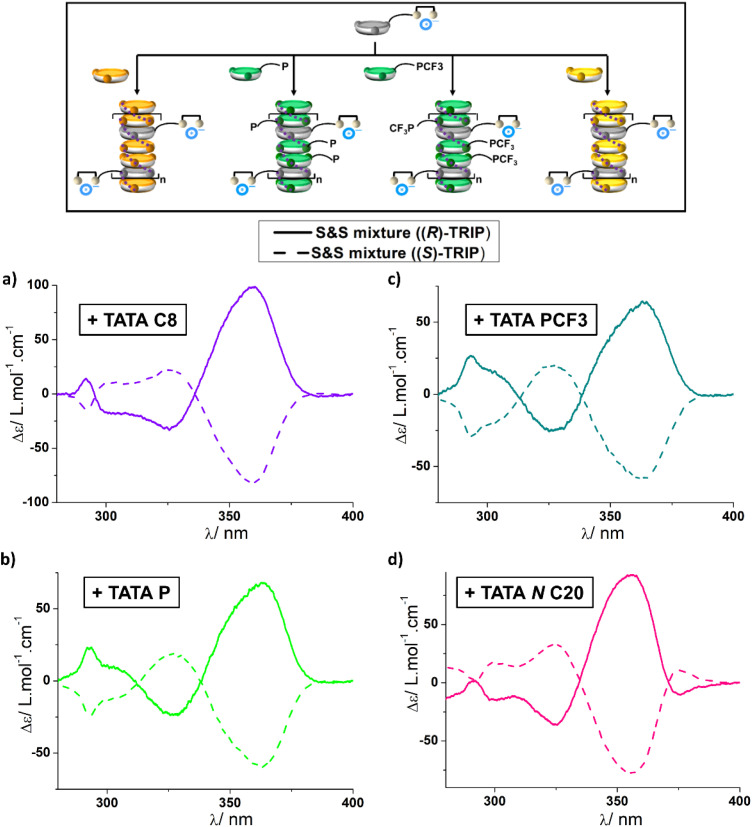
Transfer of chirality of the supramolecular “sergeant” to other “soldiers”. (Top) Schematic representation of the copolymerization between the supramolecular “sergeant” and various “soldiers”. The supramolecular “sergeant” consists of an equimolar mixture between TATA TU and either (*S*)-TRIP·NBu_4_ or (*R*)-TRIP·NBu_4_ (2.9 mM + 2.9 mM). CD analyses of the “sergeants-and-soldiers”-type mixtures (fs = 50%) containing TATA C8 (a), TATA P (b), TATA PCF3 (c) and TATA *N* C20 (d) as “soldiers” (2.9 mM). CD spectra have been processed as follows: the contribution of the supramolecular “sergeant” was subtracted from the pristine CD spectra and CD values were converted into molar CD values by considering only the concentration of the “soldier”. The difference in concentrations for the mixtures was also considered. See Fig. S10–S13† for the pristine CD and UV/Vis spectra.

We next tested the scope of this chirality induction process by examining other TATA coassemblies (Fig. 3). This time, the combination of TATA TU and TRIP·NBu_4_ was selected as the supramolecular “sergeant” and achiral TATA monomers of diverse chemical structures as “soldiers”, including one “soldier” (TATA *N* C20)^61,62^ with amide functions connected to their N atoms to the TA core, *i.e.* inverted connectivity relative to TATA TU. All mixtures contain a fraction of supramolecular “sergeant” of 50%. In all cases, the CD signals of the “sergeants-and-soldiers”-type mixtures are of higher intensity than those of the supramolecular “sergeant” alone (Fig. S10–S13†).

CD spectra are similar regardless of the “soldier” and are characterized by a dominant CD band at *λ* ≈ 360 nm and two minor bands of opposite intensities with one maximum at *λ* ≈ 325 nm and one at *λ* ≈ 295 nm. Two crossover points are thus detected, the first one at *λ* = 340 nm, which is common to all mixtures, while the position of the second one varies according to the nature of the “soldier”. The different CD signatures for the coassemblies depending on whether TATA *p*-U/TRIP·NBu_4_ or TATA TU/TRIP·NBu_4_ is used as “sergeant” suggest that these two supramolecular “sergeants” induce different conformations in the coassemblies. Anyway, the higher intensity of the CD signals (relative to the “sergeant”) and the fact that enantiomers of TRIP·NBu_4_ furnish mirror-image CD spectra are consistent with transfer of chirality of the supramolecular “sergeant” to helical TATA coassemblies for all “soldiers” as represented schematically in the upper part of Fig. 3. This includes chirality induction in TATA ligands, TATA P and TATA PCF3, a crucial point for their implementation in asymmetric catalysis (see below). In the case of TATA P, coassembly also occurs in MCH, and additional bands specific to the copolymers are detected in this solvent (Fig. S11†). Monomer TATA *N* C20, despite having a different connection of its amide functions relative to TATA TU, also coassembles with the supramolecular “sergeant”. The ability of monomers with different amide connectivities to coassemble into the same stacks was previously demonstrated for BTA-based supramolecular polymers.^63^ Our approach appears quite general and may be applied for the construction of various helically biased TATA copolymers.

### Chirality induction in pre-formed TATA copolymers

**Fig. 4:**
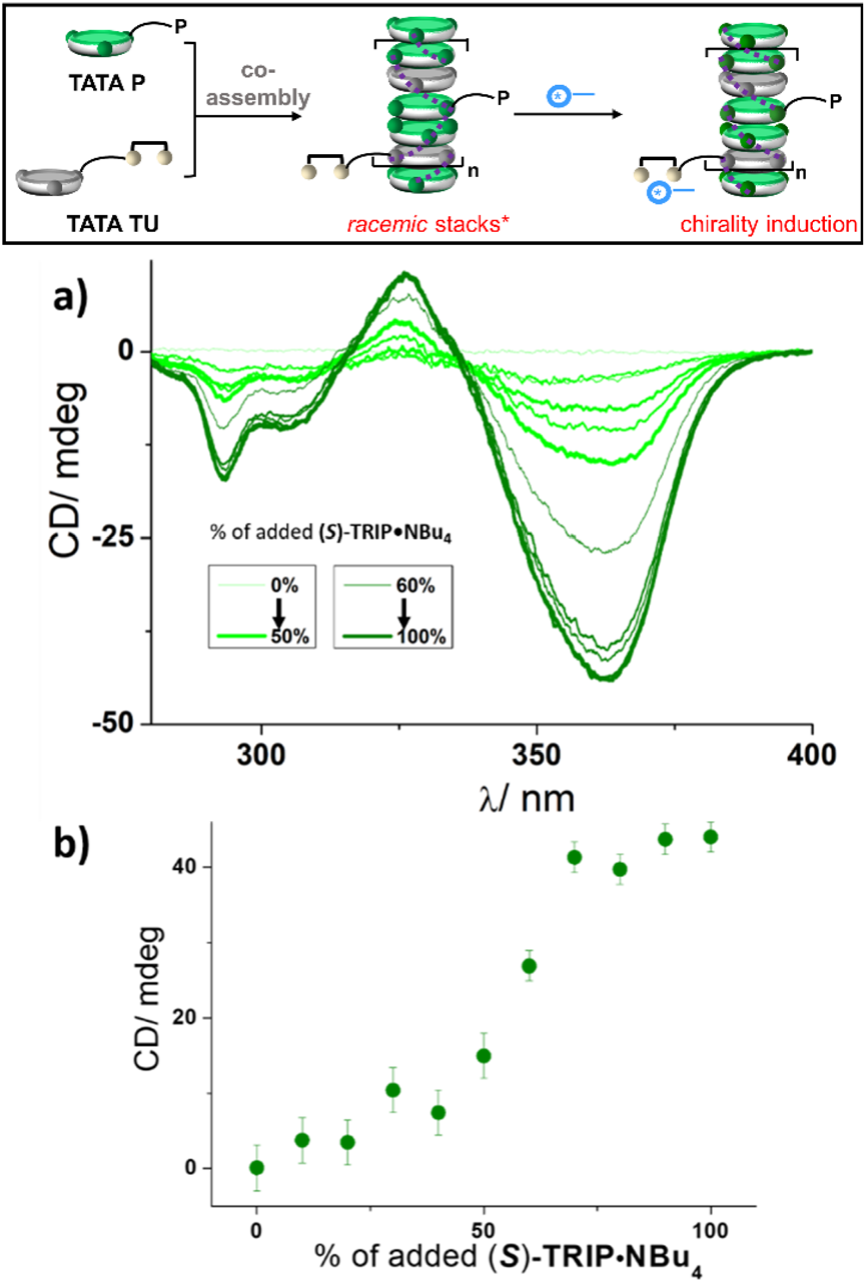
Chirality induction in pre-formed TATA copolymers. (Top) Schematic representation of the formation of helically biased TATA copolymers by addition of the chiral anion to pre-formed TATA coassemblies. *The thiourea functions in the racemic stacks are not free (see Fig. S15†). (a) CD spectra of “sergeants-and-soldiers”-type mixtures obtained by adding (*S*)-TRIP·NBu_4_ (0–100% relative to TATA TU, 0–2.9 mM) to pre-formed coassemblies between TATA P (2.9 mM) and TATA TU (2.9 mM) in toluene. (b) Plot of the CD signal (*λ* = 363.0 nm) as a function of the percentage of (*S*)-TRIP·NBu_4_ added to the mixtures (relative to TATA TU).

One advantage of the present concept is the possibility to induce chirality with anions in pre-formed TATA copolymers composed of achiral monomers that are thus initially composed of an equal amount of left-handed and right-handed helical fragments (see a schematic representation in the upper part of Fig. 4). We first probe the possibility to induce chirality in copolymers of TATA TU and TATA P by addition of (*S*)-TRIP·NBu_4_ at room temperature. The resulting CD spectrum after three minutes of mixing is almost identical to the one obtained after a heating/cooling cycle of the solution obtained by adding the supramolecular “sergeant” to TATA P (Fig. S14†). This indicates that the TATA coassemblies are sufficiently dynamic to bind the TRIP anion and reach the thermodynamic state without heating. This may constitute a hallmark of chirality induction through the supramolecular “sergeant” approach, since chirality induction can be made *in situ*, *i.e.* supramolecular chirality emerges in a system devoid of chiral monomers. Further studies are needed to determine the exact time required to induce chirality *in situ* which may be relevant for comparison with other systems in the literature.^13^

We examined in more detail the dynamic induction of chirality in pre-formed stacks of TATA P and TATA TU. Incremental quantities of (*S*)-TRIP·NBu_4_ were added to equimolar mixtures of TATA P and TATA TU. CD (Fig. 4a) and FT-IR (Fig. S15†) analyses of the corresponding solutions were performed. CD spectra (Fig. 4a) exhibit a nonlinear increase of the intensity of the CD bands relative to the percentage of (*S*)-TRIP·NBu_4_ added to the mixtures. This is particularly obvious for the CD band at *λ* = 363.0 nm (Fig. 4b). This trend can be explained by considering two extreme states for the TATA copolymers. In the first state, there is no (or very low) chirality transfer to TATA P, *i.e.* the amount of supramolecular “sergeant” formed is not sufficient to induce a preferred handedness in the copolymers. In the second state, the quantity of “sergeants” is sufficient to bias the helicity of the copolymers. The nonlinear increase of the CD signal is an attribute of the “sergeants-and-soldiers” effect, as further corroborated by the fact that a linear evolution of the CD signal is observed when a molecular thiourea model is mixed with (*S*)-TRIP·NBu_4_ (Fig. S5†). FT-IR analyses of the mixtures (Fig. S15†) are also consistent with two main states for the coassemblies since two types of FT-IR spectra with small but significant shifts of the N–H and C=O stretching frequencies are observed for mixtures with low and high amounts of (*S*)-TRIP·NBu_4_. FT-IR analyses bring the additional information that at least part of the thiourea functions are bonded to each other in the initial TATA copolymers (see the notes in the captions of Fig. 4 and S15†). It thus appears that binding of the TRIP anion with the thiourea competes with the ability of thiourea functions to bind together. If we consider that all anions are bonded to thiourea, full chirality induction is achieved for a fraction of supramolecular “sergeants” in the coassemblies of *ca.* 35% ([(*S*)-TRIP·NBu_4_]/(TATA TU + TATA P) for the plateau in Fig. 4b). This is consistent with the quantity of supramolecular “sergeants” required to fully bias the sense of rotation of the TATA copolymers embedding TATA C8 as the “soldier” (Fig. 2).

### Implementation in asymmetric catalysis

The aforementioned analytical data convincingly support the possibility to use the supramolecular “sergeant” as a chiral inducer in catalytic TATA copolymers, thanks to its ability to provide a preferred handedness to TATA helices embedding an achiral TATA ligand, such as TATA P and TATA PCF3 (Fig. 3 and 4). The role of this supramolecular “sergeant” is anticipated to be similar to that of conventional covalent “sergeants” that have been used by our group to develop BTA-based helical catalysts, with multi-level induction of chirality as represented in Scheme 1a.^13,15,17,18,54^

The copper-catalyzed hydrosilylation of 4-nitroacetophenone has been selected as a benchmark reaction^64^ to test whether chirality induction into the TATA copolymers can be transferred to the copper atoms, centers of the asymmetric catalytic reaction. Initial tests help to identify TATA PCF3 as a more efficient ligand than TATA P; the presence of the CF_3_ groups on the aryl rings connected to the phosphorus atom was also beneficial in the case of BTA-based helical catalysts (Table S1†).^54^ Supramolecular and covalent “sergeants” were thus evaluated with TATA PCF3 for the catalytic reaction. The fraction of “sergeant” in the mixtures of 50% ensures full induction of chirality, as probed above by CD measurements (Fig. 4). (*R*)-TRIP·NBu_4_, on its own, does not induce selectivity in the reaction (Table 1, entry 1). Limited conversion in this case can infer competitive binding of the TRIP anion to the copper atoms. The supramolecular “sergeant” composed of TATA TU and TRIP led to no conversion (entries 2 and3). This can be explained by the well-established strong interaction between copper(i) and sulfur, *i.e.* thiophilicity which prevents coordination of the substrate to the copper center.^65^ We were pleased to see that full conversion was achieved when urea-functionalized TATA monomers were present in the catalytic mixtures. More interestingly, TATA *p*-U displayed significant enantioselectivities since both enantiomers of 4-nitroacetophenol were obtained with *ca.* 30% enantiomeric excess (ee) depending on the configuration of the TRIP anion (entries 6 and 7). The same mixture was poorly active and selective in THF (entries 8 and 9), further corroborating that chirality induction occurs within TATA copolymers. In contrast, TATA *m*-U showed no selectivity: this suggests either non-optimal coassembly between TATA PCF3 and TATA *m*-U or an unfavorable geometry preventing transfer of chirality to the copper atom (entries 4 and 5).

**Table 1:** Supramolecular helical TATA catalysts embedding a supramolecular or covalent “sergeant”: evaluation in the copper-catalyzed hydrosilylation of 4-nitroacetophenone. Catalytic mixtures are composed of TATA PCF3 (coordinated to Cu) as the “soldier” and of the supramolecular or covalent “sergeants”. The fraction of “sergeant” in the catalytic mixtures is 50%. Conversion and enantiomeric excess (ee) were determined by chiral GC analysis. ee values are indicated as positive and negative when (*S*)-NPnol and (*R*)-NPnol are the major enantiomers, respectively. See the ESI for more details

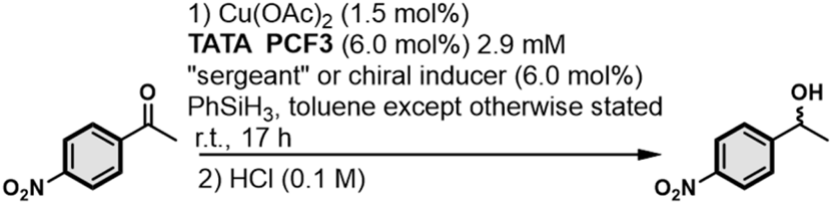			
Entry	“Sergeant” or “chiral inducer”	Conversion	ee ± 2 (%)
1	(*R*)-TRIP·NBu_4_	50%	+2
2	TATA TU + (*S*)-TRIP·NBu_4_	0%	n.d.
3	TATA TU + (*R*)-TRIP·NBu_4_	0%	n.d.
4	TATA *m*-U + (*S*)-TRIP·NBu_4_	84%	0
5	TATA *m*-U + (*R*)-TRIP·NBu_4_	99%	0
6	TATA *p*-U + (*S*)-TRIP·NBu_4_	99%	−35
7	TATA *p*-U + (*R*)-TRIP·NBu_4_	98%	+30
8^a^	TATA *p*-U + (*S*)-TRIP·NBu_4_	40%	+5
9^a^	TATA *p*-U + (*R*)-TRIP·NBu_4_	13%	−5
10	(*S*)-TATA*	92%	−8
11	(*R*)-TATA*	95%	+9

a In THF.

**Fig. 5:**
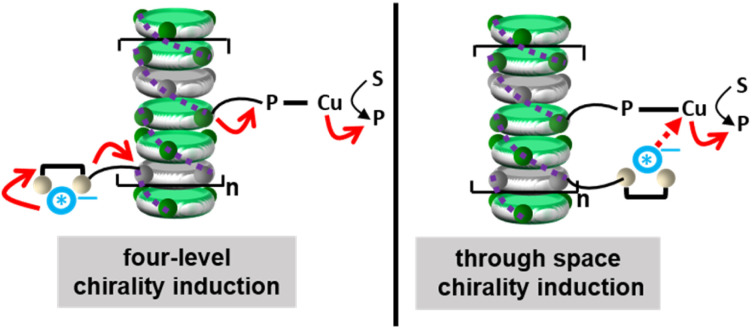
Possible modes of chirality induction in supramolecular helical TATA catalysts embedding a supramolecular “sergeant”. S: substrate, P: product.

The couple TATA *p*-U/TRIP thus constitutes the best tested supramolecular “sergeant” for the hydrosilylation reaction. Its selectivity is significantly higher than the one provided by TATA*, a covalent “sergeant” (8–9% ee, entries 10 and 11). It was previously found with BTA-based helical catalysts that the main difference in the displayed selectivities was related to the ability of the “sergeant” to intercalate efficiently into the stacks formed by the ligand.^17,66^ Herein, CD analyses suggest that both TATA *p*-U/TRIP and TATA* coassemble with the TATA “soldier” (Fig. S9†). Two possible pathways for chirality induction in catalytic TATA copolymers can thus be envisaged: (i) the anticipated induction of chirality “through bond” that allows to provide a chiral environment to the catalytic copper centre through a four-level induction of chirality from the chiral anion to the copper centre through the main chain of the supramolecular copolymer (as represented by the four red arrows in Fig. 5, left); the difference in selectivities between TATA *p*-U/TRIP and TATA* may arise from a different conformation induced to TATA PCF3 by the “sergeants”, (ii) an alternative induction of chirality through space because of the local proximity between the TRIP anion and the copper atom at the periphery of the TATA copolymers (Fig. 5, right); this mechanism may allow a better stereodiscrimination of the transition states explaining the superiority of TATA *p*-U/TRIP over TATA*.^67^ These pathways can actually constitute two limited structures; it can also be envisaged as contribution of both pathways for biasing the stereochemical outcome of the reaction with the supramolecular “sergeant”. Even though the overall enantioselectivities are significantly lower than those obtained with BTA-based helical catalysts^13,17,18,66,68^ under similar conditions, there is arguably room for further optimization notably through the screening of chiral anions of varied chemical natures,^69–71^ a strategy not accessible with conventional “sergeants”.

## Conclusions

We devised a new strategy for the induction of chirality in supramolecular copolymers of hydrogen-bonded triarylamine trisamide (TATA) monomers. Thanks to the orthogonal nature of the amide and (thio)urea moieties,^72,73^ the TRIP anion specifically binds the thiourea and urea functions of functionalized TATA monomers, thus furnishing a supramolecular chiral inducer that can be used as a “sergeant” for the construction of a variety of helically-biased TATA copolymers. Effective achiral TATA monomers (“soldiers”) encompass two TATA monomers functionalized with a phosphine unit and a TATA monomer with a different connectivity of the amide function. The present approach allows induction of chirality in pre-formed supramolecular copolymers *in situ*, without the need of a chiral monomer. For a benchmark catalytic reaction, the supramolecular “sergeant” provides higher enantioselectivity than a conventional covalent “sergeant,” thus reflecting the potential of this strategy for developing more efficient supramolecular helical catalysts. The modular and multi-component nature of this new type of supramolecular catalyst should enable the rapid screening of monomer and chiral anion combinations. Likewise, previous data in the literature suggest that TATA coassemblies can display very strong levels of chirality induction through the “sergeants-and-soldiers” effect.^11^ Increasing the performance of the supramolecular “sergeant”, notably by adding achiral TATA monomers playing the role of additives^13^ and designing switchable TATA catalysts^74^ are attractive extensions of this work.

## Author contributions

A. P. performed the experimental work, organized and arranged the data, and composed the first version of the manuscript. Q. S. and W. F. conducted preliminary data that led to the design of the (thio)urea-based TATA monomers. S. G. and J. L. discussed the concept of the project by bringing their expertise to the induction of chirality by means of chiral anions. L. B. contributed to the elaboration of the concept. M. R. secured funding, reproduced selected experimental data, supervised the project, analysed the data, and revised the manuscript.

## Conflicts of interest

There are no conflicts to declare.

## Supplementary Material

SC-016-D5SC02159F-s001

## Data Availability

The synthetic, analytical and catalytic data generated in this study are all provided in the ESI.†
