# Supplementary material for: Supramolecular “sergeants”: in situ and multi-level induction of chirality in helical assemblies of triarylamine trisamide monomers
Source: Chem Sci. 2025 Jul 10;16(32):14584–94. doi: 10.1039/d5sc02159f (PMC12272724; doi:10.1039/d5sc02159f)
Supplement: SC-016-D5SC02159F-s001 [file SC-016-D5SC02159F-s001.pdf]

Supplementary Information (SI) for Chemical Science.
This journal is © The Royal Society of Chemistry 2025

Supporting information

Supramolecular "sergeants": in situ and multi-level induction of chirality in helical assemblies of triarylamine trisamide monomers

Antoine Perennes,[a] Quentin Sallembien,[a] Weiwei Fang,[a] Stéphane Grass,[b] Jérôme Lacour,[b] Laurent Bouteiller[a] and Matthieu Raynal*[a]

a Sorbonne Université, CNRS, Institut Parisien de Chimie Moléculaire, 4 Place Jussieu, 75005 Paris (France) e-mail: matthieu.raynal@sorbonne-universite.fr.

b Department of Organic Chemistry, University of Geneva, Quai Ernest Ansermet 30, 1211 Geneva 4, Switzerland.

Supplementary Chart S1, Figures S1-S15 and Table S1 ______ 2

General methods ______ 20

Solutions for FT-IR and CD analyses ______ 22

Catalytic experiments ______ 23

Synthetic procedures ______ 24

Selected chiral GC traces (Figures S16-S17) ______ 35

NMR spectra (Figures S18-S58) ______ 36

References ______ 60

Supplementary Chart S1, Figures S1-S15 and Table S1

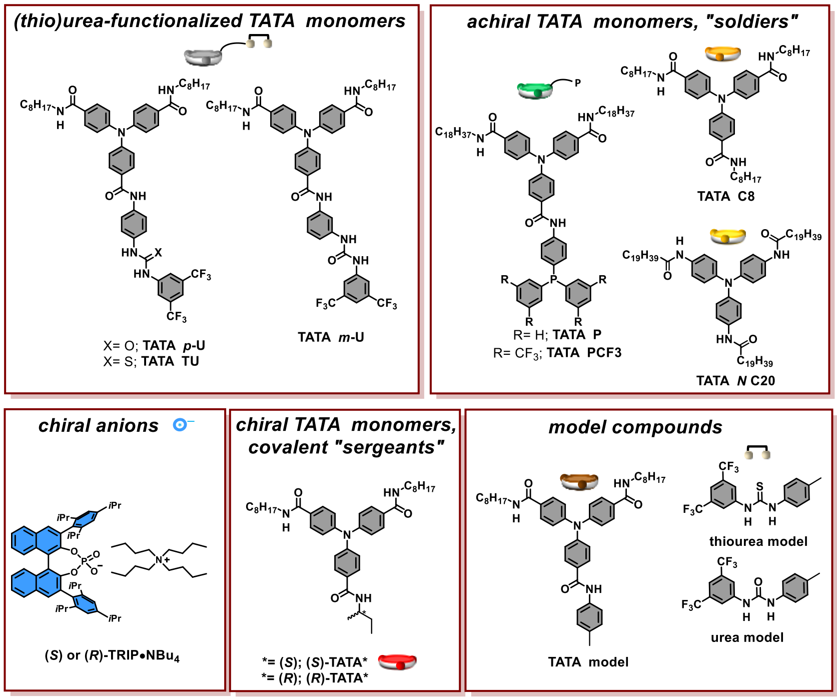

Chart S1. a) Chemical structures of TATA monomers, chiral anions, and model compounds used in this study.

Throughout this paper, the fraction of supramolecular "sergeants" initially introduced in the TATA mixture (fs), is defined as follows: fs= [(thio)urea based TATA monomer]/([(thio)urea based TATA monomer] + ["soldier"]), for which [(thio)urea based TATA monomer]= **TATA TU**, **TATA *m*-U** or **TATA *p*-U** and "soldier"= **TATA C8**, **TATA P**, **TATA PCF3** or **TATA *N* C20**. This is because in the sergeants-and-soldiers type experiments the concentrations in chiral anion and in (thio)urea based TATA monomer are identical. For the experiments concerning the in-situ chirality induction to **TATA TU** + **TATA P** (Figure 4), the results are reported as a function of the amount of chiral anion added relatively to **TATA TU**, the corresponding fs is estimated by dividing this value by 2 (considering that all TATA TU are bonded to the TRIP anion).

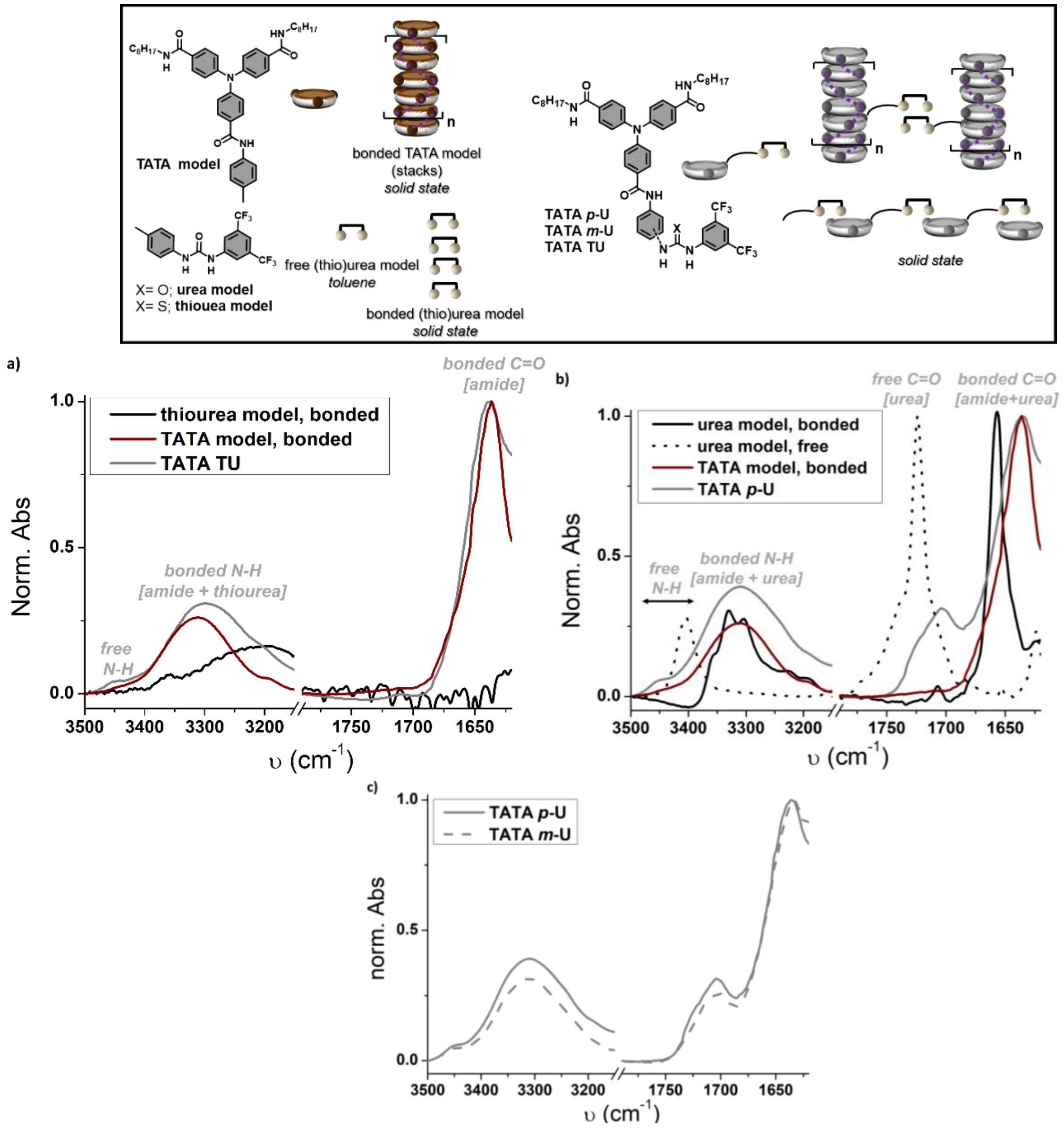

Figure S1. FT-IR analyses of **TATA TU** (a), **TATA *p*-U** (b) and compared FT-IR analyses of **TATA *p*-U** and **TATA *m*-U** (c) in the solid state. Comparison with the FT-IR spectra of bonded **TATA model** (solid state), bonded **thiourea model** (solid state), bonded **urea model** (solid state), and of free **urea model** (toluene, 2.9 mM). Zoom on the N─H and C=O regions. Top: schematic representation of the assemblies formed by **TATA model**, (thio)urea models and (thio)urea-functionalized TATA monomers in the solid state.

Interpretation: Assignment of the bands are made according to the literature[1] and the spectra of the models: free N─H (of amide): 3400─3450 cm^{-1}, bonded N─H (of amide to amide, case of **TATA model**): 3300 cm^{-1}, bonded N─H (of urea to urea, case of bonded **urea model**): 3300 cm^{-1} (several bands), bonded N─H (of thiourea, case of bonded **thiourea model**): 3200 cm^{-1}, free C=O (of urea, case of free **urea model**): 1720 cm^{-1}, bonded C=O (of urea to urea, case of bonded **urea model**): 1655 cm^{-1}, bonded C=O (of amide to amide, case of **TATA model**): 1638 cm^{-1}. FT-IR analyses of **TATA TU**, **TATA *p*-U** and **TATA *m*-U** reveal that both their amide and (thio)urea functions participate in the hydrogen-bonding network present in their homoassemblies. Possible aggregation modes are represented schematically above the spectra.

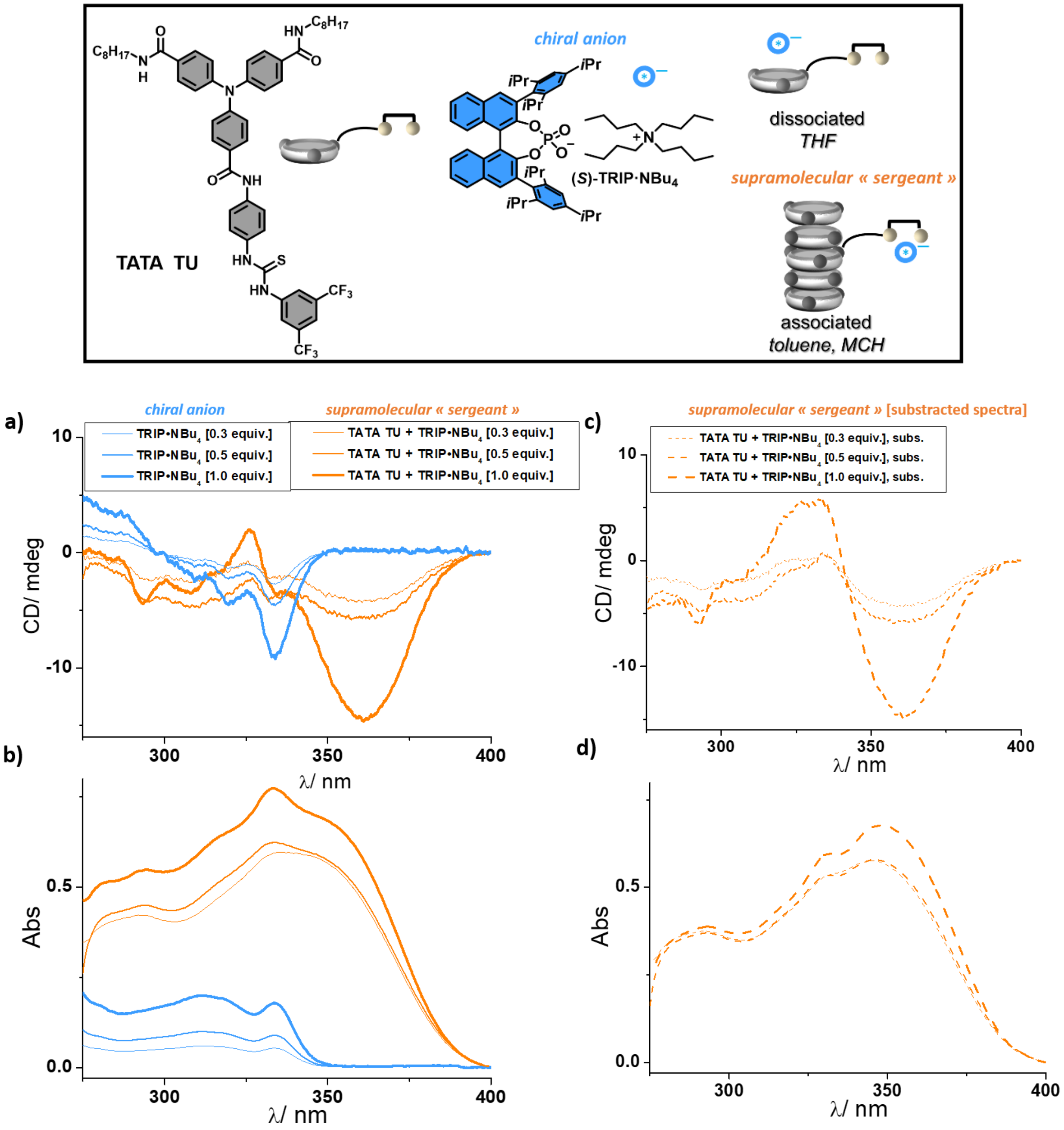

Figure S2. Characterization of the supramolecular "sergeant" by CD spectroscopy in toluene. a) CD analyses of the chiral anion alone (**(*S*)-TRIP•NBu$_4$**) and of the supramolecular "sergeant" formed by mixing **TATA TU** and **(*S*)-TRIP•NBu$_4$** in toluene. 1:0.3, 1:0.5, and 1:1 mixtures are recorded that contains the same amount of **TATA TU** (2.9 mM) and 0.87 mM (23%), 1.45 mM (33%) and 2.9 mM (50%) of **(*S*)-TRIP•NBu$_4$**, respectively. b) Corresponding UV-Vis analyses (of a). c) CD spectra (from a) for which the contribution from **(*S*)-TRIP•NBu$_4$** has been subtracted. d) Corresponding UV-Vis analyses (of c).

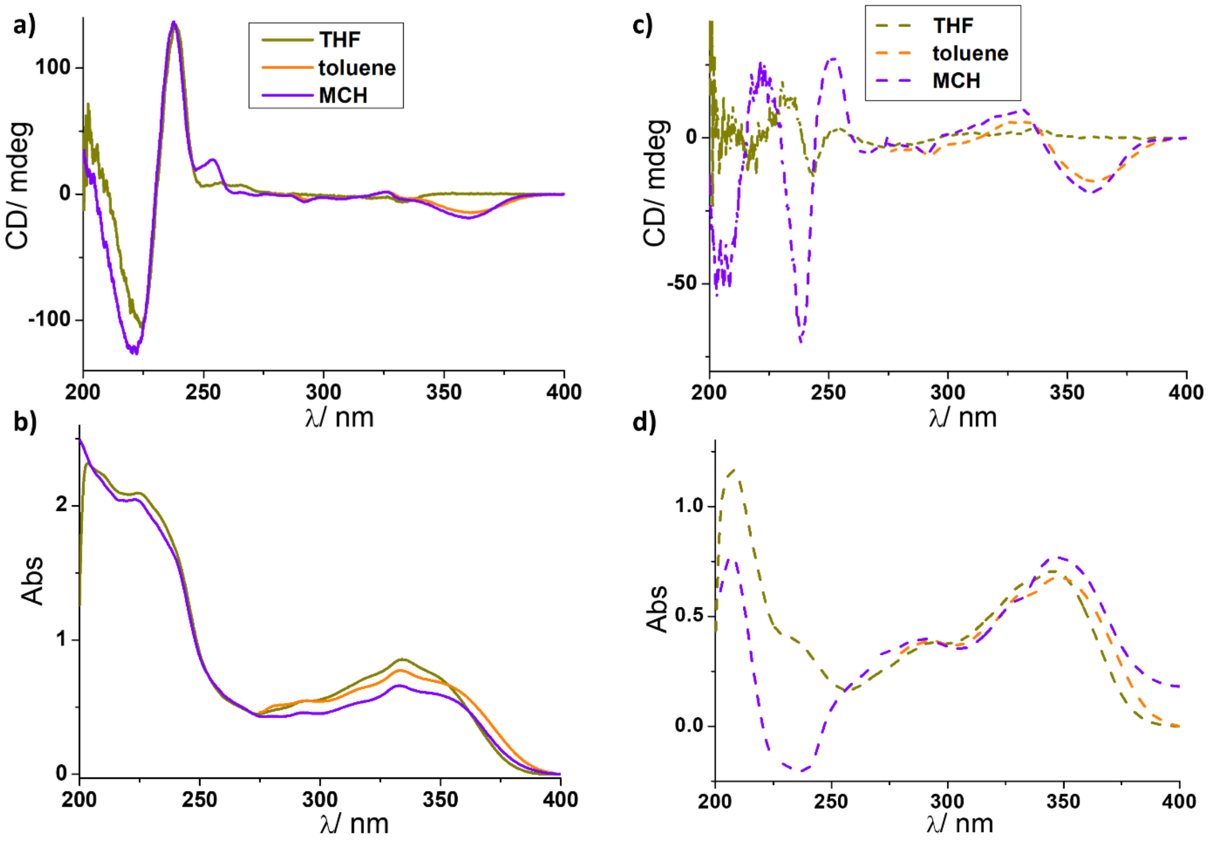

Figure S3. Characterization of the supramolecular "sergeant" by CD spectroscopy in various solvents. a) CD analyses of the supramolecular "sergeant" formed by mixing **TATA TU** and **(*S*)-TRIP•NBu$_4$** in THF, toluene and MCH (1:1 mixture, 5.8 mM total concentration). b) Corresponding UV-Vis analyses (of a). c) CD spectra (from a) for which the contribution of **(*S*)-TRIP•NBu$_4$** to the CD signal has been subtracted. d) Corresponding UV-Vis analyses (of c).

Interpretation: The supramolecular "sergeant" formed by mixing **TATA TU** and **(*S*)-TRIP•NBu$_4$** shows similar CD bands in toluene and MCH. In THF, no obvious induced CD band is detected (Figure S3c), corroborating the fact that **TATA TU** and **(*S*)-TRIP•NBu$_4$** are not associated in this solvent.

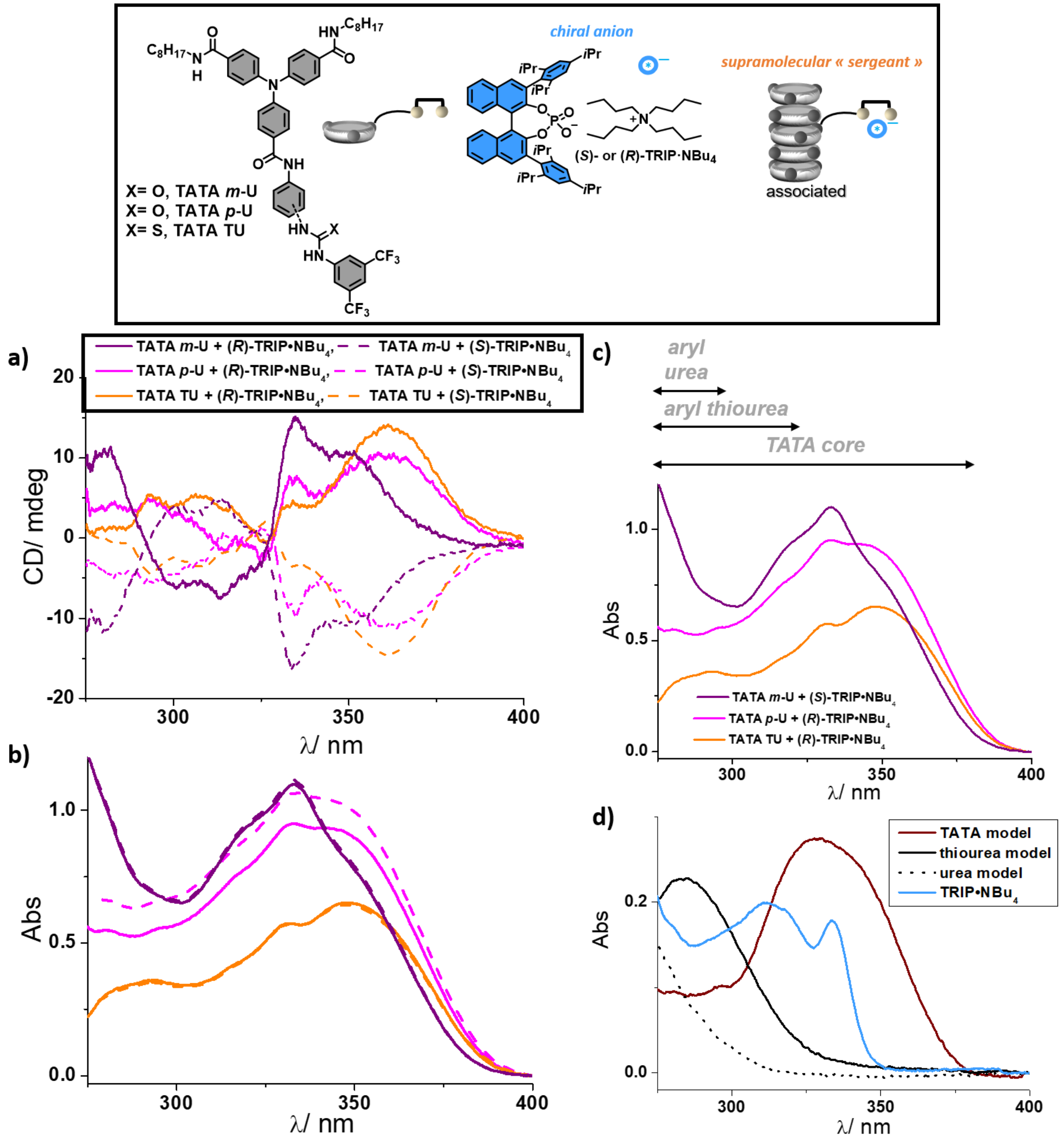

Figure S4. Characterization of the supramolecular "sergeants" by CD and UV-Vis spectroscopy. a) CD analyses of the supramolecular "sergeants" formed by mixing **TATA TU, TATA *p*-U** or **TATA *m*-U** and **(*R*)-** or **(*S*)-TRIP•NBu$_4$** in toluene (1:1 mixture, 5.8 mM total concentration). b) Corresponding UV-Vis analyses (of a). c) UV-Vis analyses of the supramolecular "sergeants" formed by mixing **TATA TU, TATA *p*-U** or **TATA *m*-U** and **(*R*)-TRIP•NBu$_4$** in toluene with regions related to the different chromophores according to UV-Vis analyses of model compounds in d. d) UV-Vis analyses of **(*R*)-TRIP•NBu$_4$** (2.9 mM) and of the different model compounds (2.9 mM) in toluene.

Interpretation: The different UV-Vis bands for the equimolar mixtures shown in Figure S4c have been assigned thanks to the UV-Vis spectra of the model compounds (Figure S4d). This comparison shows that the induced CD signals encompass bands related to the thiourea function, the urea function but also to the triarylamine trisamide (TATA) core.

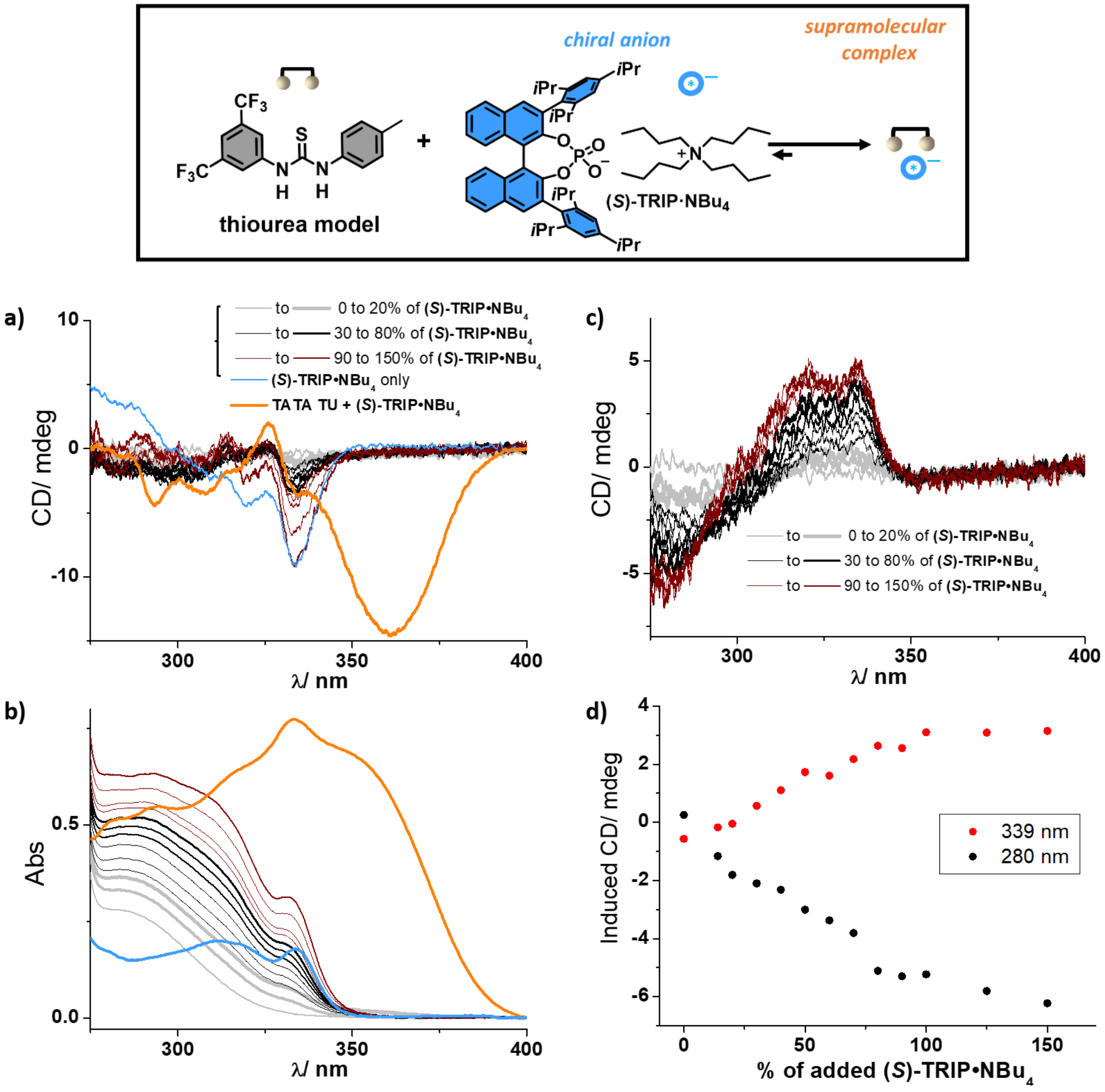

Figure S5. Supramolecular complex between the thiourea model and (*S*)-TRIP•NBu_4. a) CD analyses of mixtures between the thiourea model (2.9 mM) and **(*S*)-TRIP•NBu_4** (% of salt added relatively to the thiourea model: 14%, 20%, 30%, 40%, 50%, 60%, 70%, 80%, 90%, 100%, 125%, 150%) in toluene and comparison with the CD spectra obtained for **(*S*)-TRIP•NBu_4** (2.9 mM) and the supramolecular "sergeant" (TATA TU + **(*S*)-TRIP•NBu_4**, 1:1 mixture, 5.8 mM total concentration) in toluene. b) Corresponding UV-Vis analyses (of a). c) CD spectra (from a) for which the contribution of **(*S*)-TRIP•NBu_4** to the CD signal has been subtracted. This corresponds to the CD induced to the thiourea model in the supramolecular complex. d) Plot of the CD signal induced (ICD) to the thiourea model as a function of the % of added **(*S*)-TRIP•NBu_4**. The gradual increase of the ICD and the saturation of the ICD after addition of one equivalent of **(*S*)-TRIP•NBu_4** is consistent with the formation of 1:1 complex being the dominant species for all mixtures ($K_{complex} > 10^4$).

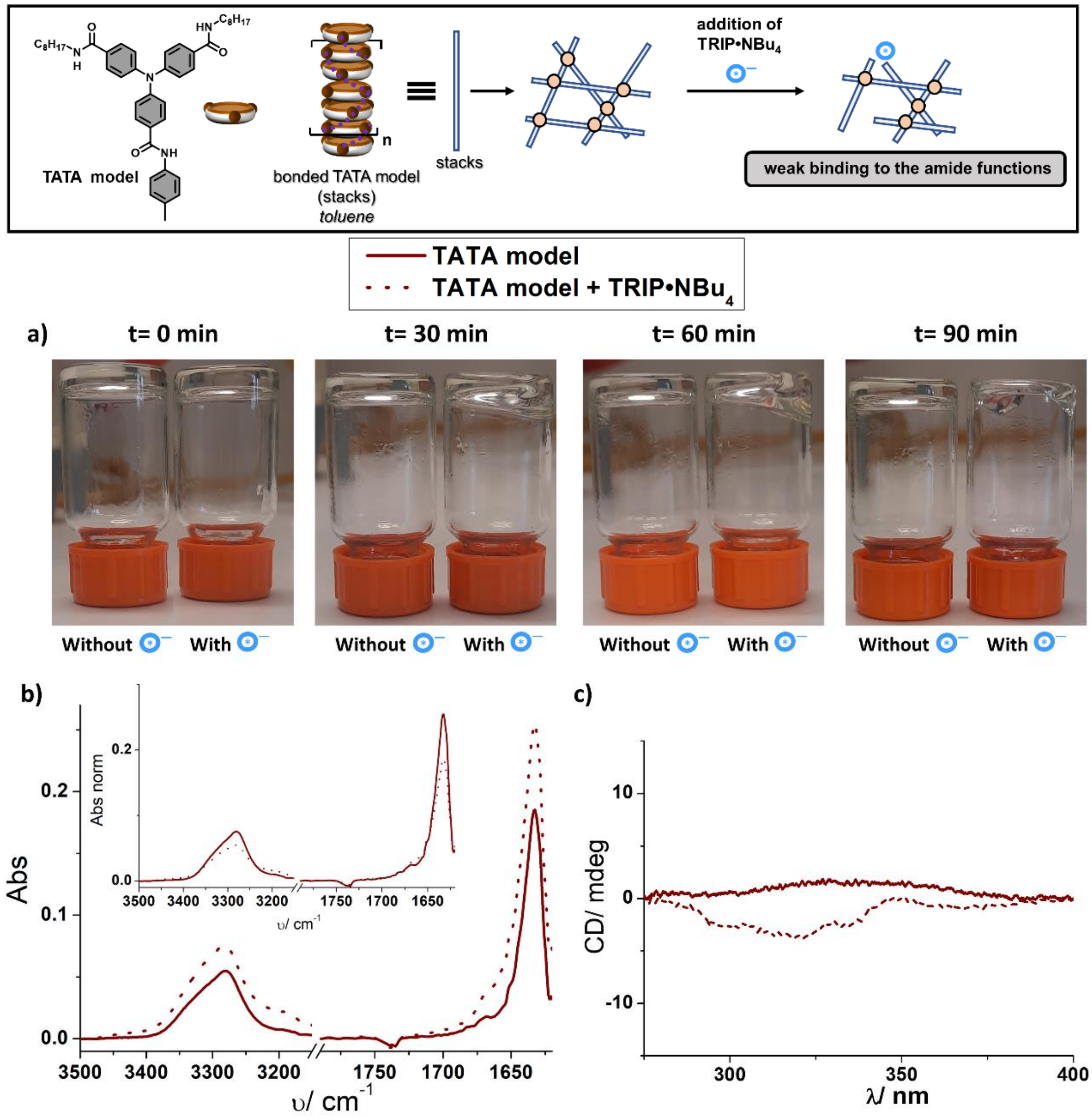

Figure S6. Interaction of the chiral anion (*R*)-TRIP•NBu$_4$ with stacks of TATA model. a) Pictures of the gel/viscous solution obtained for **TATA model** alone (2.9 mM) and its equimolar mixture with **(*R*)-TRIP•NBu$_4$** in toluene. b) FT-IR analyses of **TATA model** alone (2.9 mM) and its equimolar mixture with **(*R*)-TRIP•NBu$_4$** in toluene (inset: normalized spectra at the maxima of the C=O band). c) CD analyses of **TATA model** alone (2.9 mM) and of its equimolar mixture with **(*R*)-TRIP•NBu$_4$** in toluene. For the mixture, the contribution of **(*R*)-TRIP•NBu$_4$** to the CD signal has been subtracted.

Interpretation: TATA model forms long helical stacks in solution as deduced from: (i) FT-IR bands (νN-H= 3280 cm^{-1}, νC=O= 1638 cm^{-1}) characteristic of hydrogen-bonded TATA assemblies,[1,2] (ii) the fact that it forms a gel, probably as a consequence of entanglements between fibers (as represented schematically above the figure, the orange circles represent the entanglements). Addition of **(*R*)-TRIP•NBu$_4$** leads to a weaker gel, yet the solution remains viscous as can be seen from the pictures in Figure S6a. Likewise, no significant changes can be observed in the FT-IR spectrum of the mixture (Figure S6b). The lower intensity of the FT-IR spectrum observed for the mixture is attributed to a better filling of the cell by the less viscous solution. In addition, CD spectra shown in Figure S6c indicate that induction of chirality through chain capping does not occur to a significant extent.

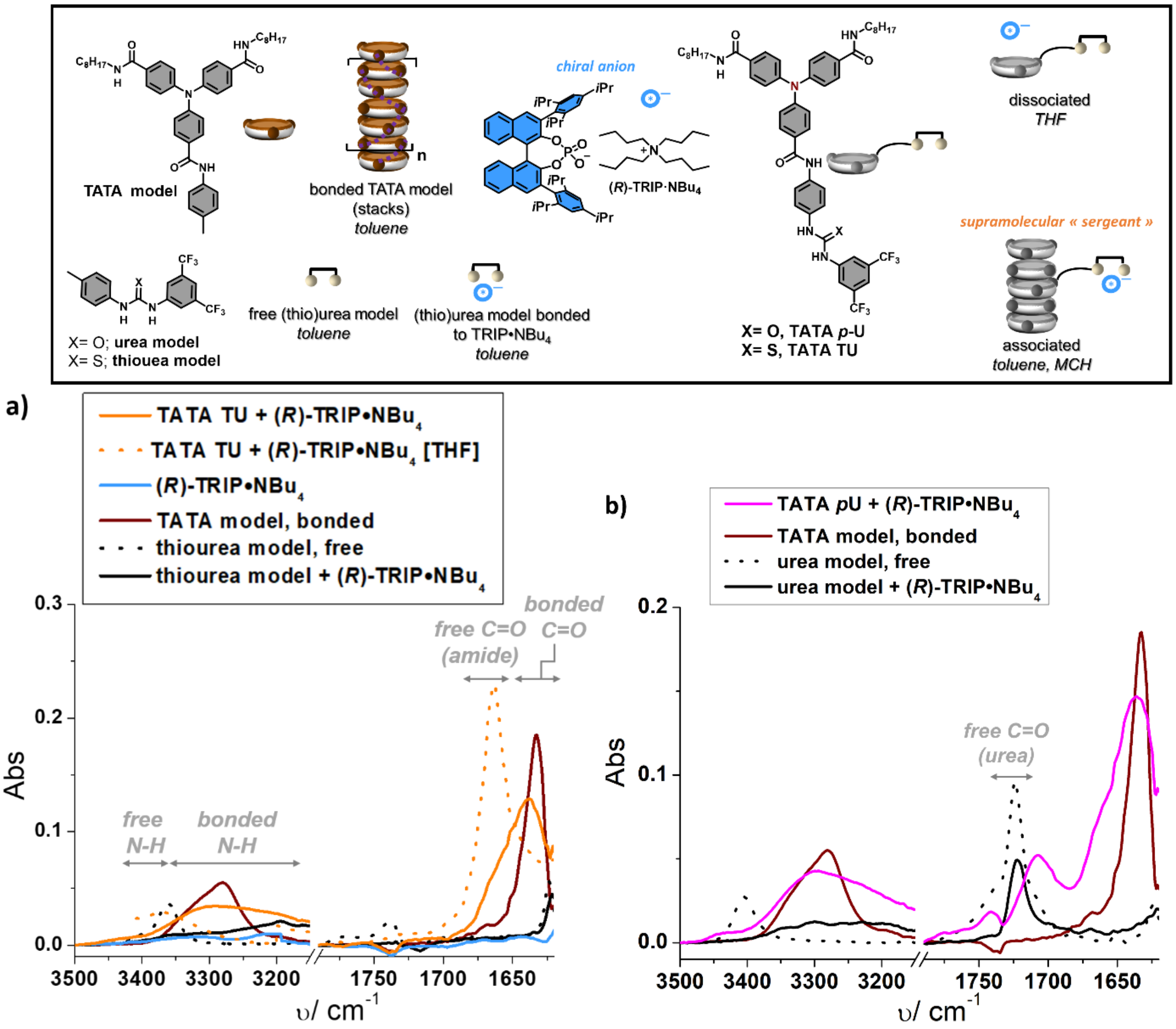

Figure S7. Characterization of the supramolecular "sergeants" by FT-IR. a) FT-IR analyses of **(*R*)-TRIP•NBu$_4$** alone (2.9 mM), TATA model (2.9 mM), thiourea model alone (2.9 mM), the mixture between thiourea model and **(*R*)-TRIP•NBu$_4$** (2.9 mM + 2.9 mM) and of the mixture between **TATA TU** and **(*R*)-TRIP•NBu$_4$** (2.9 mM + 2.9 mM) in toluene and of the mixture between **TATA TU** and **(*R*)-TRIP•NBu$_4$** (2.9 mM + 2.9 mM) in THF. b) FT-IR analyses of TATA model (2.9 mM), urea model alone (2.9 mM), the mixture between urea model and **(*R*)-TRIP•NBu$_4$** (2.9 mM + 2.9 mM) and of the mixture between **TATA *p*-U** and **(*R*)-TRIP•NBu$_4$** (2.9 mM + 2.9 mM) in toluene.

Interpretation: The different FT-IR bands of the supramolecular "sergeant" can be assigned thanks to the comparison between the different spectra or the literature (see also Figure S1)**:** free N─H (of amide): 3400─3450 cm^{-1}, free N─H (of urea and thiourea, cases of **thiourea model** and **urea model** in toluene): 3380─3420 cm^{-1}, bonded N─H (of amide to amide, case of **TATA model**): 3300 cm^{-1}, bonded N-H (of thiourea or urea to TRIP, cases of mixtures of **thiourea model** or **urea model** and TRIP in toluene): 3200-3300 cm^{-1} (broad band), free C=O (of urea, case of **urea model** in toluene): 1720 cm^{-1}, free C=O (of amide, case of the mixture between **TATA TU** and TRIP in THF): 1670 cm^{-1}, bonded C=O (of amide to amide, case of **TATA model**): 1638 cm^{-1}. Comparison with simulated spectra (see Figure 1) indicates that the "supramolecular sergeants" probably exist under the form of short stacks (or stacks with some disorder) with the TRIP anion specifically bonded to the thiourea or urea function of **TATA TU** and **TATA *p*-U**, respectively (as represented schematically above the figure).

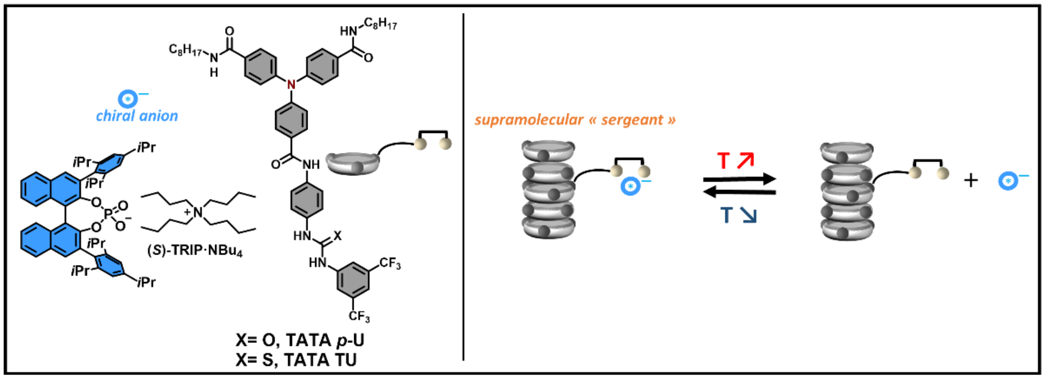
chiral anion
(S)-TRIP·NBu4
X= O, TATA p-U
X= S, TATA TU
supramolecular « sergeant »
T ↗
T ↘

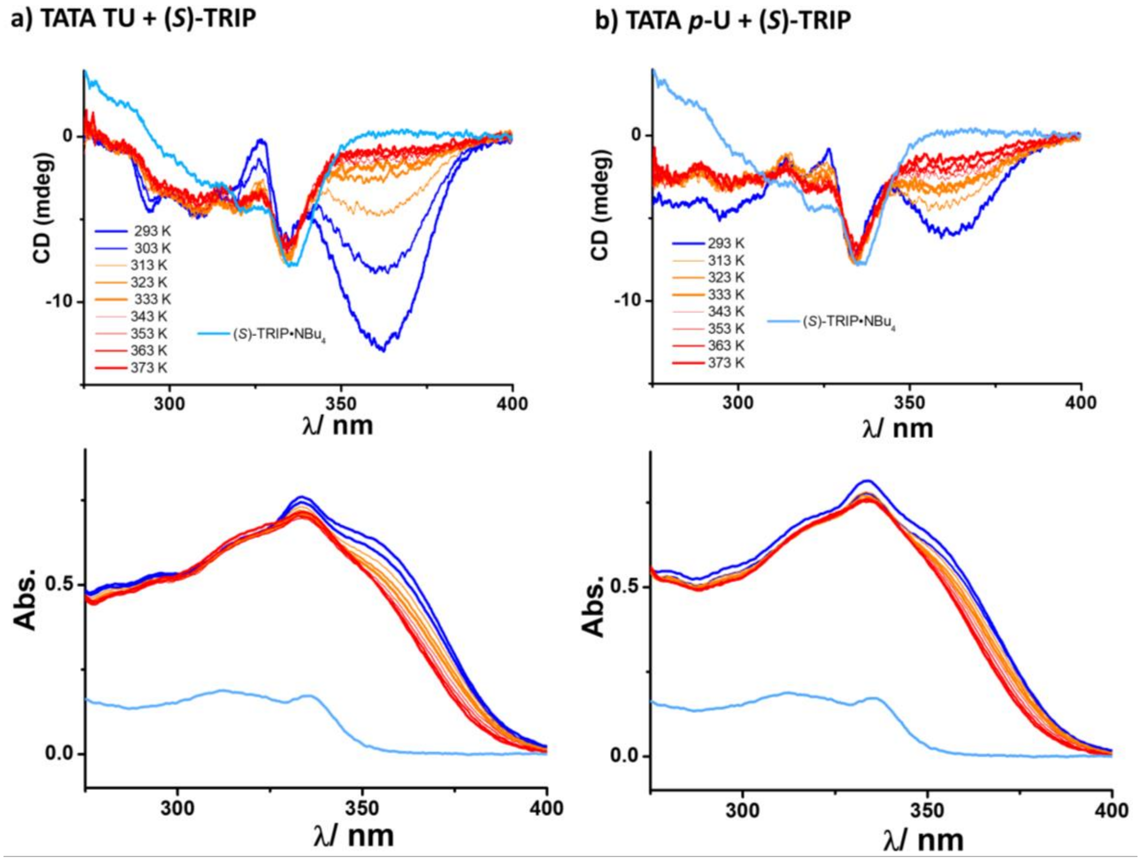
a) TATA TU + (S)-TRIP
b) TATA p-U + (S)-TRIP
CD (mdeg)
(S)-TRIP•NBu4
293 K
303 K
313 K
323 K
333 K
343 K
353 K
363 K
373 K
λ/ nm
Abs.

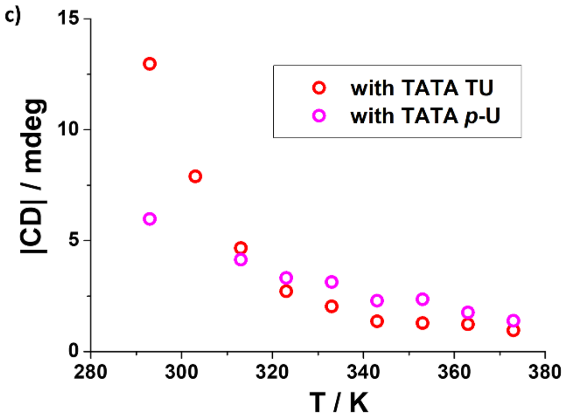
c)
with TATA TU
with TATA p-U
|CD| / mdeg
T / K

Figure S8. Stability of the supramolecular "sergeants". a) CD (top) and UV-Vis (bottom) analyses of the supramolecular "sergeant" **(TATA TU + (*S*)-TRIP•NBu₄**, 1:1 mixture, 5.8 mM total concentration) in toluene at different temperatures between 293 K and 393 K. The CD spectrum of **(*S*)-TRIP•NBu₄** is shown for comparison of the spectrum of the mixture at 393 K. b) CD (top) and UV-Vis (bottom) analyses of the supramolecular "sergeant" **(TATA *p*-U + (*S*)-TRIP•NBu₄**, 1:1 mixture, 5.8 mM total concentration) in toluene at different temperatures between 293 K and 393 K. The CD spectrum of **(*S*)-TRIP•NBu₄** is shown for comparison of the spectrum of the mixture at 393 K. c) Plot of the CD intensity at 362.3 nm as a function of the temperature for the two supramolecular "sergeants".

Note: New solutions have been prepared to conduct these experiments relatively to data reported in Figures S2 and S4. The CD spectrum at 293 K for the supramolecular "sergeant" composed of **TATA TU** + **(*S*)-TRIP•NBu₄** is virtually identical to the one reported in these Figures. However, the CD spectrum at 293 K for the supramolecular "sergeant" composed of **TATA *p*-U** + **(*S*)-TRIP•NBu₄** exhibits a lower intensity ($|CD|_{362}$= 6.0 mdeg versus 9.8 mdeg in Figure S4). We attribute this difference to the fact that some precipitate tends to form with time from the solution containing this "sergeant" which may impede full reproducibility of the CD analysis.

Interpretation: For both supramolecular "sergeants", the CD band at ca. 360 nm induced by the chiral anion, i.e. the induced CD (ICD) band, gradually decreases when the temperature increases. The CD spectra at 393 K resemble those of the chiral anion; differences may arise from the fact that some aggregates still subsist at this temperature as can be deduced from residual intensity in the ICD band at this temperature (plot in c). Overall, these data indicate that the supramolecular "sergeants" gradually dissociate upon heating. The initial CD intensity is higher for "sergeant" derived from **TATA TU**, which suggests a stronger interaction between the chiral anion and the thiourea function.

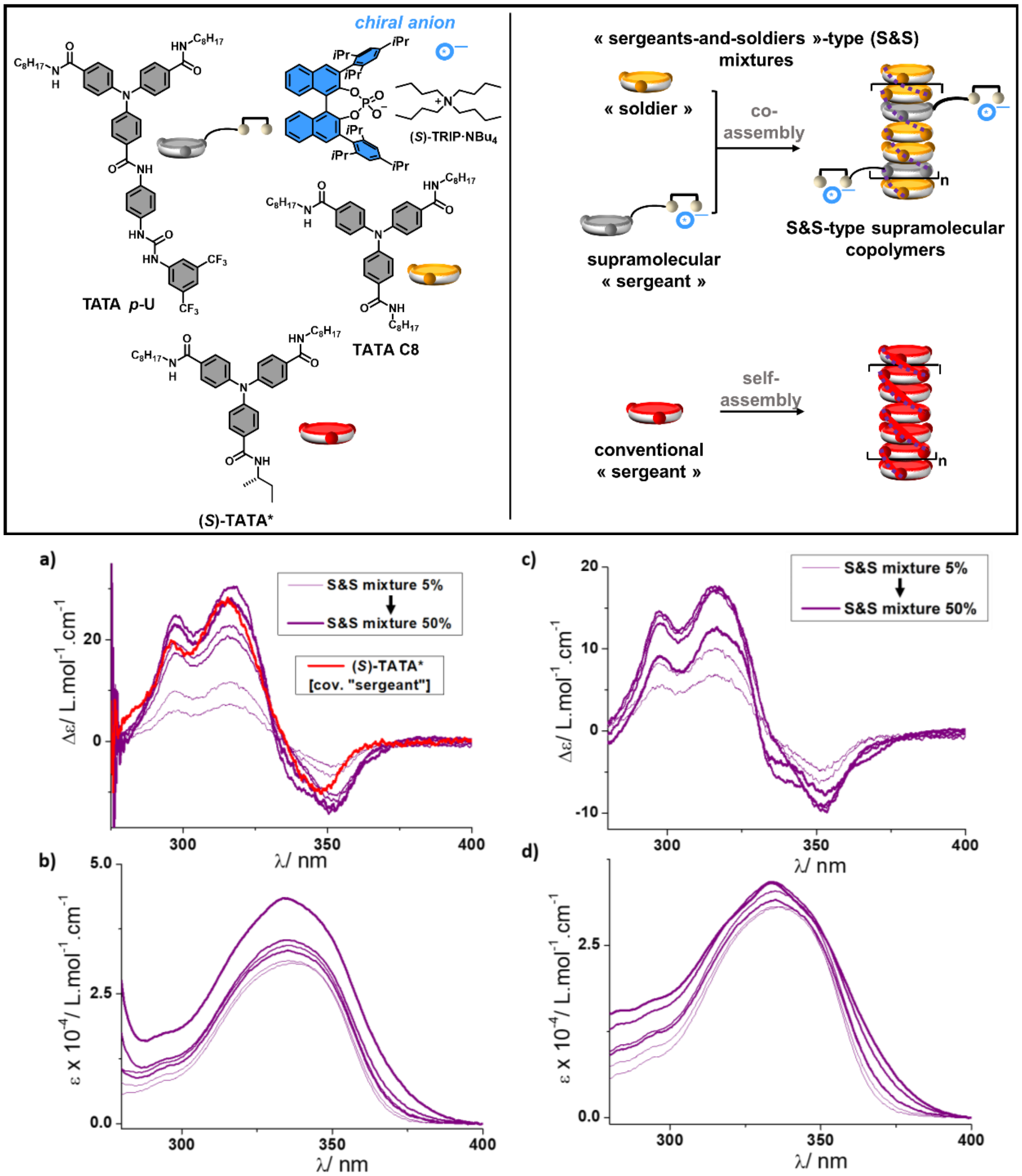

Figure S9. Induction of chirality to TATA C8 with TATA *p*-U + (*S*)-TRIP•NBu$_4$. a) CD analyses of the "sergeants-and-soldiers"- (S&S) type mixtures obtained by mixing **TATA C8** (the "soldier", 0.2 mM) with either various amounts of the supramolecular "sergeant" (**TATA *p*-U** + **(*S*)-TRIP•NBu$_4$**, 1:1 ratio, fs= 5%, 10%, 15%, 20%, 40%, 50%) or 0.2 mM of the covalent "sergeant" **(*S*)-TATA*** (f_s= 50%) in toluene. Molar CD values are obtained by subtracting the contribution of the supramolecular "sergeant" and by considering only the concentration of **TATA C8** for the remaining CD signal. b) Corresponding UV/Vis spectra (to a). c) CD spectra of the same mixtures but molar CD values are obtained from the pristine CD spectra by considering the total concentration in TATA monomers (**TATA C8** + the supramolecular "sergeant"). d) Corresponding UV/Vis spectra (to c).

Interpretation: CD spectra, obtained by subtracting the contribution of the supramolecular "sergeant" for each mixture, have similar shape than the mixture embedding the conventional covalent "sergeant" **(*S*)-TATA***. In addition, CD spectra of the mixtures containing 40% and 50% of supramolecular "sergeant" are very close in shape and intensity to the CD spectrum of the mixture with **(*S*)-TATA***: this corroborates the formation of homochiral copolymers.

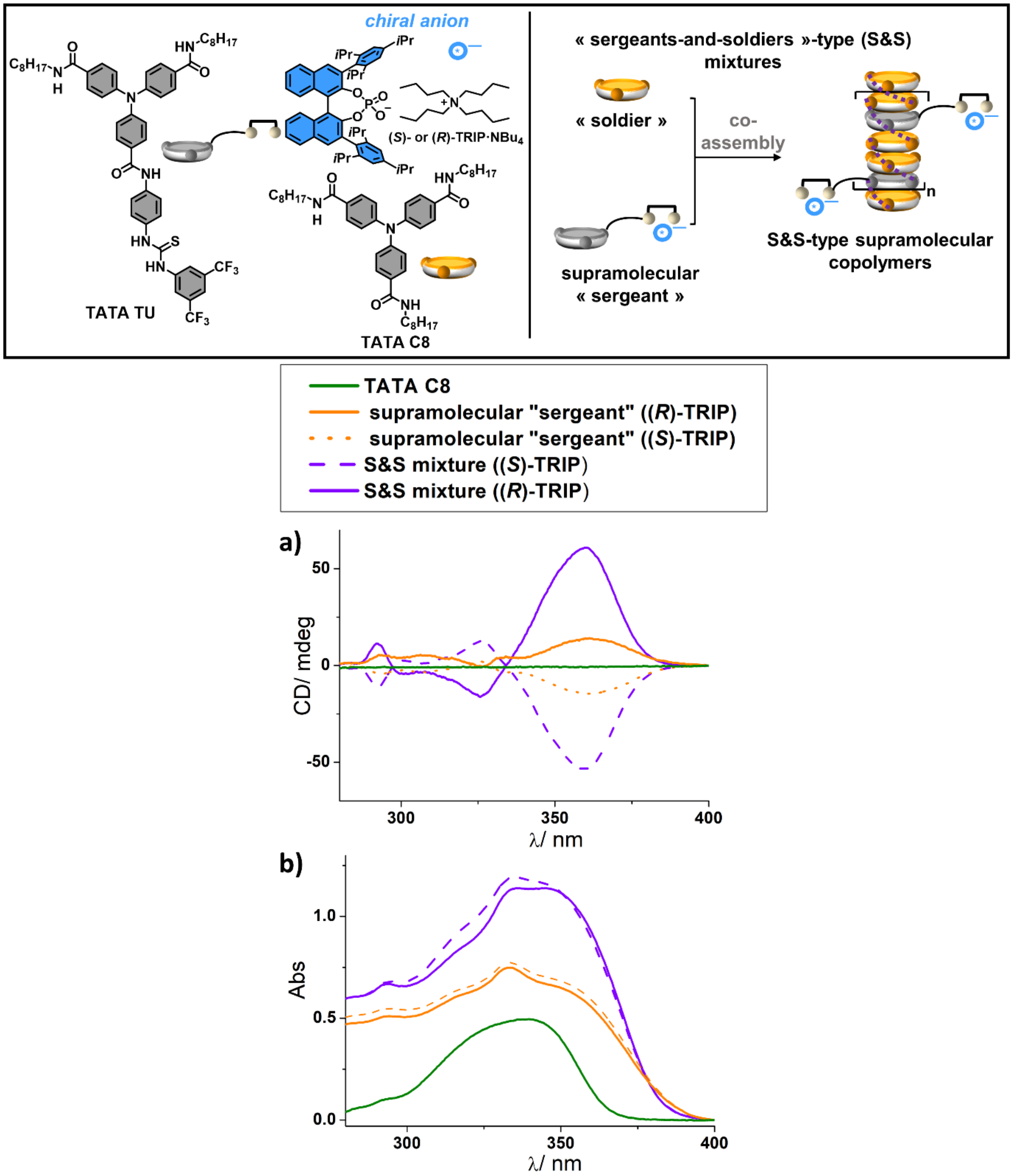

Figure S10. Induction of chirality to TATA C8 with TATA TU + TRIP•NBu$_4$. a) CD analyses of the “sergeants-and-soldiers”-type mixtures obtained by mixing **TATA C8** (the “soldier”, 2.9 mM) with the supramolecular “sergeants” (**TATA TU** + **(*R*)-TRIP•NBu$_4$** or **(*S*)-TRIP•NBu$_4$**, 1:1 ratio, fs= 50%, 2.9 mM) in toluene. The CD spectra of **TATA C8** alone (2.9 mM) and of the supramolecular “sergeants” (2.9 mM) are shown for comparison. b) Corresponding UV/Vis spectra (to a).

Interpretation: The different shape and higher intensity of the CD spectra of the “sergeants-and-soldiers”-type mixtures relatively to the those of the supramolecular “sergeants” alone corroborate the formation of copolymers with a preferred handedness.

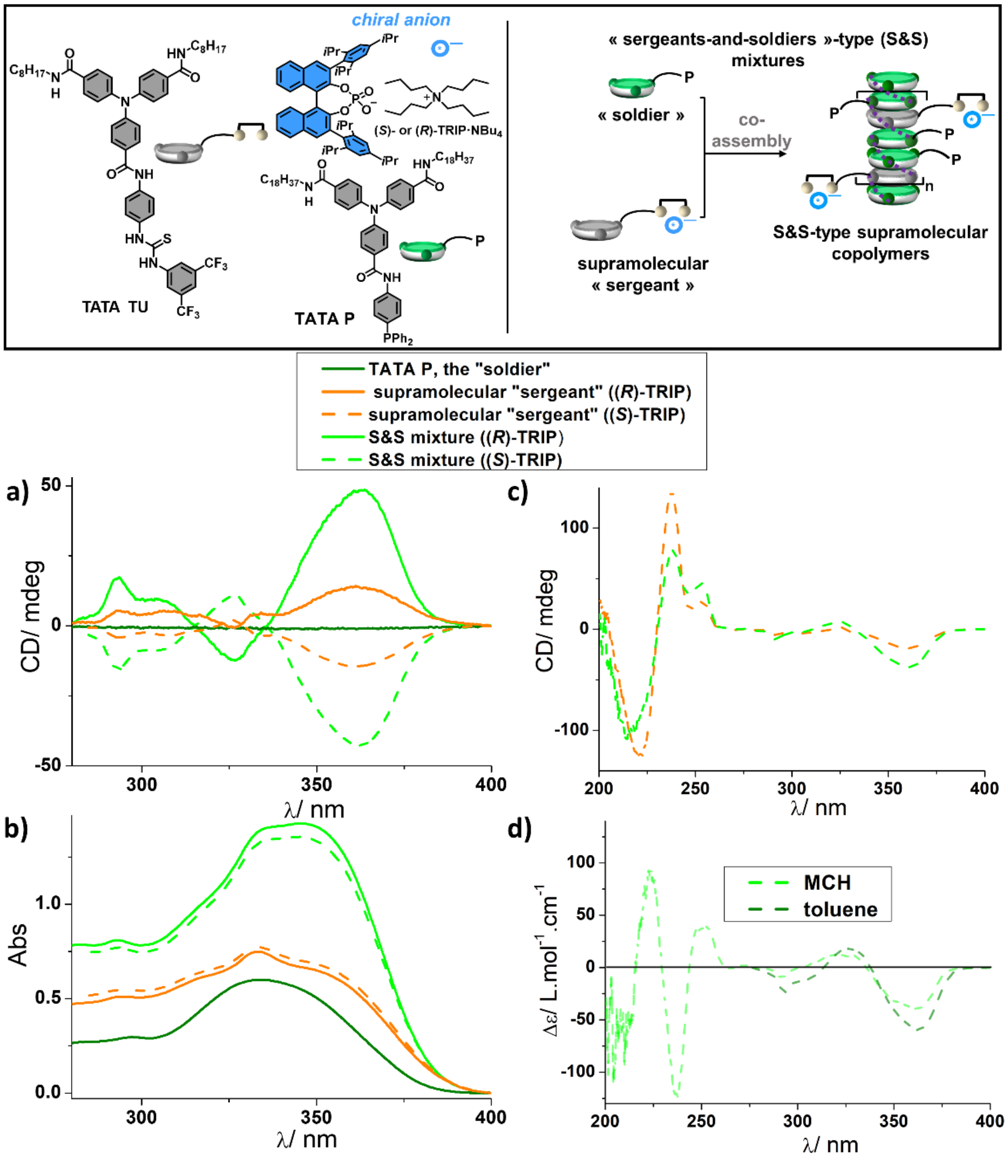

Figure S11. Induction of chirality to TATA P with TATA TU + TRIP•NBu$_4$. a) CD analyses of the "sergeants-and-soldiers"-type mixtures obtained by mixing **TATA P** (the "soldier", 2.9 mM) with the supramolecular "sergeants" (**TATA TU** + **(*R*)-TRIP•NBu$_4$** or **(*S*)-TRIP•NBu$_4$**, 1:1 ratio, fs= 50%, 2.9 mM) in toluene. The CD spectra of **TATA P** alone (2.9 mM) and of the supramolecular "sergeants" (2.9 mM) are shown for comparison. b) Corresponding UV/Vis spectra (to a). c) CD analyses of the "sergeants-and-soldiers" mixture obtained by mixing **TATA P** (the "soldier", 2.9 mM) with the supramolecular "sergeants" (**TATA TU** + **(*S*)-TRIP•NBu$_4$**, 1:1 ratio, fs= 50%, 2.9 mM) in MCH. The CD spectrum of the supramolecular "sergeant" (2.9 mM) is shown for comparison. d) Comparison of the CD spectra of the "sergeants-and-soldiers" mixture (**TATA P** +**TATA TU** + **(*S*)-TRIP•NBu$_4$**) in toluene and MCH. The contribution of the supramolecular "sergeant" has been subtracted.

Interpretation: The different shape and higher intensity of the CD spectra of the "sergeants-and-soldiers"-type mixtures relatively to the those of the supramolecular "sergeants" alone corroborate the formation of copolymers with a preferred handedness. Additional bands specific to the copolymers are detected in MCH. The lower intensity of the CD bands above 300 nm in this solvent, suggests a lower extent of chirality induction relatively to toluene.

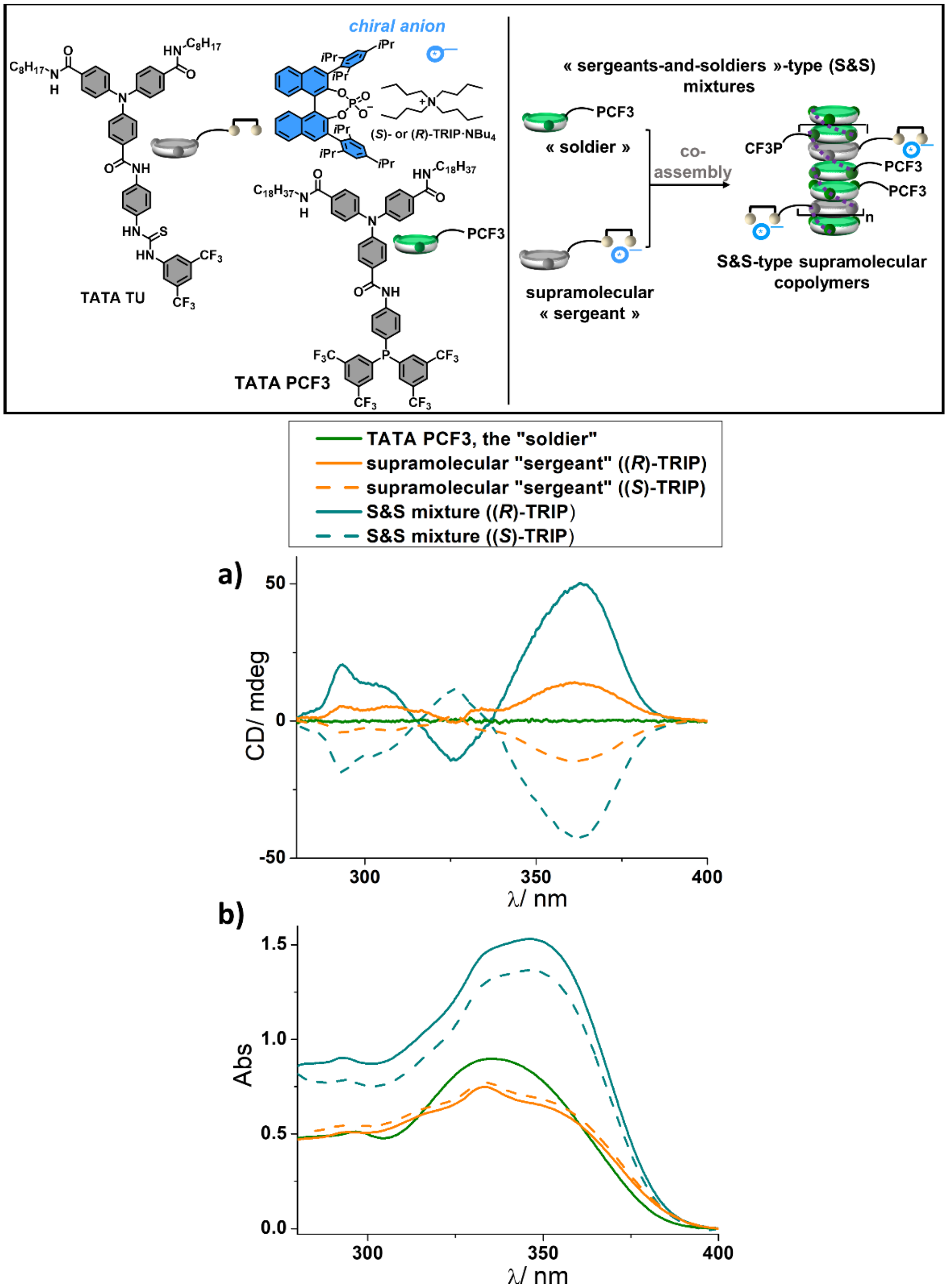

Figure S12. Induction of chirality to TATA PCF3 with TATA TU + TRIP•NBu_4. a) CD analyses of the "sergeants-and-soldiers"-type mixtures obtained by mixing **TATA PCF3** (the "soldier", 2.9 mM) with the supramolecular "sergeants" (**TATA TU + (*R*)-TRIP•NBu_4** or **(*S*)-TRIP•NBu_4**, 1:1 ratio, fs= 50%, 2.9 mM) in toluene. The CD spectra of **TATA PCF3** alone (2.9 mM) and of the supramolecular "sergeants" (2.9 mM) are shown for comparison. b) Corresponding UV/Vis spectra (to a).

Interpretation: The different shape and higher intensity of the CD spectra of the "sergeants-and-soldiers"-type mixtures relatively to the those of the supramolecular "sergeants" alone corroborate the formation of copolymers with a preferred handedness.

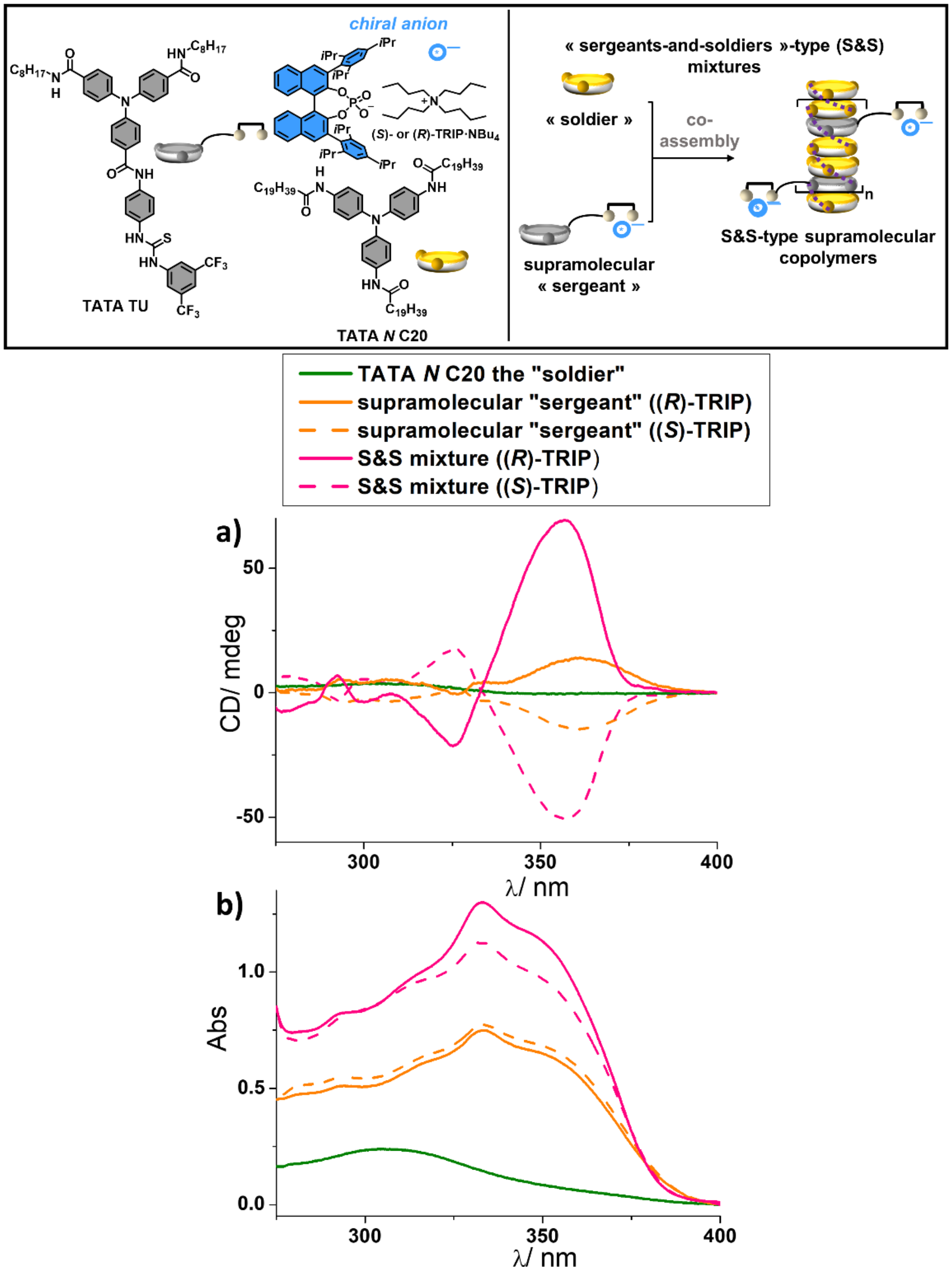

Figure S13. Induction of chirality to TATA *N* C20 with TATA TU + TRIP•NBu$_4$. a) CD analyses of the "sergeants-and-soldiers"-type mixtures obtained by mixing **TATA *N* C20** (the "soldier", 2.9 mM) with the supramolecular "sergeant" (**TATA TU** + **(*R*)-TRIP•NBu$_4$** or **(*S*)-TRIP•NBu$_4$**, 1:1 ratio, fs= 50%, 2.9 mM) in toluene. The CD spectra of **TATA *N* C20** alone (2.9 mM) and of the supramolecular "sergeants" (2.9 mM) are shown for comparison. b) Corresponding UV/Vis spectra (to a).

Interpretation: The different shape and higher intensity of the CD spectra of the "sergeants-and-soldiers"-type mixtures relatively to the those of the supramolecular "sergeants" alone corroborate the formation of copolymers with a preferred handedness.

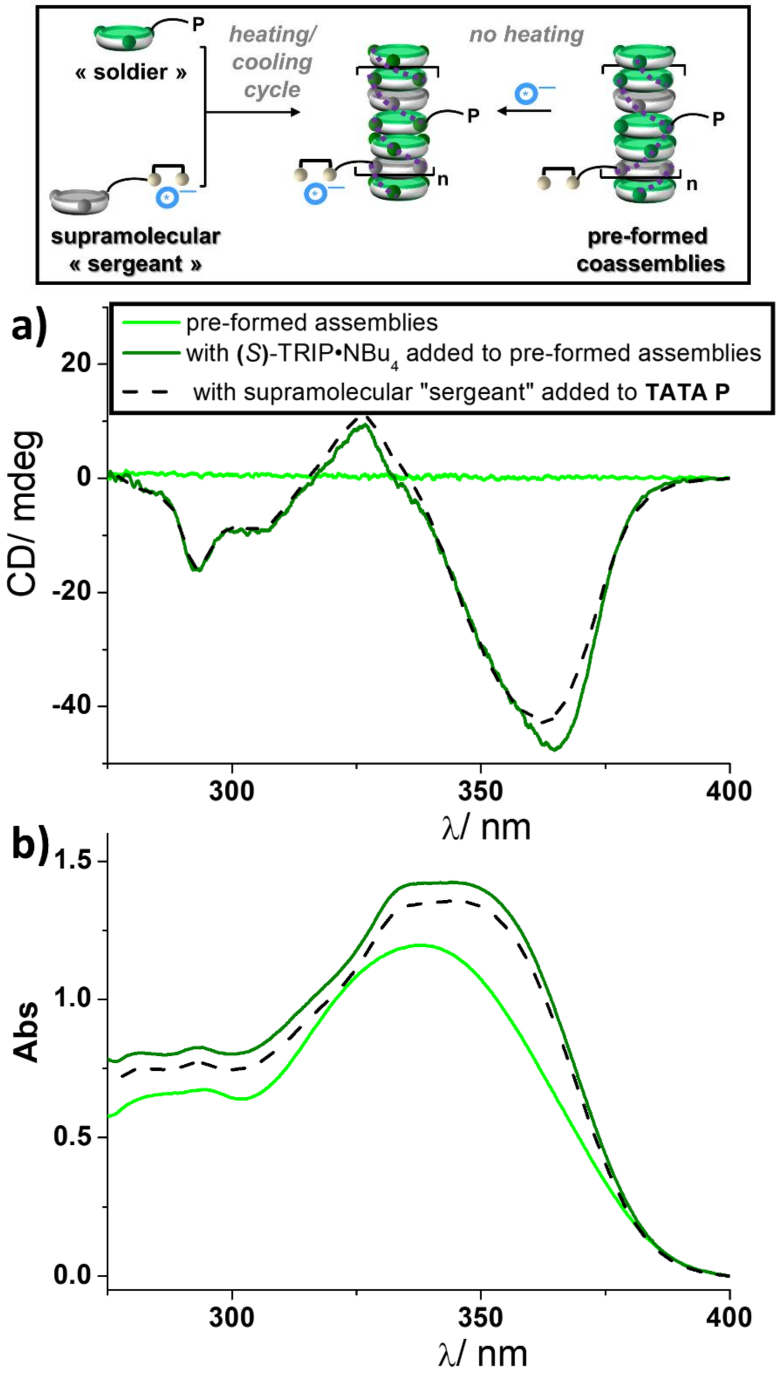

Figure S14. Different routes for chirality induction into TATA copolymers. a) CD spectra of the "sergeants-and-soldiers"-type copolymers composed of **TATA P** as "soldier" (2.9 mM) and of **TATA TU** + **(*S*)-TRIP•NBu$_4$** as the supramolecular "sergeant" (1:1 ratio, 2.9 mM) in toluene generated either by addition of the supramolecular "sergeant" to **TATA P** to or by addition of **(*S*)-TRIP•NBu$_4$** to pre-formed coassemblies of **TATA P** and **TATA TU**. For the latter, addition of the chiral inducer was done at room temperature and the spectrum was recorded ca. three minutes after mixing (no heating). b) Corresponding UV/Vis spectra (to a).

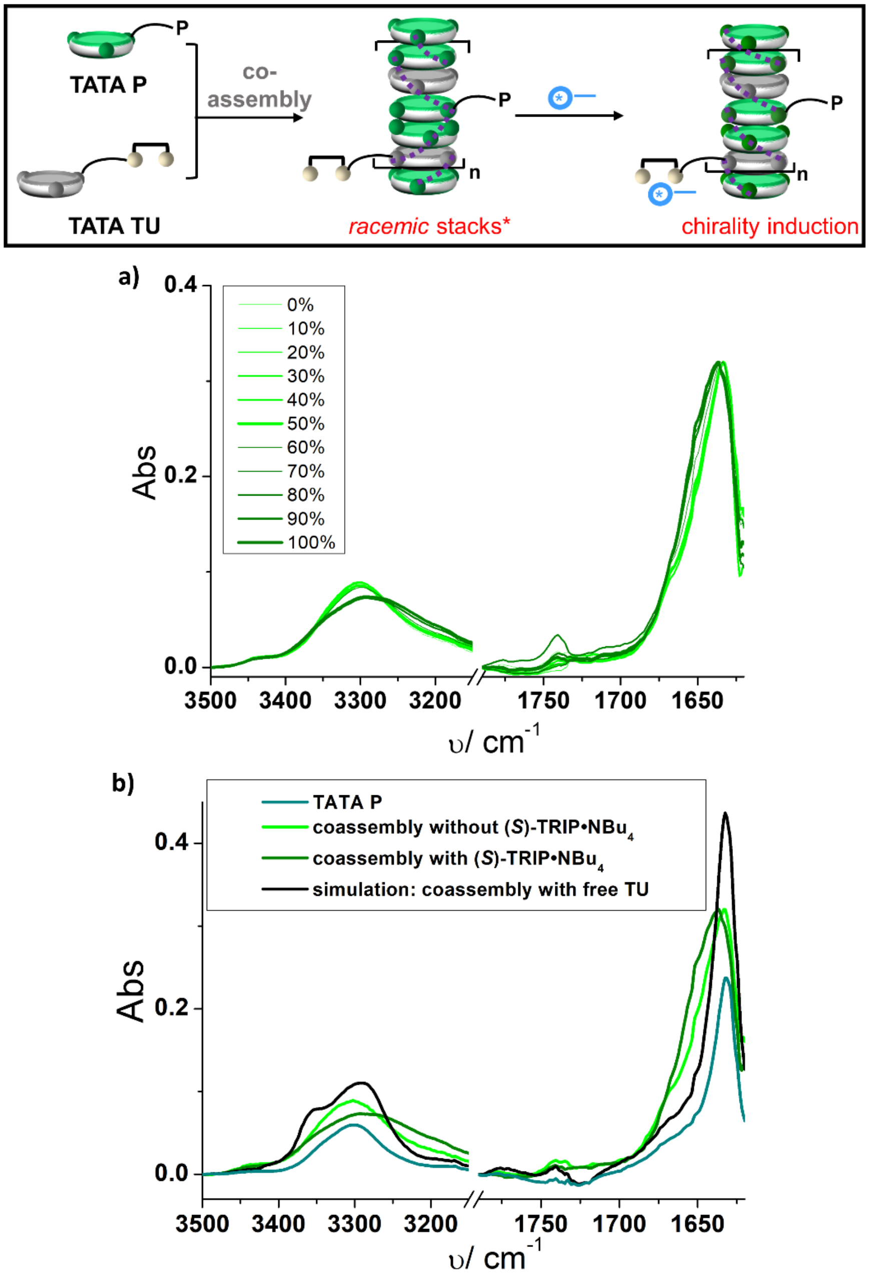

Figure S15. Chirality induction to TATA copolymers. a) FT-IR spectra of the "sergeants-and-soldiers"-type copolymers obtained by adding **(*S*)-TRIP•NBu$_4$** (0-100% relatively to **TATA TU**, 0-2.9 mM) to pre-formed copolymers between **TATA P** (2.9 mM) and **TATA TU** (2.9 mM) in toluene. b) FT-IR spectra of **TATA P** (2.9 mM), of the mixture **TATA P + TATA TU** (2.9 + 2.9 mM), and of the mixture **TATA P + TATA TU + (*S*)-TRIP•NBu$_4$** (2.9 + 2.9 + 2.9 mM). The spectrum obtained by simulating coassembly of **TATA P + TATA TU** with free thiourea function is also shown in black (see the General Methods section).

Interpretation: FT-IR analyses reveal two states: one for copolymers with no significant induction of chirality to **TATA P** and one for copolymers with induction of chirality to **TATA P** as deduced from CD analyses in Figure 4. The simulated spectrum indicates that the thiourea functions are not actually free in the pristine copolymer but are probably bonded to each other's either intramolecularly (in single helices) or intermolecularly (in bundled helices).

Table S1. Evaluation of the TATA ligands in the catalytic reaction.

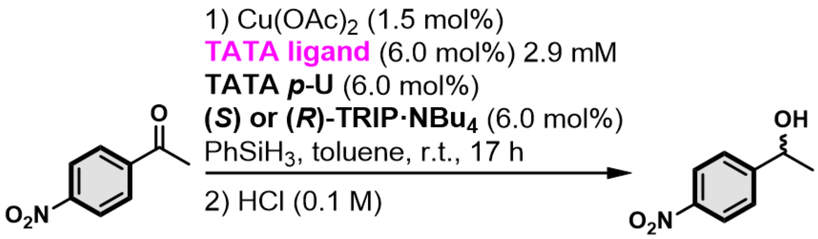

entry	TATA ligand	TRIP•NBu_4	conversion	*ee*±2 (%)
**1**	**TATA P**	***S***	**83 %**	**–13**
**2**	**TATA P**	***R***	**92 %**	**+13**
**3**	**TATA PCF3**	***S***	**99 %**	**–35**
**4**	**TATA PCF3**	***R***	**98 %**	**+30**

General methods

Synthetic procedures: Urea and thiourea models were obtained by reaction of *p*-toluidine with 3,5-bis(trifluoromethyl)phenyl isocyanate and 3,5-bis(trifluoromethyl)phenyl isothiocyanate, respectively. *N*-eicosanoic acid, diphenyl ether, tetrabutylammonium chloride, 4-nitroacetophenone, $Cu(OAc)_2$, DMAP, HOBt, octylamine, potassium hydroxide, potassium carbonate, copper iodide, copper powder, oxalyl chloride, methylcyclohexane (MCH), (*S*)-2-aminobutane, and anhydrous DMF were acquired from Sigma Aldrich. Methyl 4-iodo-benzoate, and (*R*)-2-aminobutane were obtained from Alfa Aesar. EDC•HCl and (*S*) and (*R*)-3,3'-bis(2,4,6-triisopropylphenyl)-1,1'-binaphthyl-2,2'-diyl hydrogen phosphate (**(*S*)-** and **(*R*)-TRIP**) were purchased from ABCR. Octadecylamine and *p*-toluidine were provided from Acros Organics, and methyl 4-amino-benzoate and 4,4',4''-triaminotriphenylamine were bought from Fluorochem. 3,5-bis(trifluoromethyl)phenyl isothiocyanate was ordered from TCI chemicals. Phenylsilane was ordered from Apollo Scientific. Silver carbonate was acquired from Thermo Scientific. All commercial compounds were used as received. Dry THF, dry DCM, and dry toluene were obtained from an SPS solvent purification system (IT-Inc). Triethylamine was dried by distillation over CaH_2 and stored in the dark. All inert atmosphere reactions were carried out under an argon atmosphere with standard Schlenk-line techniques. Purification by "flash" chromatography was performed by adsorbing the samples on silica; the adsorbed samples were introduced in the solid loader and purified by means of Reveleris X2 purification system (Buchi®) using pre-packed silica cartridges Ecoflex® (irregular 50 μm silica). NMR spectra were recorded on a Bruker Avance 300 or on a Bruker Avance 400 spectrometer and calibrated to the residual solvent peak: acetone-d_6 (^{1}H: 2.05 ppm; ^{13}C: 29.84 ppm), $CDCl_3$ (^{1}H: 7.26 ppm; ^{13}C: 77.16 ppm), CD_2Cl_2 (^{1}H: 5.32 ppm; ^{13}C: 53.84 ppm), THF-d_8 (^{1}H: 3.58 ppm; ^{13}C: 67.21 ppm), and DMSO-d_6 (^{1}H: 2.50 ppm; ^{13}C: 39.52 ppm). Peaks are reported with their corresponding multiplicity (s: singlet, br s: broad singlet, d: doublet, t: triplet, q: quartet, qnt: quintet, septet, dd: doublet of doublets, dq: doublet of quartets), and integration, and respective *J* coupling constants are given in Hertz. Exact mass measurements (HRMS) were obtained on TQ R30-10 HRMS spectrometer by ESI^+ or APCI ionization, and are reported in m/z for the major signal. Solid-state FT-IR analyses were performed by drop casting a solution of the compounds onto KBr cells and by analyzing the resulting films by transmission (Figure S1).

Circular Dichroism (CD) analyses: Circular dichroism (CD) measurements were performed on a Jasco J-1500 spectrometer equipped with a Peltier thermostated cell holder and Xe laser. <u>CD analyses of Figures 1, 3, 4, S2, S4, S5, S7, S10-S14</u>: they were performed at 293 K with the following parameters: 50 $nm.min^{-1}$ sweep rate, 0.05 nm data pitch, 2.0 nm bandwidth, and between 400 and 275 nm. The solutions were placed into a cylindrical spectrosil quartz cell of 0.05 mm pathlength (Starna® 31/Q/0.05). Toluene and cell contributions at the same temperature were subtracted from the obtained signals. <u>CD analyses of Figure S8</u>: they were performed between 293 K and 393 K (heating, 1 K/min, full spectrum every 10K) with the following parameters: 50 $nm.min^{-1}$ sweep rate, 0.05 nm data pitch, 2.0 nm bandwidth, and between 400 and 275 nm. The

solutions were placed into a cylindrical spectrosil quartz cell of 0.05 mm pathlength (Starna® 31/Q/0.05). Toluene and cell contributions at the same temperature were subtracted from the obtained signals at the same temperature. CD analyses of Figure S3: they were performed at 293 K with the following parameters: 50 nm.min^{-1} sweep rate, 0.05 nm data pitch, 2.0 nm bandwidth, and between 400 and 200 nm. The solutions were placed into a cylindrical spectrosil quartz cell of 0.05 mm pathlength (Starna® 31/Q/0.05). Solvent and cell contributions at the same temperature were subtracted from the obtained signals. CD analyses of Figures 2 and S9: they were performed at 293 K with the following parameters: 50 nm.min^{-1} sweep rate, 0.05 nm data pitch, 2.0 nm bandwidth, and between 400 and 275 nm. The solutions were placed into a rectangular spectrosil quartz cell of 1.0 mm pathlength. Toluene and cell contributions at the same temperature were subtracted from the obtained signals.

All solutions were pre-heated before measurements. For all samples, the LD contribution was negligible (ΔLD < 0.005 dOD) and the shape of the CD signal was independent of the orientation of the quartz cells. In some cases, the CD intensity is reported as the molar extinction coefficient, calculated as $\Delta\varepsilon = \theta/(32982 \times$[**TATA "soldier"**]$\times$l), with θ= ellipticity (in mdeg), [**TATA "soldier"**]= concentration in **TATA "soldier"** (in mol.L^{-1}), and l= cell pathlength (in cm).

UV-Vis analyses: UV-Vis absorption spectra were extracted from CD on each of the above samples and obtained after correction of the absorption of air, solvent, and cell at 293 K.

Fourier-Transform Infrared (FT-IR) analyses: FT-IR measurements were performed on a Nicolet iS10 spectrometer. Spectra of solutions in toluene and methylcyclohexane were measured in a 0.05 cm pathlength CaF_2 cell at room temperature and were corrected for air, solvent and cell absorption. Procedure for the simulated FT-IR spectra in Figure 1: *Simulation 1 (bonded amide/free N-H (thio)urea)*: These spectra were built up by summing the FT-IR spectra of **TATA model** (2.9 mM in toluene, bonded amide) and of **thiourea** or **urea model** (2.9 mM in toluene, free N-H (thio)urea and urea functions). This yields the FT-IR signature of supramolecular stacks with pendant free thiourea or urea functions. *Simulation 2 (bonded amide/bonded N-H (thio)urea)*: These spectra were built up by summing the FT-IR spectra of **TATA model** (2.9 mM in toluene, bonded amide) and of **thiourea** or **urea model** bonded to **(*R*)-TRIP•NBu$_4$** (2.9 mM + 2.9 mM in toluene, bonded (thio)urea and urea functions). This yields the FT-IR signature of supramolecular stacks with pendant thiourea or urea functions N-H bonded to **(*R*)-TRIP**. Procedure for the simulated FT-IR spectrum in Figure S15: The spectrum was built up by summing the FT-IR spectrum obtained for *simulation 1*, i.e. the sum of the FT-IR spectra of **TATA model** (2.9 mM in toluene, bonded amide) and of **thiourea model** (2.9 mM in toluene, free N-H thiourea function), with that of **TATA P** (2.9 mM in toluene, bonded amide). This yields the FT-IR signature of supramolecular copolymers with pendant free N-H thiourea functions.

Preparation of the solutions for FT-IR and CD analyses and catalytic experiments: see below

Solutions for FT-IR and CD analyses

All solutions were gently heated prior to analyses, expect otherwise stated.

Procedure to afford the supramolecular "sergeants" between TATA *m*-U or *p*-U and TRIP•NBu4 (Figures 1, S4 and S7): A solution of **(*S*)** or **(*R*)-TRIP•NBu4** (2.9 mg, 2.9 µmol) in the desired solvent (1.0 mL) was poured into a vial containing either solid **TATA *p*-U** or solid **TATA *m*-U** (2.7 mg, 2.9 µmol).

Procedure to afford the supramolecular "sergeant" between TATA TU and TRIP•NBu4 (Figures 1, S2, S3, S4 and S7): A solution containing 0.3 equivalent, (0.87 mg, 0.87 µmol), 0.5 equivalent (1.44 mg, 1.45 µmol) or 1 equivalent of **(*S*)** or **(*R*)-TRIP•NBu4** (2.88 mg, 2.9 µmol), relatively to **TATA TU**, in toluene (1.0 mL) was poured into a vial containing solid **TATA TU** (2.79 mg, 2.9 µmol).

Procedure for the formation of the supramolecular copolymers, from pre-formed supramolecular "sergeant": Case of **TATA C8** (0.2 mM, Figures 2 and S9): A solution containing **TATA *p*-U** and **(*S*)-TRIP•NBu4** (1:1 ratio) in toluene was prepared and then an aliquot of this solution containing the desired amount of the supramolecular "sergeant' was poured into a vial containing **TATA C8** (0.14 mg, 0.2 µmol) in toluene. Toluene was added to reach a final volume of 1.0 mL. Case of **TATA TU** with the different "soldiers" (2.9 mM, Figures 3 and S10-S14): A solution containing **TATA TU** (2.8 mg, 2.9 µmol) and **(*S*)** or **(*R*)-TRIP•NBu4** (2.9 mg, 2.9 µmol) in toluene or MCH (1.0 mL) was poured into a vial containing solid **TATA C8** (2.1 mg, 2.9 µmol), **TATA P** (3.3 mg, 2.9 µmol), **TATA PCF3** (4.1 mg, 2.9 µmol) or T**ATA *N* C20** (3.40 mg, 2.9 µmol).

Procedure for the in-situ induction of chirality into supramolecular copolymers (Figures 4 and S15): 10 µL of a solution of **(*S*)-TRIP•NBu4** (5.8 mg, 5.8 µmol) in toluene (0.2 mL) were incrementally added to a solution containing **TATA P** (3.3 mg, 2.9 µmol) and **TATA TU** (2.8 mg, 2.9 µmol) in toluene (1.0 mL) until 100 µL has been added (2.9 mg, 2.9 µmol). The solution was stirred for three minutes but not heated prior to analysis.

Catalytic experiments

General procedure: Experiments with TATA mixtures were performed similarly to our previous study with BTA mixtures.[3] A pre-catalytic mixture composed of the ligand and the copper salt was prepared as follows: oven-dried test tubes were loaded with a stock solution prepared by mixing **TATA ligand** and [$Cu(OAc)_2 \cdot H_2O$], divided in order to get **TATA ligand** (1.74 µmol, 6.0 mol%) and [$Cu(OAc)_2 \cdot H_2O$] (0.08 mg, 0.44 µmol, 1.5 mol%) in dry THF (500 µL) in each tube. The solvent was removed under vacuum and the tubes were kept under vacuum (10^{-3} mbar) for 1 hour. Solid 4-nitroacetophenone (4.8 mg, 29.0 µmol, 100 mol%) and a solution of **TATA *m*-U**, **TATA *p*-U** or **TATA TU** (1.74 µmol, 6.0 mol%) with **(*S*)** or **(*R*)-TRIP•NBu$_4$** (1.74 µmol, 6.0 mol%) in toluene (600 µL) were added to the tube. The mixture was then briefly heated to the solvent boiling point (≈ 383 K). After cooling to room temperature, the stirring bar was introduced and the mixture was stirred for 15 min. Phenylsilane (8 µL, 58 µmol, 200 mol%) was then added and the reaction mixture was stirred vigorously for 17 h at room temperature. Typical work-up: Aqueous solution of HCl (10 wt%, 1000 µL) was added and the mixtures were stirred for 30 min (until the solution became transparent). Then, the products were extracted with Et_2O (500 µL) and AcOEt (500 µL) and the organic phase was passed through a small silica plug. The solid was washed with AcOEt. The solvents were evaporated and the crude material was analyzed by NMR and by chiral GC. Conversion and enantiomeric excess (*ee*) were determined by chiral GC analysis. *Ee* values are indicated as positive and negative when (*S*)-4-nitroacetophenol and (*R*)-4-nitroacetophenol are the major enantiomers, respectively. Chiral GC analyses:[4] The optical purity was determined by GC analysis: Chiral Cyclosil-B column, 30 m × 250 µm x 0.25 µm, inlet pressure= 12.6 psi. Injection temperature= 250°C; detector temperature= 300°C; column temperature= 135°C. Retention time: 18 min (4-nitroacetophenone), 46 min ((*R*)-4-nitroacetophenol), 48 min ((*S*)-4-nitroacetophenol).

Catalytic tests with the conventional "sergeants" (*R*)-TATA* and (*S*)-TATA*: Oven-dried test tubes were loaded with a stock solution prepared by mixing **TATA PCF3** and [$Cu(OAc)_2 \cdot H_2O$], divided in order to get **TATA PCF3** (2.5 mg, 1.74 µmol, 6.0 mol%) and $Cu(OAc)_2 \cdot H_2O$] (0.08 mg, 0.44 µmol, 1.5 mol%) in dry THF (500 µL) in each tube. The solvent was **removed** under vacuum and the tubes were kept under vacuum (10^{-3} mbar) for 1 hour. Solid 4-nitroacetophenone (4.8 mg, 29.0 µmol, 100 mol%), **(*R*)-TATA*** or **(*S*)-TATA*** (1.1 mg, 1.74 µmol, 6.0 mol%) and toluene (600 µL) were added to the pre-catalyst solid and the mixture was gently heated to the solvent boiling point (≈ 383 K) and cooled down to room temperature. The stirring bar was introduced and the mixture was stirred for 15 min. Phenylsilane (8 µL, 58 µmol, 200 mol%) was then added and the reaction mixture was stirred vigorously for 17 h at room temperature. Work-up was similar to the one reported in the general procedure.

Representative chiral GC analyses are given in Figures S16-S17.

Synthetic procedures

Synthesis of intermediates

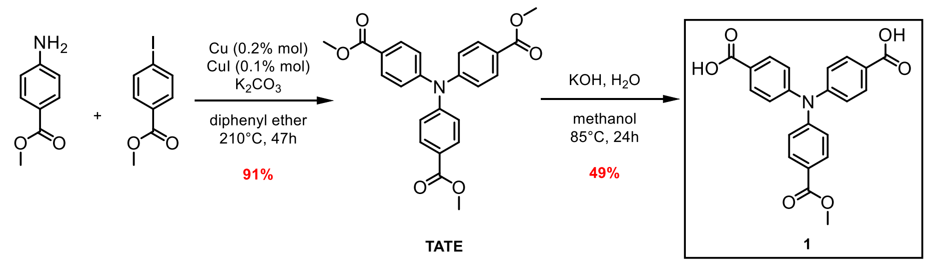

4,4',4''-tris-[*p*-methyl ester]-triarylamine (TATE): Inspired from the procedure of Swart et al.,[5] a 250 mL Schlenk flask was filled with methyl 4-amino-benzoate (5.00 g, 35 mmol, 1 equiv.), methyl 4-iodo-benzoate (26.0 g, 105 mmol, 3 equiv.), copper powder (0.42 g, 7 mmol), copper iodide (0.67 g, 3.5 mmol, 0.1 equiv.), and potassium carbonate (9.32 g, 70 mmol, 2 equiv.) under inert atmosphere. The materials were dried in vacuo for 24 hours then diphenyl ether (43 mL) is added in the Schlenk flask and the reaction mixture was heated to 210°C for 24 hours. After cooling down to room temperature, the reactive medium was filtered through silica and the residue was washed with 300 mL of ethyl acetate and 100 mL of dichloromethane. The organic layers were combined then evaporated and diphenyl ether was removed by distillation under pressure. The crude material was purified by column chromatography (SiO_2, PE/ EtOAc, 70/30) affording **TATE** (12.2 g, 91%) as a colourless solid. ^{1}H NMR (400 MHz, $CDCl_3$): δ (ppm)= 7.96 (apparent d, *J*= 8.9 Hz, 6H), 7.12 (apparent d, *J*= 8.9 Hz, 6 H), 3.91 (s, 9H). The NMR spectrum matches the one reported in the literature.[6]

4,4'-bis-[*p*-carboxylic acid]-4''-[*p*-methyl ester]-triarylamine (1): TATE (1.88 g, 4.45 mmol) was dissolved into 100 mL of methanol and potassium hydroxide (750 mg, 13.3 mmol, 3 equiv.) was added. The reactive mixture was heated to 85°C for 20 hours. After removal of most of the solvent, the medium was quenched with 1N of aqueous HCl to pH 1 and washed with diethyl ether three times. The combined organic layers were dried over $MgSO_4$ and evaporated. The crude material was purified by "flash" column chromatography (SiO_2, DCM/EtOAc, 100/0 to 78/22) affording compound **1** (857 mg, 49%) as a colourless solid. ^{1}H NMR (400 MHz, acetone-d_6): δ (ppm)= 7.97 (apparent d, *J*= 8.9 Hz, 4H), 7.94 (apparent d, *J*= 8.9 Hz, 2H), 7.18 (apparent d, *J*= 8.7 Hz, 6H), 3.83 (s, 3H) ; ^{13}C{^{1}H} NMR (101 MHz, acetone-d_6): δ (ppm)= 167.13, 166.63, 151.46, 151.35, 132.22, 131.90, 126.70, 126.25, 124.82, 124.69, 52.23 ; HMRS (ESI): m/z calculated for $C_{22}H_{16}NO_6$ [M-H]$^-$: 390.0983, found: 390.0985 (0.5 ppm).

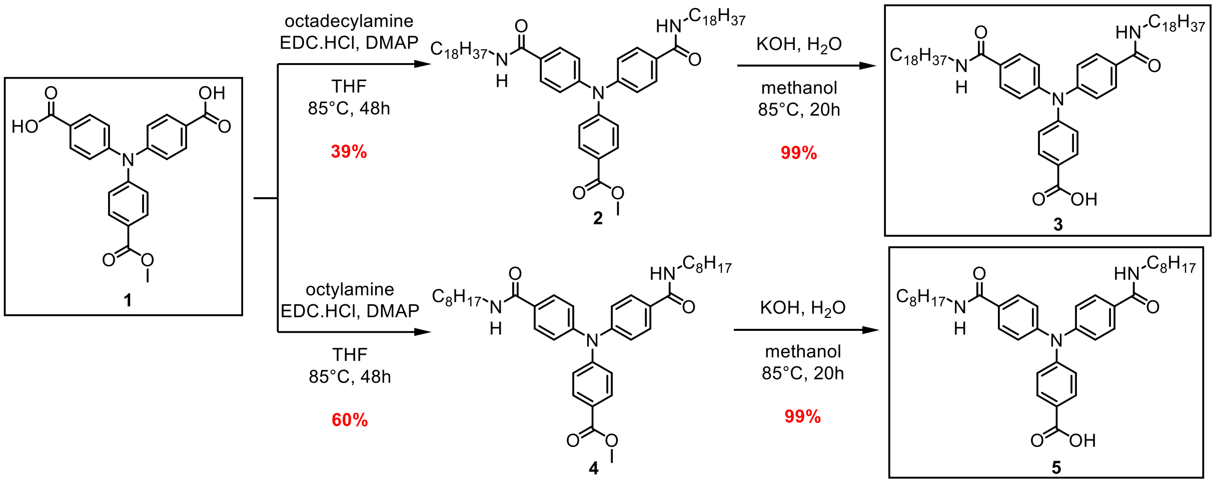

4,4’-bis-[*p*-octadecylamide]-4’’-[*p*-methyl ester]-triarylamine (2): Compound **1** (856 mg, 2.19 mmol, 1 equiv.), octadecylamine (1.77 g, 6.57 mmol, 3 equiv.), EDC•HCl (1.43 g, 7.44 mmol, 3.4 equiv.), and DMAP (0.91 g, 7.44 mmol, 3.4 equiv.) were dissolved into THF (82 mL) under argon. The corresponding mixture was heated to 85°C for two days. The mixture was cooled down to room temperature and the solvent was evaporated. 70 mL of DCM was then added and the organic layer was washed successively with aqueous HCl (0.1 M), with aqueous $NaHCO_3$ (0.1M) and brine. The organic phase was dried over $MgSO_4$, evaporated under vacuum and the crude material was purified by “flash” column chromatography (SiO_2, DCM/EtOAc, 100/0 to 85/15) affording compound **2** (766 mg, 39%) as a colourless solid. ^1H NMR (400 MHz, $CDCl_3$): δ (ppm)= 7.90 (apparent d, *J*= 8.7 Hz, 2H), 7.68 (apparent d, *J*= 8.7 Hz, 4H), 7.10 (apparent d, *J*= 8.7 Hz, 4H), 7.06 (apparent d, *J*= 8.7 Hz, 2H), 6.18 (t, *J*= 5.7 Hz, 2H), 3.88 (s, 3H), 3.43 (q, *J*= 7.0 Hz, 4H), 1.61 (qnt, *J*= 7.0 Hz, 4H), 1.39-1.22 (m, 60H), 0.88 (t, *J*= 6.8 Hz, 6H) ; $^{13}C\{^1H\}$ NMR (101 MHz, $CDCl_3$): δ (ppm)= 166.80, 166.63, 150.79, 149.16, 131.24, 130.47, 128.57, 124.81, 124.47, 122.91, 52.15, 40.29, 32.05, 29.86, 29.83, 29.79, 29.74, 29.70, 29.49, 27.16, 22.82, 14.25 ; HMRS (ESI): m/z calculated for $C_{58}H_{91}N_3O_4H$ $[M+H]^+$: 894.7082, found: 894.7086 (0.4 ppm).

4,4’-bis-[*p*-octadecylamide]-4’’-[*p*-carboxylic acid]-triarylamine (3): To a solution of compound **2** (2.31 g, 2.58 mmol, 1 equiv.) in a mixture of 400 mL of methanol and 20 mL of water was added potassium hydroxide (2.17 g, 38.7 mmol, 15 equiv.). The reaction mixture was heated to 85°C for 20 h. After cooling to room temperature, 250 mL of water then 50 mL of aqueous HCl (1M) were slowly poured to the reaction mixture. The formed precipitate was isolated by filtration over a glass frit and washed with water. The colorless solid was dried under vacuum overnight over P_2O_5 yielding pure compound **4** (2.26 g, 99%) as a colourless solid. ^1H NMR (400 MHz, THF-d_8): δ (ppm)= 7.89 (apparent d, *J*= 8.6 Hz, 2H), 7.79 (apparent d, *J*= 8.6 Hz, 4H), 7.54 (t, *J*= 5.8 Hz, 2H), 7.11 (apparent d, *J*= 8.6 Hz, 4H), 7.07 (apparent d, *J*= 8.7 Hz, 2H), 3.34 (q, *J*= 6.7 Hz, 4H), 1.57 (qnt, *J*= 7.2 Hz, 4H), 1.43-1.26 (m, 60H), 0.89 (t, *J*= 6.8 Hz, 6H) ; $^{13}C\{^1H\}$ NMR (101 MHz, THF-d_8): δ (ppm)= 166.85, 165.91, 151.62, 149.64, 131.76, 131.74, 129.29, 125.96, 124.71, 123.14, 40.30, 32.68, 30.73, 30.46, 30.42, 30.26, 30.12, 27.86, 23.37, 14.25 ; HMRS (ESI): m/z calculated for $C_{57}H_{89}N_3O_4H$ $[M+H]^+$: 880.6926, found: 880.6926 (0.4 ppm).

4,4'-bis-[*p*-octylamide]-4''-[*p*-methyl ester]-triarylamine (4): Compound **1** (1.30 g, 3.32 mmol, 1 equiv.), octylamine (1.65 mL, 9.96 mmol, 3 equiv.), EDC•HCl (2.17 g, 6,3 mmol, 3.4 equiv.) and DMAP (1.38 g, 6.3 mmol, 3.4 equiv.) were dissolved into THF (75 mL) under argon. The corresponding mixture was heated to 85°C for two days. The mixture was cooled down to room temperature and the solvent was evaporated. 70 mL of DCM was added and the organic layer was washed successfully with aqueous HCl (0.1 M), with aqueous $NaHCO_3$ (0.1M) and brine. The organic phase was dried over $MgSO_4$ and after concentration in vacuo, the crude material was purified by "flash" column chromatography (SiO_2, DCM/EtOAc, 100/0 to 85/15) affording compound **4** (1.22 g, 60%) as a colourless solid. ^1H NMR (400 MHz, $CDCl_3$): δ (ppm)= 7.90 (apparent d, *J*= 8.8 Hz, 2H), 7.68 (apparent d, *J*= 8.7 Hz, 4H), 7.10 (apparent d, *J*= 8.7 Hz, 4H), 7.06 (apparent d, *J*= 8.9 Hz, 2H), 6.18 (t, *J*= 5.8 Hz, 2H), 3.88 (s, 3H), 3.43 (q, *J*= 6.5 Hz, 4H), 1.60 (qnt, *J*= 7.3 Hz, 4H), 1.40-1.21 (m, 20H), 0.87 (t, *J*= 7.1 Hz, 6H) ; $^{13}C\{^1H\}$ NMR (101 MHz, $CDCl_3$) δ (ppm)= 166.81, 166.65, 150.80, 149.16, 131.24, 130.47, 128.57, 124.81, 124.48, 122.92, 52.16, 40.28, 31.92, 29.85, 29.42, 29.34, 27.15, 22.76, 14.22 ; HMRS (ESI): m/z calculated for $C_{38}H_{51}N_3O_4H$ $[M+H]^+$: 614.3952, found: 614.3951 (0.2 ppm).

4,4'-bis-[*p*-octylamide]-4''-[*p*-carboxylic acid]-triarylamine (5): To a solution of compound **4** (1.22 g, 1.98 mmol, 1 equiv.) in a mixture of 180 mL of methanol and 9 mL of water was added potassium hydroxide (1.67 g, 29.8 mmol, 15 equiv.). The reaction mixture was heated to 85°C for 20 h. After cooling to room temperature, 250 mL of water then 50 mL of aqueous HCl (1M) were slowly poured to the reaction mixture. The formed precipitate was isolated by filtration over a glass frit and washed with water. The colorless solid was dried under vacuum overnight over P_2O_5 yielding pure compound **5** (1.18 g, 99%) as a colourless solid. ^1H NMR (400 MHz, DMSO-d_6): δ (ppm)= 12.73 (s, 1H), 8.39 (t, *J*= 5.7 Hz, 2H), 7.86 (apparent d, *J*= 8.8 Hz, 2H), 7.83 (apparent d, *J*= 8.7 Hz, 4H), 7.11 (apparent d, *J*= 8.7 Hz, 4H), 7.05 (apparent d, *J*= 8.8 Hz, 2H), 3.23 (q, *J*= 6.6 Hz, 4H), 1.49 (qnt, *J*= 6.9 Hz, 4H), 1.35-1.21 (m, 20H), 0.84 (t, *J*= 6.9 Hz, 6H) ; $^{13}C\{^1H\}$ NMR (101 MHz, DMSO-d_6): δ (ppm)= 166.80, 165.29, 150.29, 148.32, 131.02, 130.21, 128.91, 124.81, 124.00, 122.17, 39.18, 31.29, 29.19, 28.80, 28.71, 26.53, 22.12, 13.96 ; HMRS (ESI): m/z calculated for $C_{37}H_{49}N_3O_4H$ $[M+H]^+$: 600.3796, found: 600.3793 (0.5 ppm).

Synthesis of non-functionalized TATA monomers

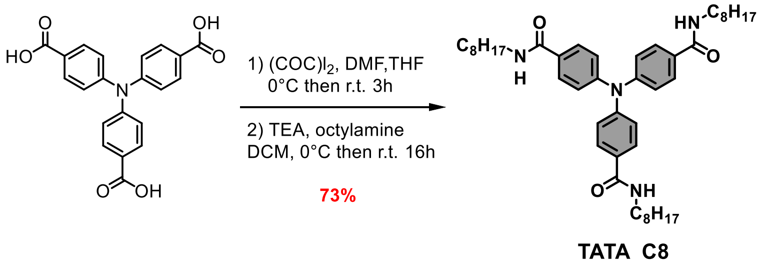

TATA C8: In an oven-dried Schlenk flask under argon atmosphere, 4,4',4''-tris-[*p*-carboxylic acid]-triarylamine[6] (0.25 g, 0.62 mmol, 1 equiv.) was dissolved in THF (11 mL). A few droplets of DMF was added and the mixture was cooled to 0°C with an ice-bath. Oxalyl chloride (0.21 mL, 2.38 mmol, 3.6 equiv.) was added dropwise and the stirred mixture was allowed to reach room

temperature. After 3 h, the solvent and the excess of oxalyl chloride were evaporated. The acyl chloride intermediate was dissolved in DCM (22 mL), cooled to 0°C with an ice-bath, and octylamine (0.36 mL, 2.18 mmol, 3.3 equiv.) was added. Triethylamine (0.65 mL, 4.64 mmol, 7 equiv.) was added dropwise. The reaction mixture was allowed to reach room temperature and stirred for additional 16 h. 20 mL of brine was added and the reaction mixture was extracted with DCM (3x40 mL). The organic layers were combined, dried over anhydrous $MgSO_4$, then filtered through a plug of silica and evaporated. The crude material was purified by "flash" column chromatography (SiO2, DCM/EtOAc, 80/20) yielding **TATA C8** (0.34 g, 73%) as a colourless solid. ^{1}H NMR (400 MHz, $CDCl_3$): δ (ppm)= 7.66 (apparent d, *J*= 8.3 Hz, 6H, CH_{arom}), 7.06 (apparent d, *J*= 8.3 Hz, 6H, CH_{arom}), 6.23 (t, *J*= 5.7 Hz, 3H, NH), 3.43 (q, *J*= 6.7 Hz, 6H, NHCH_2), 1.60 (qnt, *J*= 7.1 Hz, 6H, $NHCH_2CH_2$), 1.40-1.25 (m, 30H, CH_2), 0.87 (t, *J*= 6.6 Hz, 9H, CH_3) ; ^{13}C{^{1}H} NMR (101 MHz, $CDCl_3$): δ (ppm)= 166.91, 149.34, 130.05, 128.54, 123.98, 40.29, 31.93, 29.85, 29.44, 29.36, 27.16, 22.77, 14.22 ; HMRS (ESI): m/z calculated for $C_{45}H_{66}N_4O_3H$ $[M+H]^+$: 711.5208, found: 711.5205 (0.4 ppm) ; FT-IR: υ (cm^{-1})= 3293, 3083, 3038, 2954, 2925, 2853, 2360, 2343, 1631, 1601, 1544, 1499, 1466, 1457, 1437, 1375, 1364, 1314, 1296, 1279, 1181, 1148, 1107.

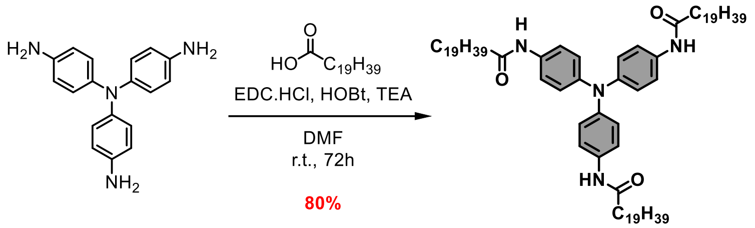

TATA *N* C20

TATA *N* C20: N-eicosanoic acid (2.44 g, 7.81 mmol, 4.5 equiv.) was dissolved in 120 mL of anhydrous DMF and EDC•HCl (1.50 g, 7.81 mmol, 4.5 equiv.) was added to the solution at 0°C under argon, followed by the dropwise addition of triethylamine (0.79 g, 7.81 mmol, 4.5 equiv.). The reaction mixture was stirred for 10 min at 0°C. HOBt (1.06 g, 7.81 mmol, 4.5 equiv.) was then added in one portion and the reaction was stirred for 10 min at 0°C. Finally, 4,4',4"-triaminotriphenylamine (504 mg, 1.74 mmol, 1 equiv.) was added at 0°C, and the red-colored mixture was heated to 60°C for 43 h. The reaction mixture was cooled down to room temperature and quenched with brine (100 mL). The aqueous phase was extracted with diethyl ether (3 x 100 mL). The organic layers were combined and washed successively with brine, with aqueous HCl (0.1 M) and with aqueous $NaHCO_3$ (up to pH=8), dried over $MgSO_4$ and evaporated under vacuum. The crude material was recrystallized from ethanol and from ethyl acetate and dried over vacuum to give **TATA *N* C20** (1.63 g, 80% yield) as a colourless solid. ^{1}H NMR (400 MHz, THF-d_8): δ (ppm)= 8.82 (s, 3H, NH), 7.48 (apparent d, *J*= 8.6 Hz, 6H, CH_{arom}), 6.90 (apparent d, *J*= 8.0 Hz, 6H, CH_{arom}), 2.26 (t, *J*= 7.4 Hz, 6H, COCH_2), 1.66 (qnt, *J*= 7.0 Hz, 6H, $COCH_2CH_2$), 1.40-1.29 (m, 96H, CH_2), 0.89 (t, *J*= 7.0 Hz, 9H, CH_3) ; ^{13}C{^{1}H} NMR (101 MHz, THF-d_8, 300 K): δ (ppm)= 170.73, 144.03, 135.59, 124.46, 120.52, 37.50, 32.68, 30.45 (m), 30.40, 30.36, 30.30, 30.14, 30.11, 26.30, 23.36, 14.24 ; HRMS (ESI, m/z): calculated for $C_{78}H_{132}N_4O_3Na$, $[M+Na]^+$: 1196.0192,

found: 1196.0194 ; FT–IR (cm^{-1}): 3293, 2953, 2918, 2849, 1655, 1600, 1522, 1508, 1466, 1406, 1377, 1315, 1267, 1247.

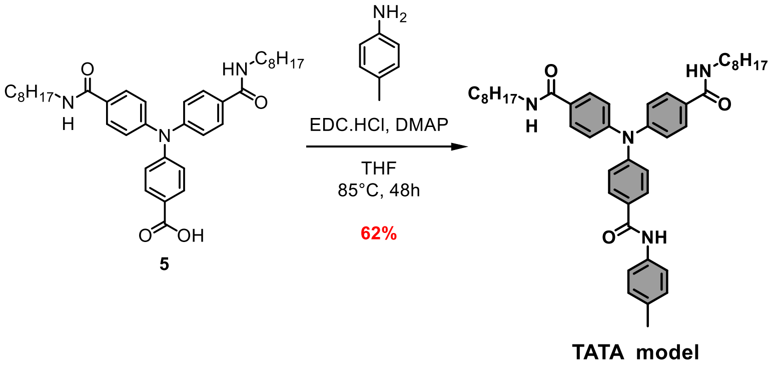

TATA model: In an oven-dried 100 mL Schlenk flask under argon atmosphere, compound **5** (100 mg, 0.17 mmol, 1 equiv.), EDC•HCl (54 mg, 0.28 mmol, 1.7 equiv.) and DMAP (35 mg, 0.28 mmol, 1.7 equiv.) were solubilized in THF (20 mL). Then *p*-toluidine (73 mg, 0.68 mmol, 4 equiv.) was added and the reaction mixture was stirred to 85 °C for 48 hours. The reaction mixture was cooled down to room temperature and the solvent was removed under vacuum. The crude material was purified by "flash" column chromatography (SiO_2, DCM/EtOAc, 90/10) yielding **TATA model** (71 mg, 62%) as a yellow solid. ^{1}H NMR (400 MHz, DMSO-d_6): δ (ppm)= 10.08 (s, 1H, NH), 8.37 (t, *J*= 5.8 Hz, 2H, NH), 7.92 (apparent d, *J*= 8.4 Hz, 2H, CH_{arom}), 7.83 (apparent d, *J*= 8.4 Hz, 4H, CH_{arom}), 7.64 (apparent d, *J*= 7.9 Hz, 2H, CH_{arom}), 7.17-7.06 (m, 8H, CH_{arom}), 3.24 (q, *J*= 6.7 Hz, 4H, $NHCH_2$), 2.28 (s, 3H, $ArCH_3$), 1.51 (qnt, 4H, $NHCH_2CH_2$), 1.37-1.20 (m, 20H, CH_2), 0.86 (t, *J*= 7.0 Hz, 6H, CH_3) ; $^{13}C\{^{1}H\}$ NMR (101 MHz, DMSO-d_6): δ (ppm)= 165.32, 164.55, 149.07, 148.52, 136.71, 132.47, 129.84, 129.58, 129.34, 128.96, 128.85, 123.50, 122.99, 120.38, 39.16, 31.26, 29.17, 28.77, 28.68, 26.51, 22.10, 20.49, 13.96 ; HMRS (ESI): *m/z* calculated for $C_{44}H_{56}N_4O_3Na$ $[M+Na]^+$: 711.4245, found: 711.4247 (0.3 ppm) ; FTIR : υ (cm^{-1}) = 3308, 3106, 3045, 2954, 2928, 2871, 2856, 2360, 2341, 1704, 1635, 1598, 1556, 1515, 1498, 1473, 1442, 1404, 1389, 1309, 1278, 1228, 1180, 1135, 1112, 1097, 1048, 1016.

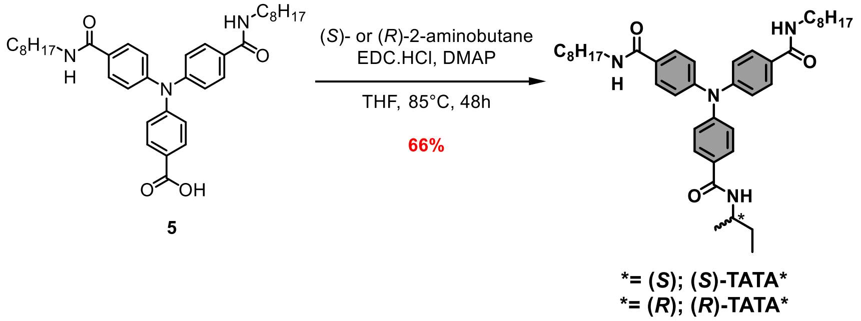

(*S*)-TATA*: In an oven-dried 100 mL Schlenk flask under argon atmosphere, compound **5** (500 mg, 0.83 mmol, 1 equiv.), EDC•HCl (271 mg, 1.42 mmol, 1.7 equiv.) and DMAP (173 mg, 1.42 mmol, 1.7 equiv.) were solubilized in THF (20 mL). Then (*S*)-2-aminobutane (67 µL, 1.25 mmol, 1.5 equiv.) was added and the reaction mixture was stirred to 85 °C for 48 hours. The reaction

mixture was cooled down to room temperature and the solvent was removed under vacuum. The crude material was purified by "flash" column chromatography (SiO_2, DCM/EtOAc, 70/30) yielding pure **(*S*)-TATA*** (360 mg, 66%) as a colourless solid. ^1H NMR (400 MHz, $CDCl_3$): δ (ppm)= 7.63 (apparent d, *J*= 8.6 Hz, 6H, CH_{arom}), 6.97 (apparent d, *J*= 8.3 Hz, 6H, CH_{arom}), 6.66 (t, *J*= 5.7 Hz, 2H, NH), 6.32 (d, *J*= 8.4 Hz, 1H, NH), 4.09 (septet, *J*= 6.5 Hz, 1H, CH), 3.40 (q, *J*= 6.7 Hz, 4H, $NHCH_2$), 1.65-1.50 (m, 6H, $NHCH_2CH_2$ + $CHCH_2CH_3$), 1.38-1.24 (m, 20H, CH_2), 1.20 (d, *J*= 6.6 Hz, 3H, $CHCH_3$), 0.93 (t, *J*= 7.4 Hz, 3H, $CHCH_2CH_3$), 0.86 (t, *J*= 6.9 Hz, 6H, $CH_2CH_2CH_3$) ; 13C{^1H} NMR (101 MHz, $CDCl_3$): δ (ppm)= 166.93, 166.37, 149.18, 130.14, 129.96, 128.63, 128.60, 123.85, 123.77, 47.29, 40.29, 31.89, 29.83, 29.81, 29.42, 29.33, 27.16, 22.73, 20.59, 14.19, 10.65 ; HMRS (ESI): m/z calculated for $C_{41}H_{58}N_4O_3Na$ $[M+Na]^+$: 677.4401, found: 677.4402 (0.1 ppm) ; FT-IR: υ (cm^{-1}) = 3304, 3069, 3040, 2958, 2926, 2870, 2855, 2334, 1633, 1599, 1548, 1499, 1454, 1377, 1349, 1314, 1281, 1185,1153, 1102.

(*R*)-TATA* was synthesized following the same procedure using (*R*)-2-aminobutane; analytical data are identical to **(*S*)-TATA***.

Synthesis of phosphine-functionalized TATA monomers

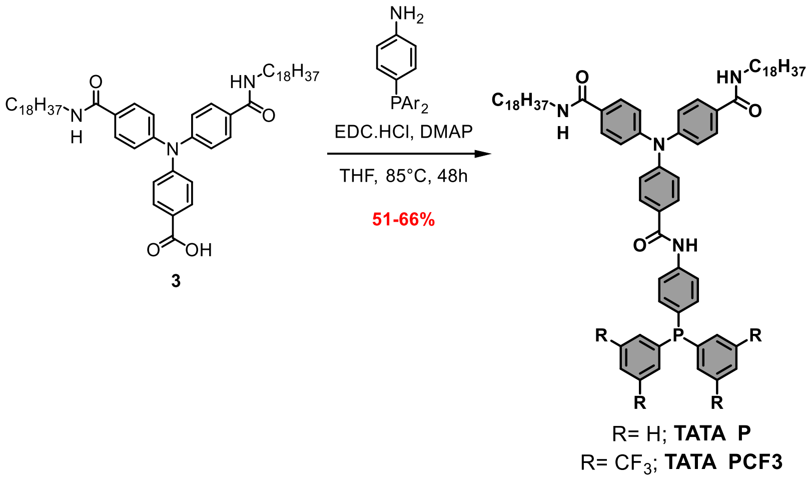

TATA P: In an oven-dried 100 mL Schlenk flask under argon atmosphere, compound **3** (500 mg, 0.57 mmol, 1.0 equiv.), EDC•HCl (196 mg, 1.02 mmol, 1.8 equiv.) and DMAP (125 mg, 1.02 mmol, 1.8 equiv.) were solubilized in THF (17 mL). Then 4-(bis(phenylphosphino))aniline[7] (189 mg, 0.68 mmol, 1.2 equiv.) in THF (3 mL) was added and the reaction mixture was stirred to 85 °C for 36 hours. The reaction mixture was cooled down to room temperature and the solvent was removed under vacuum. The crude material was purified by "flash" column chromatography (SiO_2, DCM/EtOAc, 80/20) yielding pure **TATA P** (426 mg, 66%) as a pale-yellow powder. ^1H NMR (400 MHz, $CDCl_3$): δ (ppm)= 8.12 (s, 1H, NH), 7.77 (apparent d, *J*= 8.7 Hz, 2H, CH_{arom}), 7.66 (apparent d, *J*= 8.3 Hz, 6H, CH_{arom}), 7.34-7.29 (m, 12H, PPh_2 + CH_{arom}), 7.07 (apparent d, *J*= 8.6 Hz, 6H, CH_{arom}), 6.16 (t, *J*= 5.7 Hz, 2H, NH), 3.43 (q, *J*= 6.7 Hz, 4H, $NHCH_2$), 1.67-1.55 (m, 4H, $NHCH_2CH_2$), 1.32-1.25 (m, 60H, CH_2), 0.88 (t, *J*= 6.7 Hz, 6H, CH_3) ; $^{13}C\{^1H\}$ NMR (101 MHz, $CDCl_3$): δ (ppm)= 166.94, 165.44, 149.68, 149.12, 139.15, 137.43 (d, *J*= 10.5 Hz), 134.83 (d, *J*=

20.5 Hz), 133.71 (d, *J*= 19.4 Hz), 130.23, 129.75, 129.03, 128.81, 128.66, 128.59, 124.09, 123.62, 120.34 (br s), 40.36, 32.05, 29.84 (br s), 29.73, 29.49, 27.19, 22.82, 14.26 ; $^{31}P\{^{1}H\}$ NMR (162 MHz, $CDCl_3$): δ (ppm)= −6.36 ; HRMS (APCI): m/z calculated for $C_{75}H_{103}N_4O_3PH$ $[M+H]^+$: 1139.7841, found: 1139.7850 (0.8 ppm) ; FT-IR: υ (cm^{-1})= 3300, 3062, 2922, 2851, 2360, 2342, 1632, 1597, 1547, 1525, 1500, 1467, 1434, 1394, 1372, 1317, 1281, 1182.

TATA PCF3: In an oven-dried 100 mL Schlenk flask under argon atmosphere, compound **3** (500 mg, 0.57 mmol, 1.0 equiv.), EDC•HCl (196 mg, 1.02 mmol, 1.8 equiv.) and DMAP (125 mg, 1.02 mmol, 1.8 equiv.) were solubilized in THF (17 mL). Then 4-(bis(3,5-bis(trifluoro-methyl)phenyl)phosphino)aniline[8] (374 mg, 0.68 mmol, 1.2 equiv.) in THF (3 mL) was added and the reaction mixture was stirred to 85 °C for 36 hours. The reaction mixture was cooled down to room temperature and the solvent was removed under vacuum. The crude material was purified by "flash" column chromatography (SiO_2, DCM/EtOAc, 80/20) yielding pure **TATA PCF3** (408 mg, 51%) as a pale-yellow solid. ^{1}H NMR (400 MHz, $CDCl_3$): δ (ppm)= 8.60 (apparent d, *J*= 7.2 Hz, 1H, NH), 7.89-7.87 (m, 4H, CH_{arom}), 7.79 (apparent d, *J*= 8.7 Hz, 2H, CH_{arom}), 7.72 (d, *J*= 6.5 Hz, 4H, PAr_2), 7.65 (apparent d, *J*= 8.7 Hz, 4H, CH_{arom}), 7.36 (t, *J*= 8.4 Hz, 2H, PAr_2), 7.06-7.03 (m, 6H, CH_{arom}), 6.29-6.25 (m, 2H, NH), 3.42 (q, *J*= 6.7 Hz, 4H, $NHCH_2$), 1.60 (qnt, *J*= 7.2 Hz, 4H, $NHCH_2CH_2$), 1.35-1.25 (m, 60H, CH_2), 0.87 (t, *J*= 6.7 Hz, 6H, CH_3) ; $^{13}C\{^{1}H\}$ NMR (101 MHz, $CDCl_3$): δ (ppm)= 166.91, 165.46, 149.95, 149.14, 141.09, 140.15 (d, *J*= 17.9 Hz), 135.37 (d, *J*= 22.5 Hz), 133.03 (dq, *J*= 23.6 Hz, 3.8 Hz), 132.36 (dd, *J*= 33.5, 6.2 Hz), 131.85 (br s), 130.32, 129.38, 129.07, 128.67, 127.20 (br s), 124.21, 123.60, 123.40 (m), 123.10 (q, *J*= 273.2 Hz, CF_3), 120.96 (d, *J*= 8.6 Hz), 40.38, 32.06, 29.84 (br s), 29.80, 29.76, 29.72, 29.50, 27.18, 22.83, 14.26 ; $^{31}P\{^{1}H\}$ NMR (162 MHz, $CDCl_3$): δ (ppm)= −4.85 ; $^{19}F\{^{1}H\}$ NMR (377 MHz, $CDCl_3$): δ (ppm)= −63.94 ; HMRS (APCI): m/z calculated for $C_{79}H_{99}F_{12}N_4O_3PH$ $[M+H]^+$: 1411.7336, found: 1411.7359 (1.6 ppm) ; FT-IR: υ (cm^{-1})= 3292, 3182, 3085, 3040, 2918, 2849, 1633, 1600, 1543, 1501, 1468, 1438, 1396, 1353, 1318, 1282, 1278, 1184, 1139, 1108, 1096, 1015.

Synthesis of urea-functionalized TATA monomers

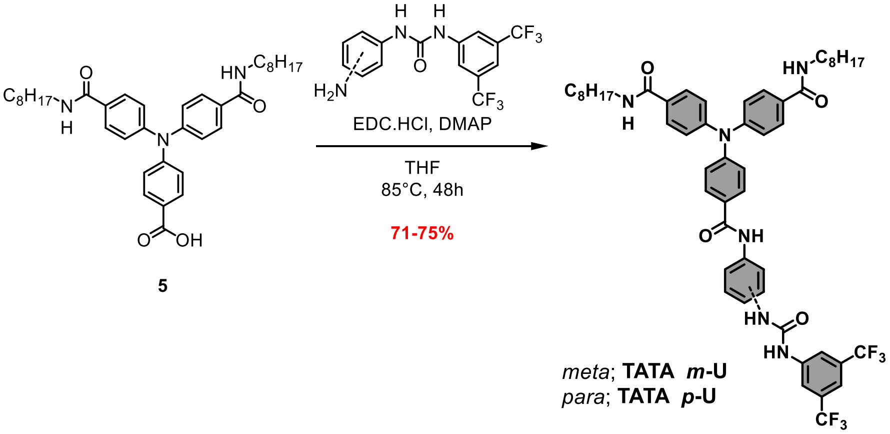

TATA *m*-U: In an oven-dried 100 mL Schlenk flask under argon atmosphere, compound **5** (500 mg, 0.83 mmol, 1 equiv.), EDC•HCl (320 mg, 1.67 mmol, 2 equiv.) and DMAP (205 mg, 1.67

mmol, 2 equiv.) were solubilized in THF (20 mL). Then *N*-(3-aminophenyl)-*N'*-[3,5-bis(trifluoromethyl)phenyl]urea[9] (333 mg, 0.92 mmol, 1.1 equiv.) was added and the reaction mixture was stirred to 85 °C for 48 hours. The reaction mixture was cooled down to room temperature and the solvent was removed under vacuum. The crude material was purified by "flash" column chromatography (SiO_2, DCM/EtOAc, 70/30 to 50/50) yielding pure **TATA *m*-U** (600 mg, 75% yield) as a colorless solid. ^1H NMR (400 MHz, DMSO-d_6): δ (ppm)= 10.20 (s, 1H, NH), 9.33 (s, 1H, NH), 9.07 (s, 1H, NH), 8.38 (t, *J*= 5.7 Hz, 2H, NH), 8.15 (s, 2H, Ar_{CF3}), 8.05 (s, 1H, CH_{arom}), 7.95 (apparent d, *J*= 8.3 Hz, 2H, CH_{arom}) 7.84 (apparent d, *J*= 8.2 Hz, 4H, CH_{arom}), 7.63 (s, 1H, Ar_{CF3}), 7.45 (d, *J*= 7.8 Hz, 1H, CH_{arom}), 7.27-7.20 (m, 2H, CH_{arom}), 7.14-7.11 (m, 6H, CH_{arom}), 3.24 (q, *J*= 6.7 Hz, 4H, $NHCH_2$), 1.52-1.47 (m, 4H, $NHCH_2CH_2$), 1.31-1.23 (m, 20H, CH_2), 0.85 (t, *J*= 6.9 Hz, 6H, CH_3) ; $^{13}C\{^1H\}$ NMR (101 MHz, DMSO-d_6): δ (ppm)= 165.31, 164.80, 152.33, 149.17, 148.51, 141.87, 139.77, 139.24, 130.72 (q, *J*= 32.6 Hz), 129.88, 129.45, 128.86, 123.33 (q, *J*= 272.8 Hz, CF_3), 123.54, 122.89, 117.93 (br s), 114.62-114.03 (m), 110.80, 39.17, 31.27, 29.19, 28.78, 28.69, 26.52, 22.10, 13.94 ; $^{19}F\{^1H\}$ NMR (377 MHz, DMSO-d_6): δ (ppm)= −62.62 ; HMRS (ESI): m/z calculated for $C_{52}H_{58}F_6N_6O_4Na$ $[M+Na]^+$: 967.4316, found: 967.4311 (0.5 ppm) ; FT-IR : υ (cm^{-1})= 3312, 3100, 3040, 2955, 2927, 2870, 2856, 2362, 2333, 1700, 1633, 1598, 1548, 1499, 1473, 1443, 1419, 1388, 1317, 1278, 1212, 1177, 1135, 1111, 1045, 1014.

TATA *p*-U: In an oven-dried 100 mL Schlenk flask under argon atmosphere, compound **5** (500 mg, 0.83 mmol, 1 equiv.), EDC•HCl (320 mg, 1.67 mmol, 2 equiv.) and DMAP (205 mg, 1.67 mmol, 2 equiv.) were solubilized in THF (20 mL). Then *N*-(4-aminophenyl)-*N'*-[3,5-bis(trifluoromethyl)phenyl]urea[9] (333 mg, 0.92 mmol, 1.1 equiv.) was added and the reaction mixture was stirred to 85 °C for 48 hours. The reaction mixture was cooled down to room temperature and the solvent was removed under vacuum. The crude material was purified by "flash" column chromatography (SiO_2, DCM/EtOAc, 70/30 to 50/50) yielding pure **TATA *p*-U** (560 mg, 71% yield) as a colorless solid. ^1H NMR (400 MHz, DMSO-d_6): δ (ppm)= 10.12 (s, 1H, NH), 9.37 (s, 1H, NH), 8.94 (s, 1H, NH), 8.38 (t, *J*= 5.7 Hz, 2H, NH), 8.14 (s, 2H, Ar_{CF3}), 7.94 (apparent d, *J*= 8.3 Hz, 2H, CH_{arom}), 7.83 (apparent d, *J*= 8.3 Hz, 4H, CH_{arom}), 7.70 (apparent d, *J*= 8.6 Hz, 2H, CH_{arom}), 7.62 (s, 1H, Ar_{CF3}), 7.46 (apparent d, *J*= 8.6 Hz, 2H, CH_{arom}), 7.15-7.12 (m, 6H, CH_{arom}), 3.24 (q, *J*= 6.6 Hz, 4H, $NHCH_2$), 1.50 (qnt, *J*= 6.7 Hz, 4H, $NHCH_2CH_2$), 1.31-1.20 (m, 20H, $NHCH_2CH_2$), 0.85 (t, *J*= 6.6 Hz, 6H, CH_3) ; $^{13}C\{^1H\}$ NMR (101 MHz, DMSO-d_6): δ (ppm)= 165.33, 164.45, 152.46, 149.09, 148.54, 142.00, 134.69, 134.23, 130.72 (q, *J*= 32.7 Hz), 129.86, 129.54, 129.34, 128.87, 123.35 (q, *J*= 272.8 Hz, CF_3), 123.51, 122.99, 120.98, 119.24, 117.91 (br s), 114.20 (br s), 39.19, 31.29, 29.20, 28.80, 28.71, 26.54, 22.12, 13.95 ; $^{19}F\{^1H\}$ NMR (377 MHz, DMSO-d_6): δ (ppm)= −61.65 ; HMRS (ESI): m/z calculated for $C_{52}H_{58}F_6N_6O_4H$ $[M+H]^+$: 945.4496, found: 945.4494 (0.3 ppm) ; FT-IR : υ (cm^{-1})= 3308, 3106, 3045, 2954, 2928, 2871, 2856, 2360, 2341, 1704, 1635, 1598, 1556, 1515, 1498, 1473, 1442, 1404, 1389, 1309, 1278, 1228, 1180, 1135, 1112, 1097, 1048, 1016.

Synthesis of TATA TU

Reaction between compound **5** and *N*-(4-aminophenyl)-*N'*-[3,5-bis(trifluoromethyl)phenyl]thiourea under various conditions afforded **TATA TU** in limited and unsatisfactory reproducible yields (from 5% to 40%, 250 mg of **TATA TU** was isolated in total from different attempts). Accordingly, only the most reproducible route is described below.

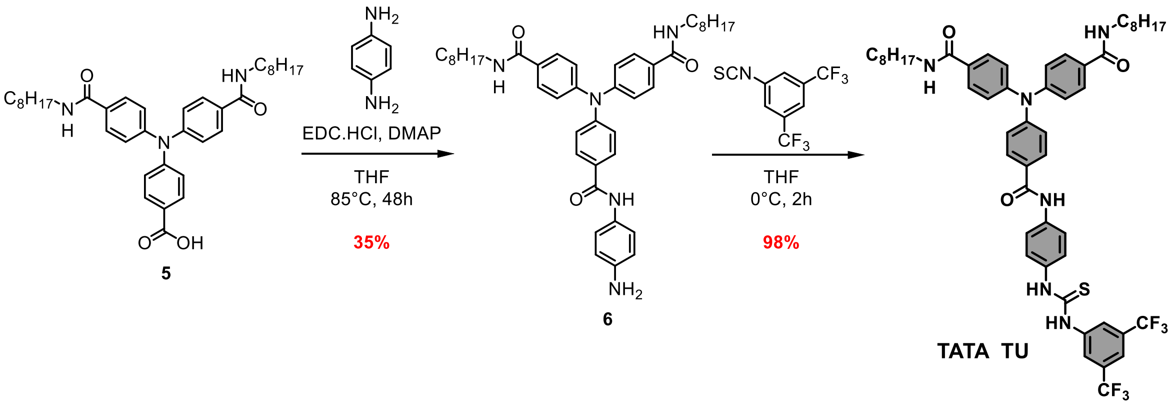

Compound 6: In an oven-dried 20 mL Schlenk flask under argon atmosphere, compound **5** (100 mg, 0.17 mmol, 1 equiv.), EDC•HCl (55 mg, 0.28 mmol, 1.7 equiv.) and DMAP (35 mg, 0.28 mmol, 1.7 equiv.) were solubilized in THF (4 mL). Then *p*-phenylenediamine (72 mg, 0.67 mmol, 4 equiv.) was added and the reaction mixture was heated to 85 °C for 48 hours. The reaction mixture was cooled down to room temperature and the solvent was removed under vacuum. The obtained solid was dissolved into ethyl acetate and washed successively with aqueous $NaHCO_3$ (0.1M) and brine. The organic phase was dried over anhydrous Na_2SO_4, filtered and evaporated under vacuum. The crude material was purified by "flash" column chromatography (SiO_2, DCM/EtOAc 50/50) yielding compound **6** (40 mg, 35%) as a dark yellow powder. Given the instability of the compound, the product was only characterized by ^{1}H NMR and engaged in the following step. ^{1}H NMR (400 MHz, acetone-d_6): δ (ppm)= 9.17 (s, 1H, NH), 7.93 (apparent d, *J*= 8.7 Hz, 2H, CH_{arom}), 7.87 (apparent d, *J*= 8.6 Hz, 4H, CH_{arom}), 7.68 (t, *J*= 5.5 Hz, 2H, NH), 7.50 (apparent d, *J*= 8.7 Hz, 2H, CH_{arom}), 7.16-7.12 (m, 6H, CH_{arom}), 6.65 (apparent d, *J*= 8.7 Hz, 2H), 4.48 (br s, 2H, NH_2), 3.39 (q, *J*= 6.7 Hz, 4H, $NHCH_2$), 1.61 (qnt, *J*= 7.0 Hz, 4H, $NHCH_2CH_2$), 1.42-1.21 (m, 20H, CH_2), 0.88 (t, *J*= 7.0 Hz, 6H, CH_3).

TATA TU: In an oven-dried 20 mL Schlenk flask under argon atmosphere, compound **6** (36 mg, 52 µmol, 1 equiv.) was dissolved in dry THF (1 mL) and the solution was cooled down to 0°C. A solution of 3,5-bis(trifluoromethyl)phenyl isothiocyanate (12 µL, 63 µmol, 1.2 equiv.) in THF (0.5 mL) was then poured to this solution via a syringe pump over 30 min. After full addition of the reagent, the reaction mixture was warmed to room temperature and stirred for additional 2 h. After removal of the solvent under reduced pressure, the crude material was purified by "flash" column chromatography (SiO_2, DCM/EtOAc, 65/35) yielding **TATA TU** (49 mg, 98%) as a yellow solid. ^{1}H NMR (400 MHz, DMSO-d_6): δ (ppm)= 10.26 (s, 1H, NH), 10.24 (s, 1H, NH), 10.14 (s, 1H,

NH), 8.38 (t, *J*= 5.7 Hz, 2H, NH), 8.27 (s, 2H, Ar_{CF3}), 7.94 (apparent d, *J*= 8.5 Hz, 2H, CH_{arom}), 7.83 (apparent d, *J*= 8.5 Hz, 4H, CH_{arom}), 7.80-7.77 (m, 3H, CH_{arom}+ Ar_{CF3}), 7.40 (apparent d, *J*= 8.9 Hz, 2H, CH_{arom}), 7.15-7.10 (m, 6H, CH_{arom}), 3.24 (q, *J*= 6.6 Hz, 4H, $NHCH_2$), 1.51 (qnt, *J*= 7.0 Hz, 4H, $NHCH_2CH_2$), 1.31-1.27 (m, 20H, CH_2), 0.83 (t, *J*= 6.8 Hz, 6H, CH_3) ; $^{13}C\{^1H\}$ NMR (101 MHz, DMSO-d_6): δ (ppm)= 179.76, 165.31, 164.68, 149.23, 148.51, 141.99, 136.74, 133.88, 129.94 (q, *J*= 32.7 Hz), 129.89, 129.43, 129.34, 128.87, 124.75, 123.55, 123.28 (q, *J*= 272.6 Hz, CF_3), 122.97, 120.62, 116.77 (br s), 39.18, 31.28, 29.19, 28.79, 28.70, 26.53, 22.11, 13.96 ; $^{19}F\{^1H\}$ NMR (377 MHz, DMSO-d_6): δ (ppm)= −61.49 ; HMRS (ESI): m/z calculated for $C_{52}H_{58}F_6N_6O_3SH$ $[M+H]^+$: 961.4268, found: 961.4266 (0.2 ppm) ; FT-IR : υ (cm^{-1})= 3300, 3095, 3045, 2959, 2926, 2855, 2360, 2342, 1638, 1598, 1543, 1513, 1498,1472, 1456, 1408, 1384, 1311, 1277, 1242, 1179, 1136, 1108, 1016, 1001.

Synthesis of chiral anions

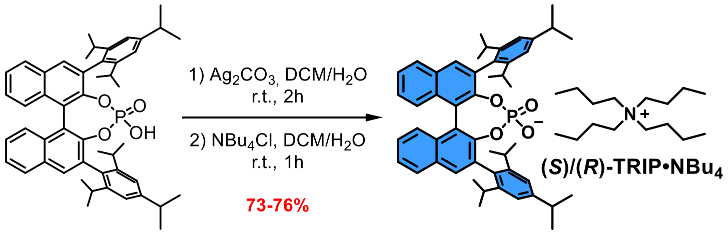

(*S*)-TRIP•NBu₄: Adapted from the literature,[10] Ag_2CO_3 (91.6 mg, 0.33 mmol, 1 equiv.) in deionized water (0.1 M) was added to a solution of (*S*)-3,3'-bis(2,4,6-triisopropylphenyl)-1,1'-binaphthyl-2,2'-diyl hydrogen phosphate (250 mg, 0.33 mmol, 1 equiv.) in DCM (0.1 M) in the dark. The reaction mixture was stirred at room temperature for 2h and then filtered over Celite. The solid was washed with DCM. The organic phase was recovered. The aqueous layer was extracted with DCM (2 x 5 mL). The combined organic phases were dried over anhydrous Na_2SO_4, filtered and evaporated under vacuum to afford a solid. A solution of NBu_4Cl (1 equiv.) in 1 mL of distilled H_2O was added to a solution of this solid in 5 mL of DCM. The reaction mixture was stirred vigorously for 1h. The aqueous layer was extracted twice with 10 mL of DCM. The combined organic phases were dried over anhydrous Na_2SO_4, filtered and evaporated under vacuum yielding **(*S*)-TRIP•NBu₄** (240 mg, 73%) as a colorless solid. ^1H NMR (400 MHz, CD_2Cl_2): δ (ppm)= 7.83 (d, *J*= 8.2 Hz, 2H, $CH_{arom.}$), 7.68 (s, 2H, $CH_{arom.}$), 7.38−7.32 (m, 2H, $CH_{arom.}$), 7.18−7.16 (m, 4H, $CH_{arom.}$), 7.14 (d, *J*= 1.9 Hz, 2H, $CH_{arom.}$), 7.04 (d, *J*= 1.9 Hz, 2H, $CH_{arom.}$), 3.11-2.89 (m, 12H, 4×NCH_2+4×CH), 2.73 (septet, *J*= 6.8 Hz, 2H, CH), 1.43 (qnt, *J*= 7.9 Hz, 8H, CH_2), 1.32 (d, *J*= 6.9 Hz, 12H, $CHCH_3$), 1.28 (d, *J*= 6.6 Hz, 6H, $CHCH_3$), 1.24-1.10 (m, 20H, 4×CH_2+4×$CHCH_3$), 0.90 (d, *J*= 6.8 Hz, 6H, $CHCH_3$), 0.85 (t, *J*= 7.3 Hz, 12H, CH_3) ; $^{13}C\{^1H\}$ NMR (101 MHz, CD_2Cl_2): δ (ppm)= 149.71, 149.61, 148.61, 147.73, 147.59, 134.59, 133.73, 133.33, 131.64, 130.40, 128.11, 127.34, 125.43, 124.30, 123.46, 121.25, 120.17, 58.91, 34.70, 31.46, 30.98, 26.51, 24.93, 24.39, 24.33, 24.17, 24.00, 23.34, 19.84, 13.73 ; $^{31}P\{^1H\}$ NMR (101 MHz, CD_2Cl_2): δ (ppm)= 4.20 ; HMRS (ESI): *m/z* calculated for $C_{50}H_{56}O_4PHNa$

[M─NBu_4+Na+H]$^+$: 775.3887, found: 775.3887 (0 ppm) ; *m/z* calculated for NBu_4 cation $C_{16}H_{36}N$: 242.2842, found: 242.2842 (0 ppm).

(*R*)-TRIP•NBu₄ was synthesized following the same procedure using (*R*)-3,3'-bis(2,4,6-triisopropylphenyl)-1,1'-binaphthyl-2,2'-diyl hydrogen phosphate (250 mg, 76%); analytical data are identical to **(*S*)-TRIP•NBu₄**.

Selected chiral GC traces (Figures S16-S17)

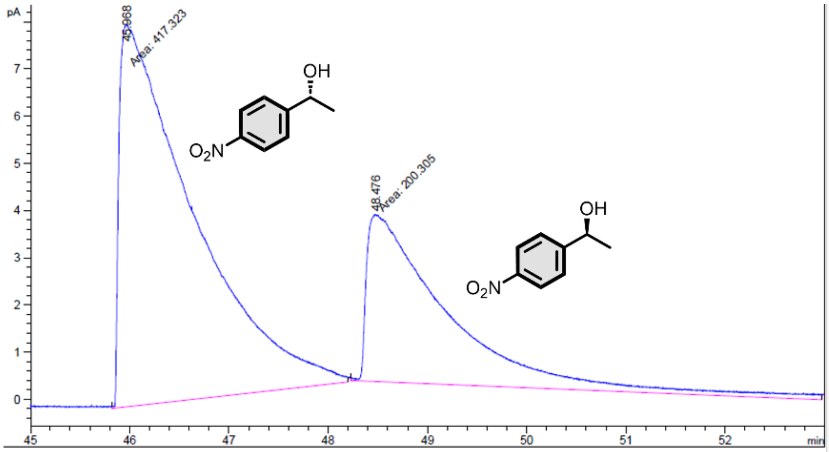

Peak #	RetTime [min]	Type	Width [min]	Area [pA*s]	Height [pA]	Area %
1	45.968	MM	0.8593	417.32257	8.09425	67.56869
2	48.476	MM	0.9417	200.30455	3.54511	32.43131

Figure S16. Chiral GC analysis of entry 6, Table 1. Composition: **TATA PCF3**/[Cu]= 4, **TATA U**, **(*S*)-TRIP•NBu$_4$** (fs= 50%). −35% *ee*.

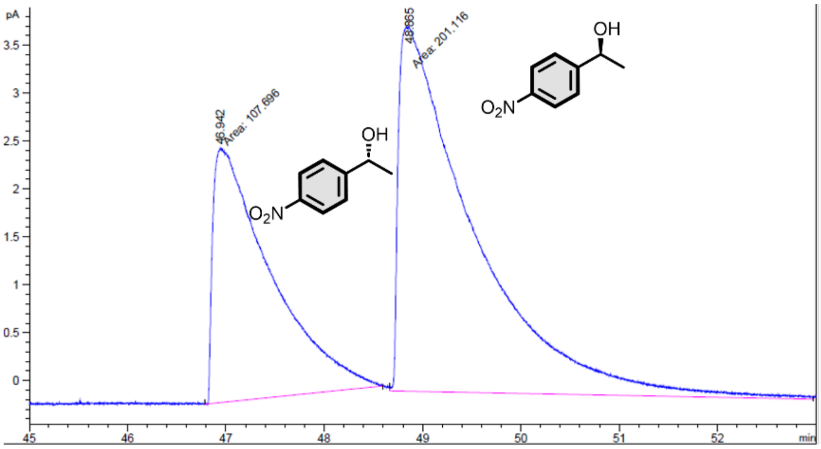

Peak #	RetTime [min]	Type	Width [min]	Area [pA*s]	Height [pA]	Area %
1	46.942	MM	0.6756	107.69601	2.65698	34.87429
2	48.865	MM	0.8807	201.11603	3.80613	65.12571

Figure S17. Chiral GC analysis of entry 7, Table 1. Composition: **TATA PCF3**/[Cu]= 4, **TATA U**, **(*R*)-TRIP•NBu$_4$** (fs= 50%). +30% *ee*.

NMR spectra (Figures S18-S58)

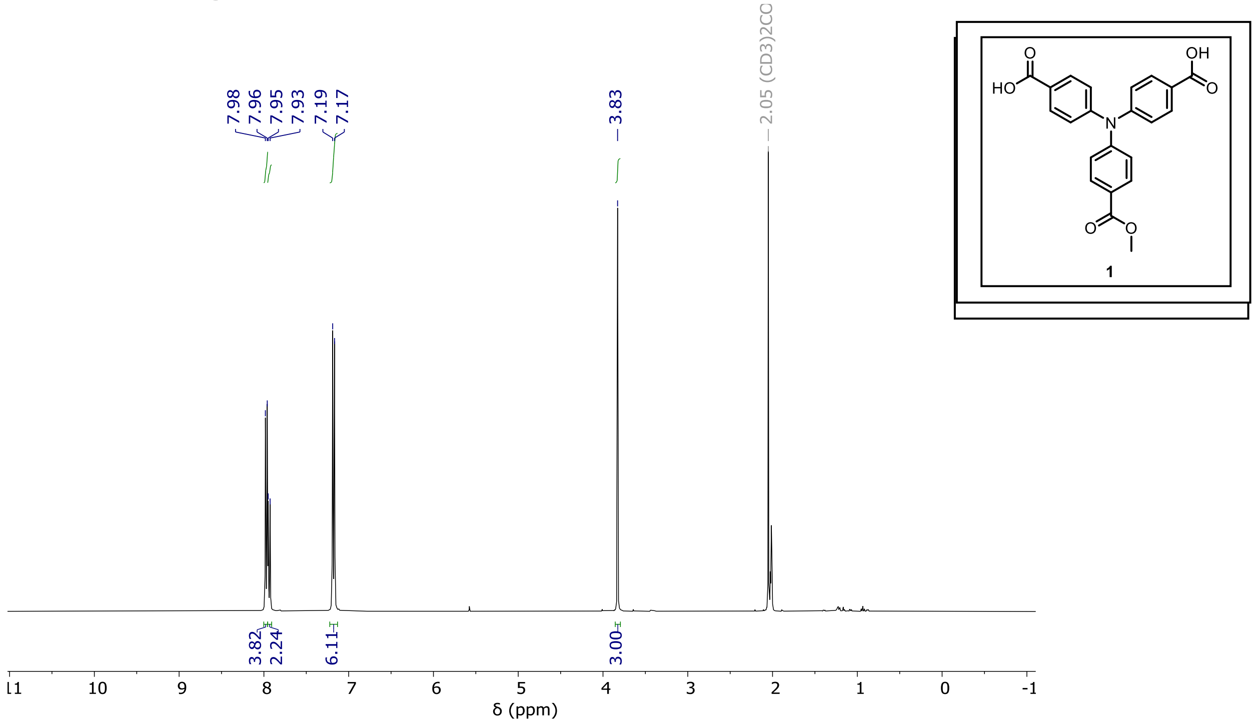

Figure S18. ^{1}H NMR (acetone-d$_6$) of compound **1**.

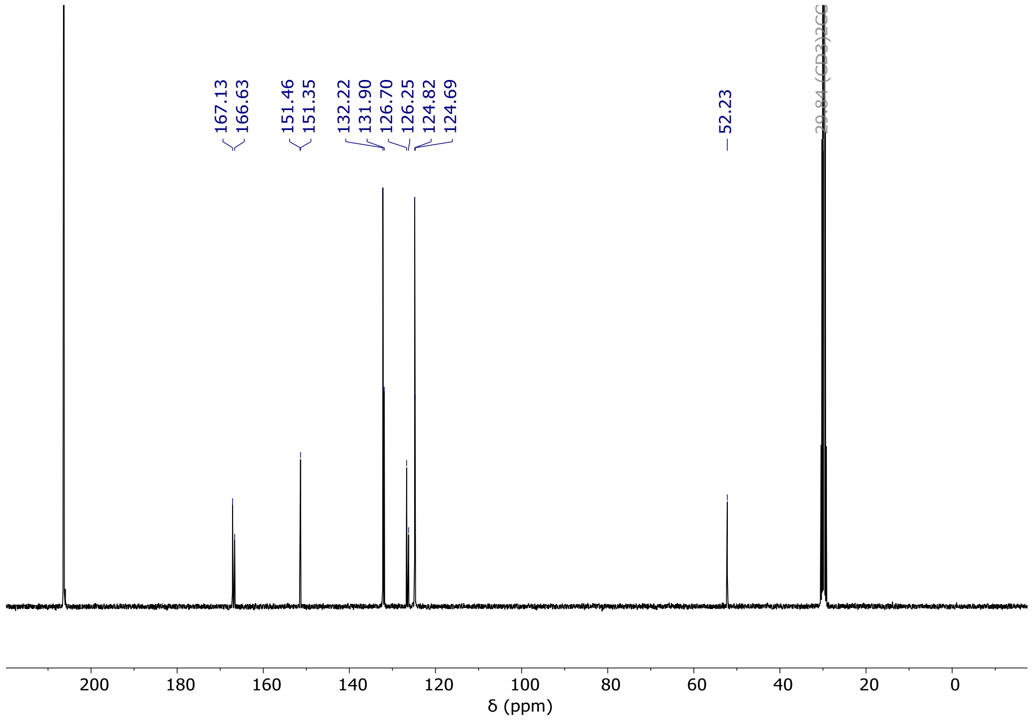

Figure S19. ^{13}C{^{1}H} NMR (acetone-d$_6$) of compound **1**.

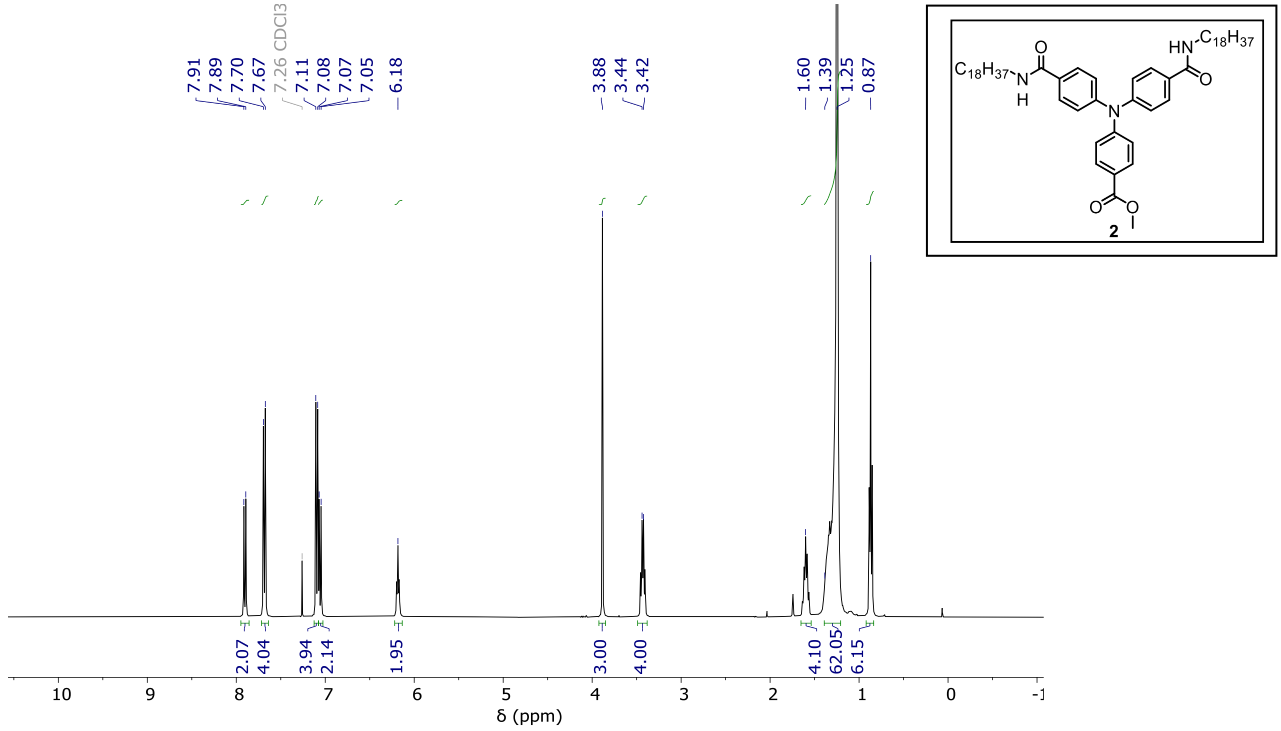

Figure S20. ^{1}H NMR ($CDCl_3$) of compound **2**.

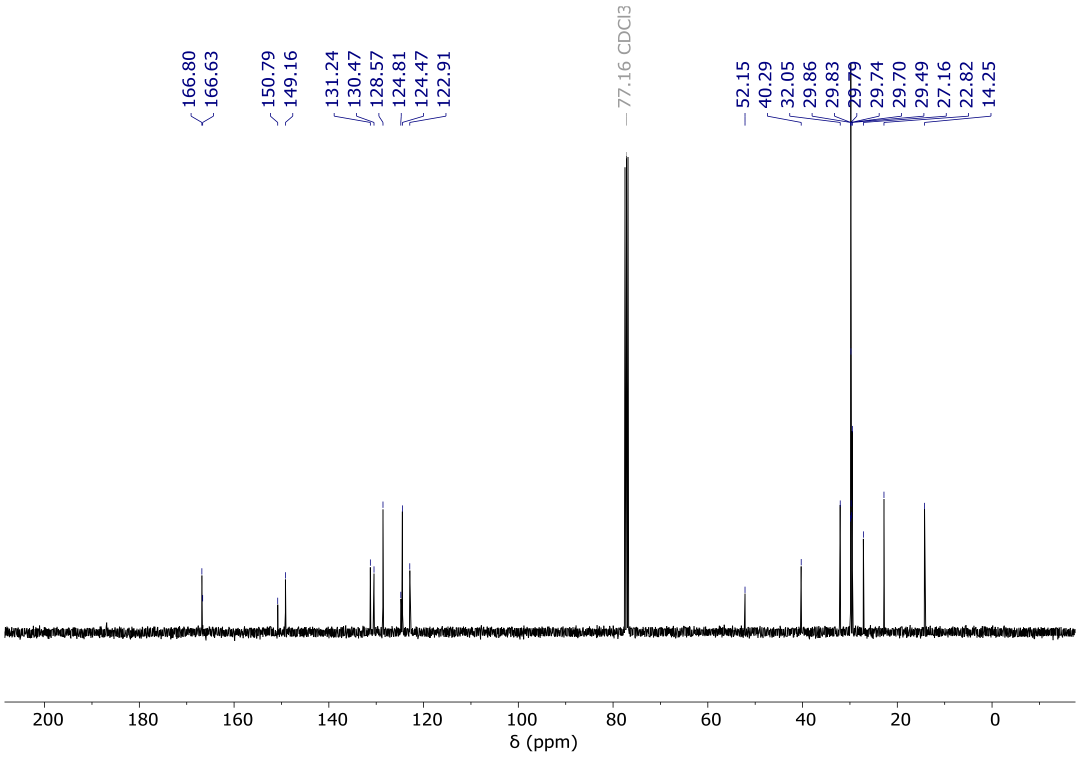

Figure S21. ^{13}C{^{1}H} NMR ($CDCl_3$) of compound **2**.

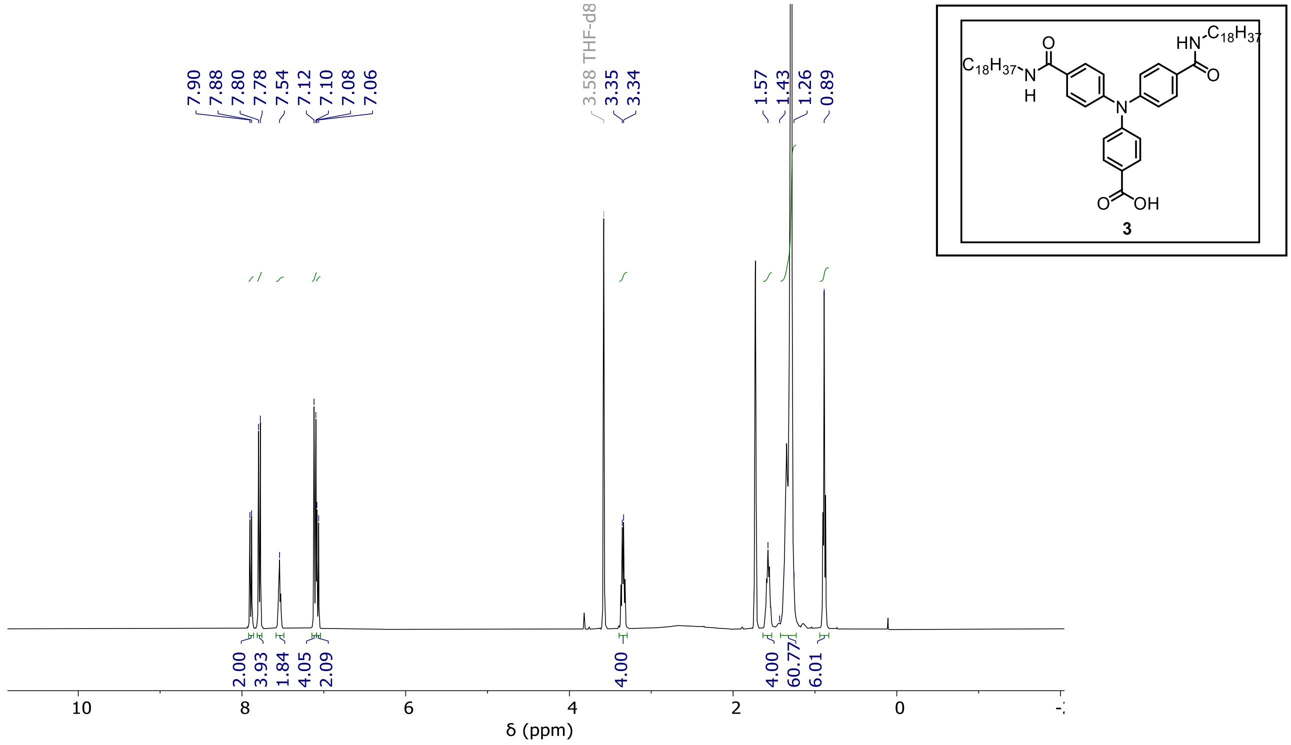

Figure S22. ^{1}H NMR (THF-d_8) of compound **3**.

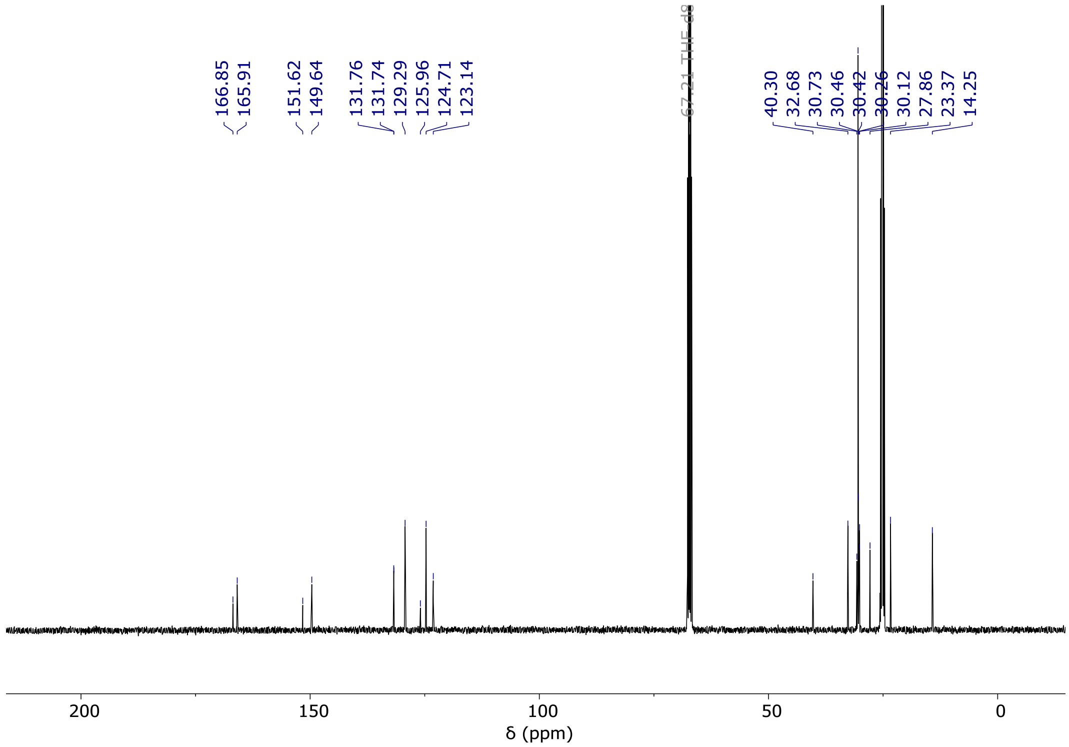

Figure S23. $^{13}C\{^1H\}$ NMR (THF-d_8) of compound **3**.

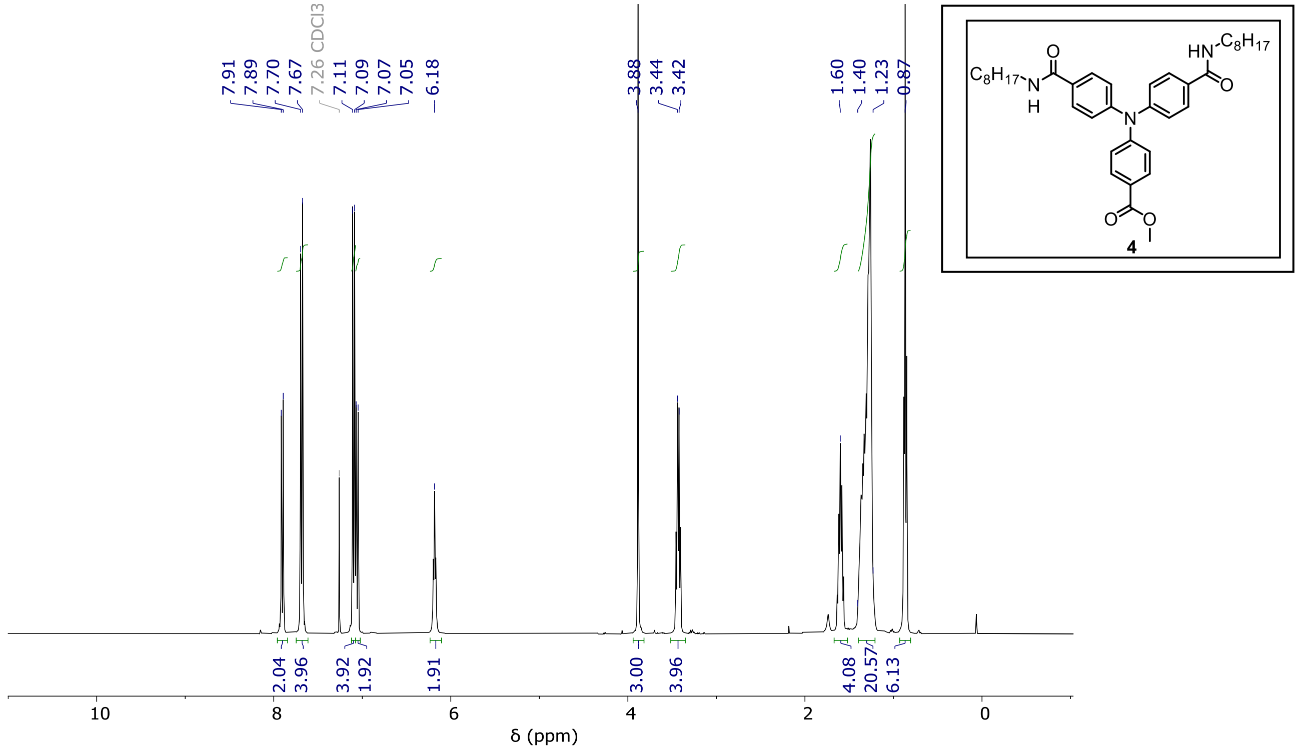

Figure S24. ^{1}H NMR ($CDCl_3$) of compound **4**.

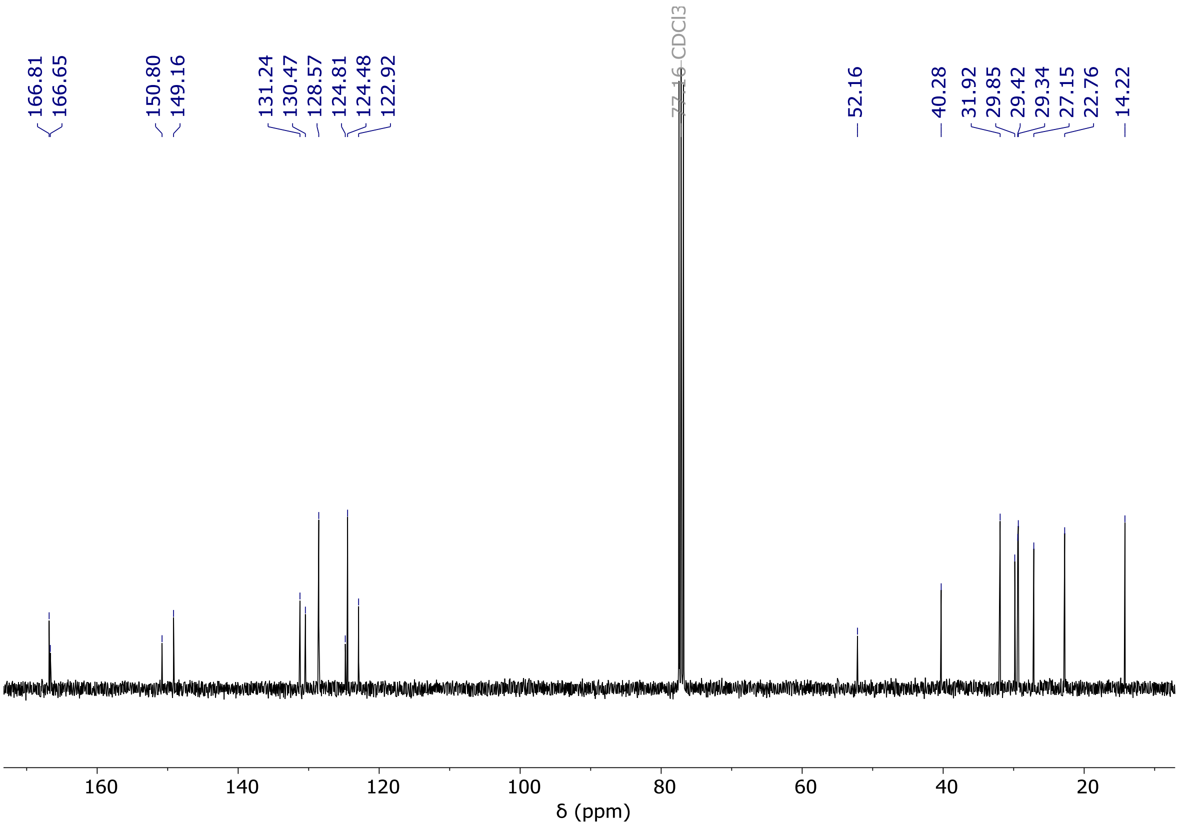

Figure S25. $^{13}C\{^{1}H\}$ NMR ($CDCl_3$) of compound **4**.

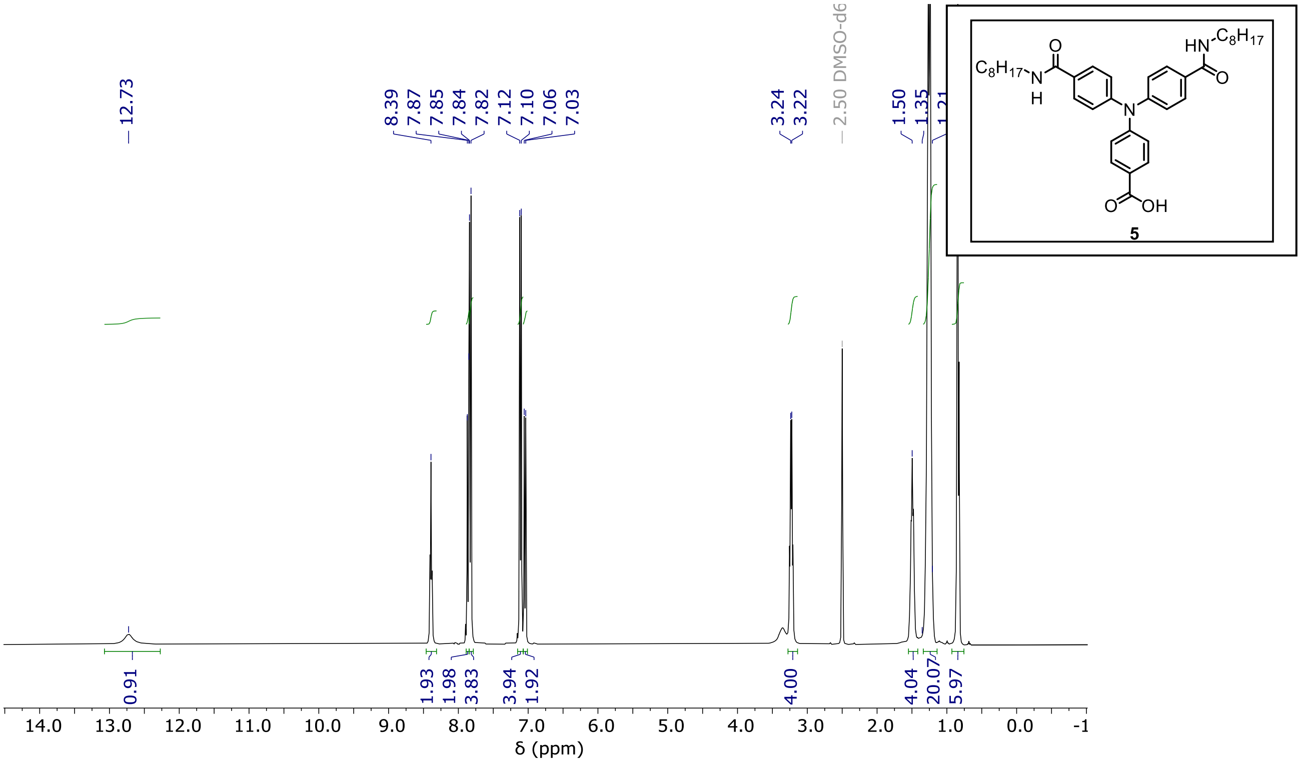

Figure S26. ^{1}H NMR (DMSO-d_6) of compound **5**.

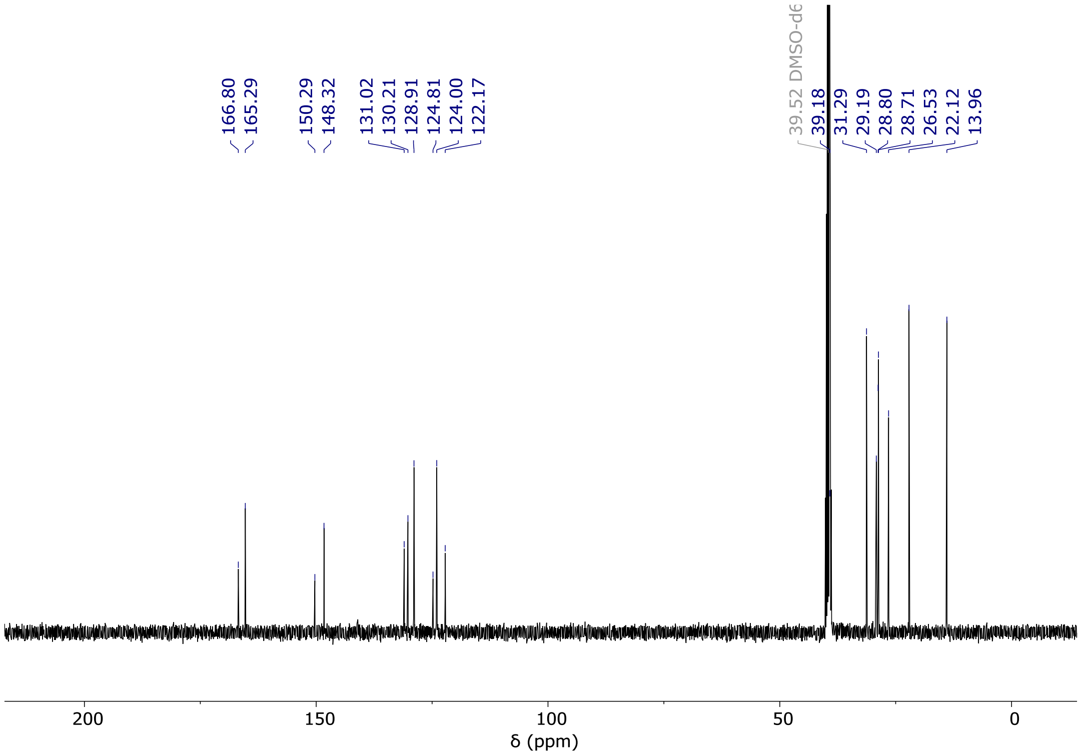

Figure S27. ^{13}C{^{1}H} NMR (DMSO-d_6) of compound **5**.

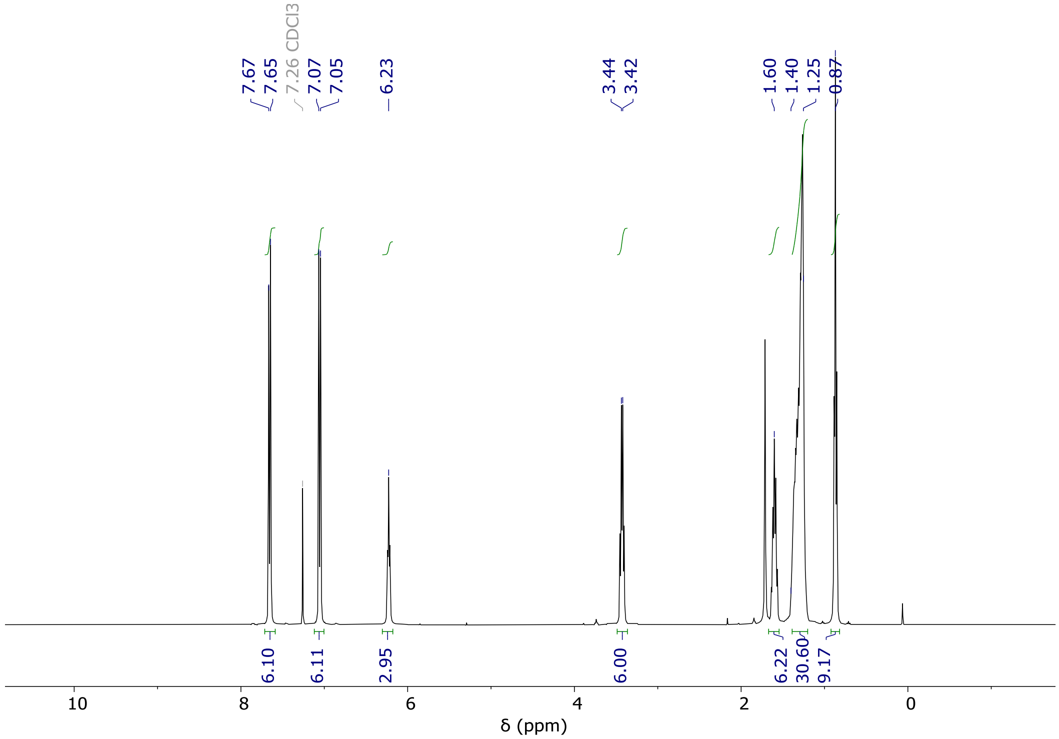

Figure S28. ^{1}H NMR ($CDCl_3$) of **TATA C8**.

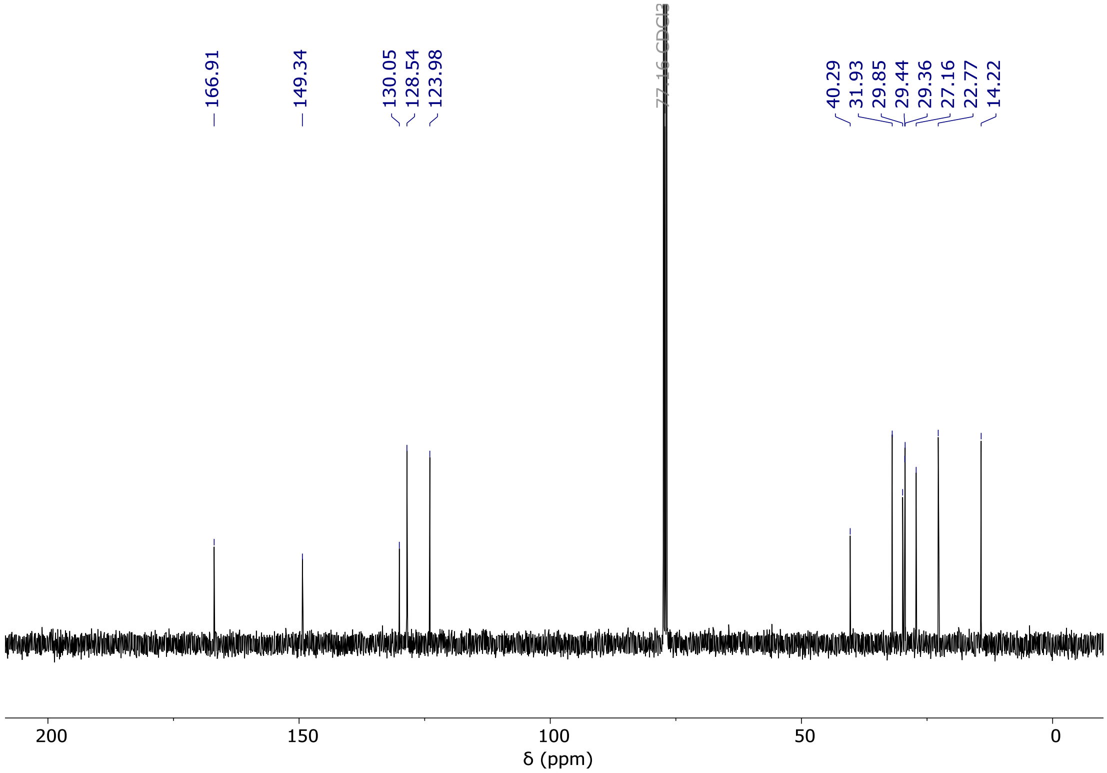

Figure S29. $^{13}C\{^{1}H\}$ NMR ($CDCl_3$) of **TATA C8**.

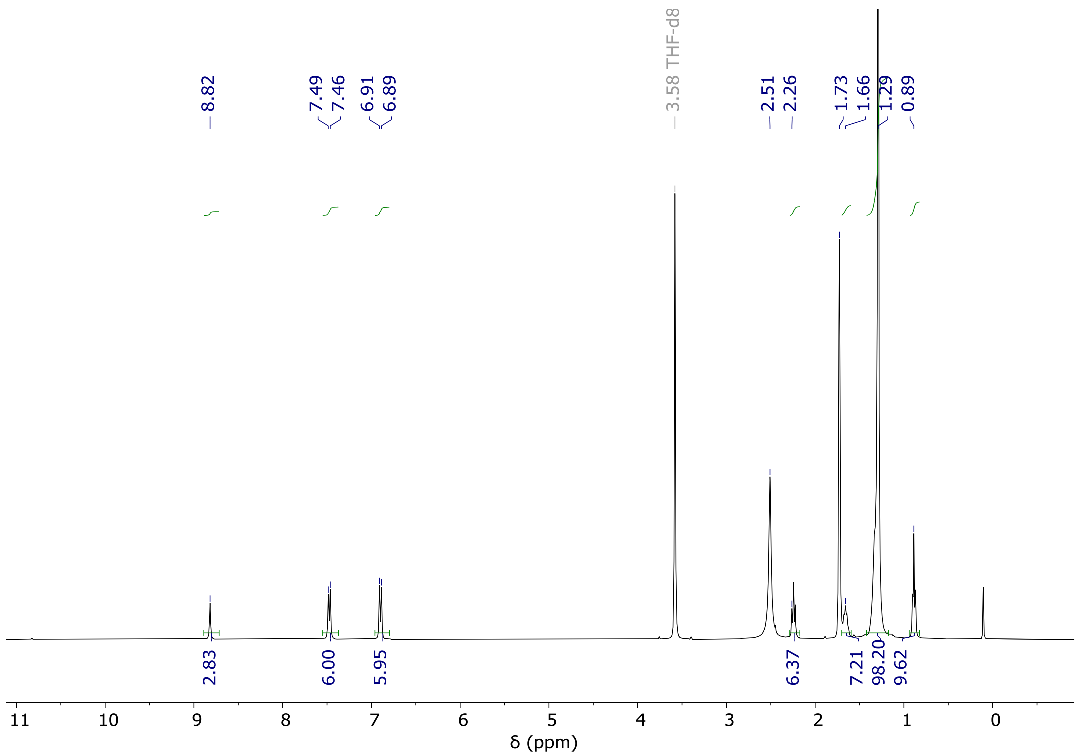

Figure S30. ^{1}H NMR (THF-d_8) of **TATA *N* C20**.

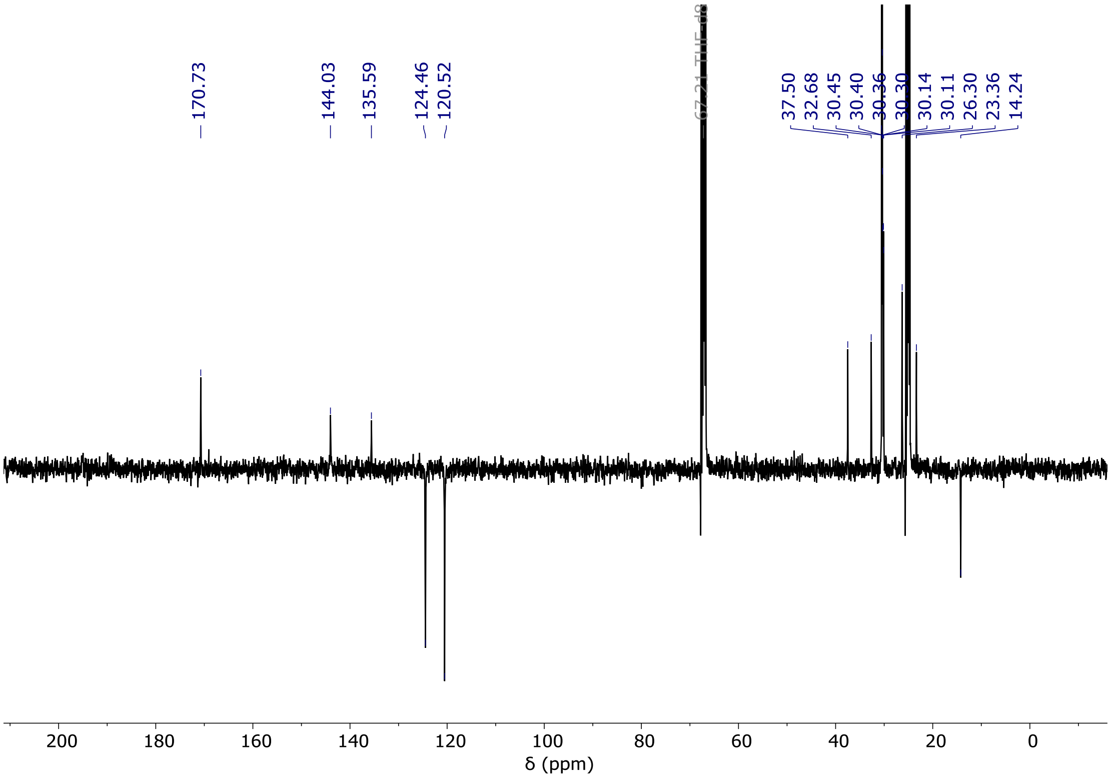

Figure S31. DEPT-135 (THF-d_8) of **TATA *N* C20**.

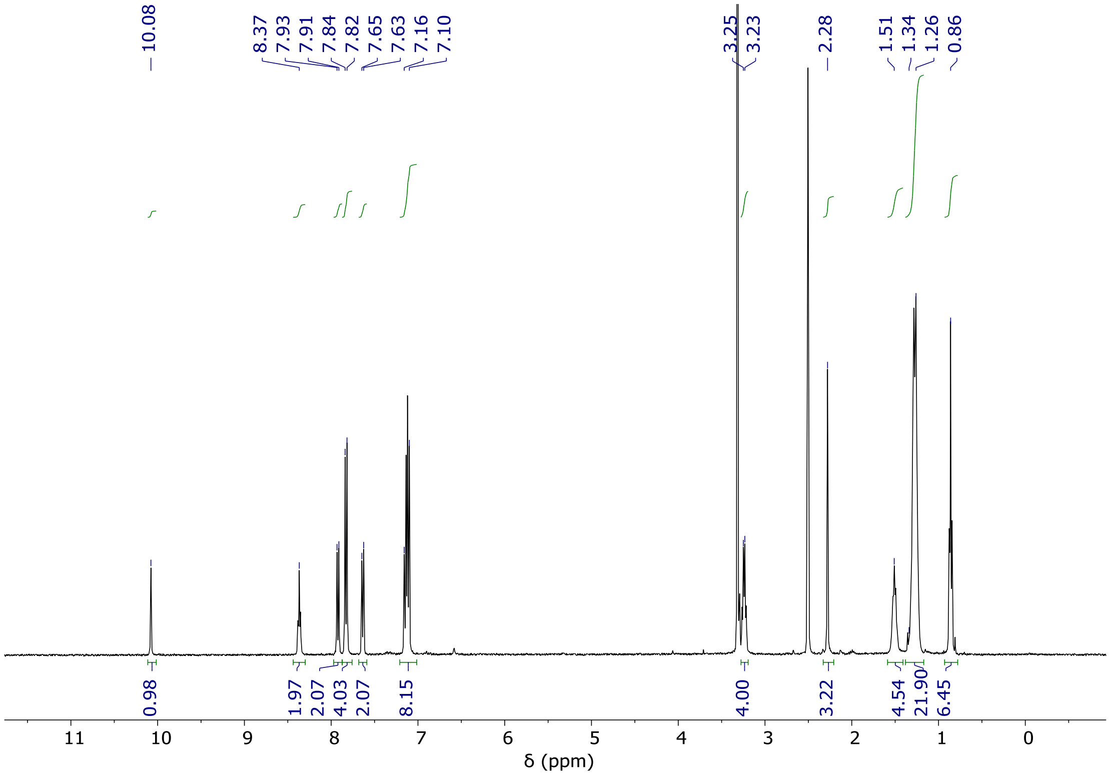

Figure S32. ^{1}H NMR (DMSO-d_6) of **TATA model**.

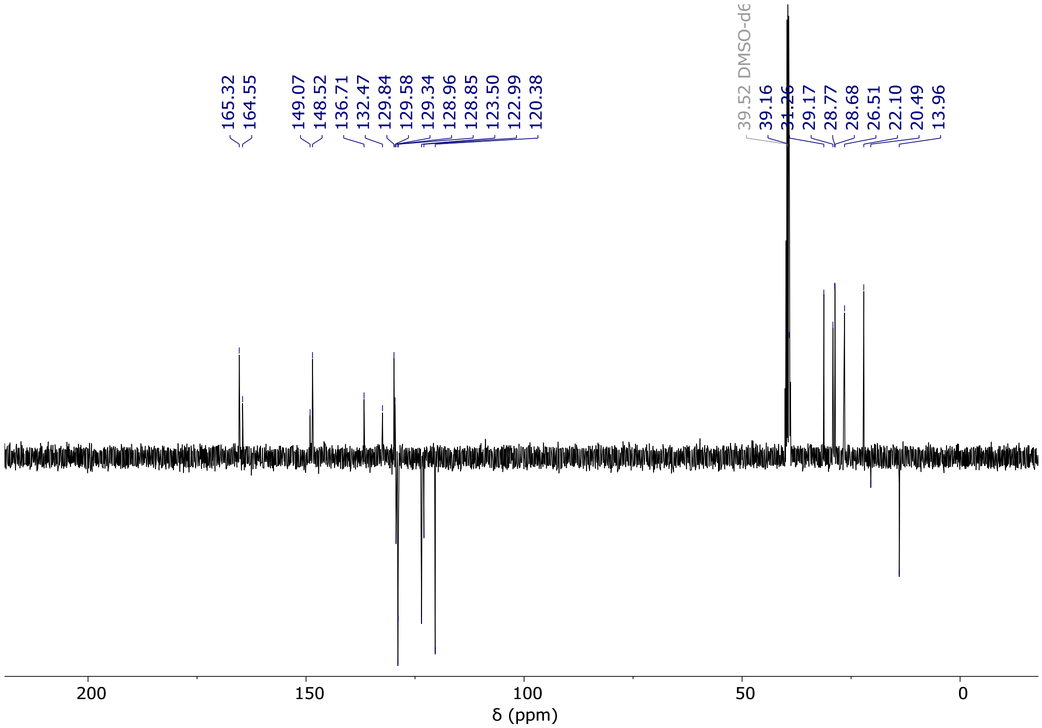

Figure S33. DEPT-135 (DMSO-d_6) of **TATA model**.

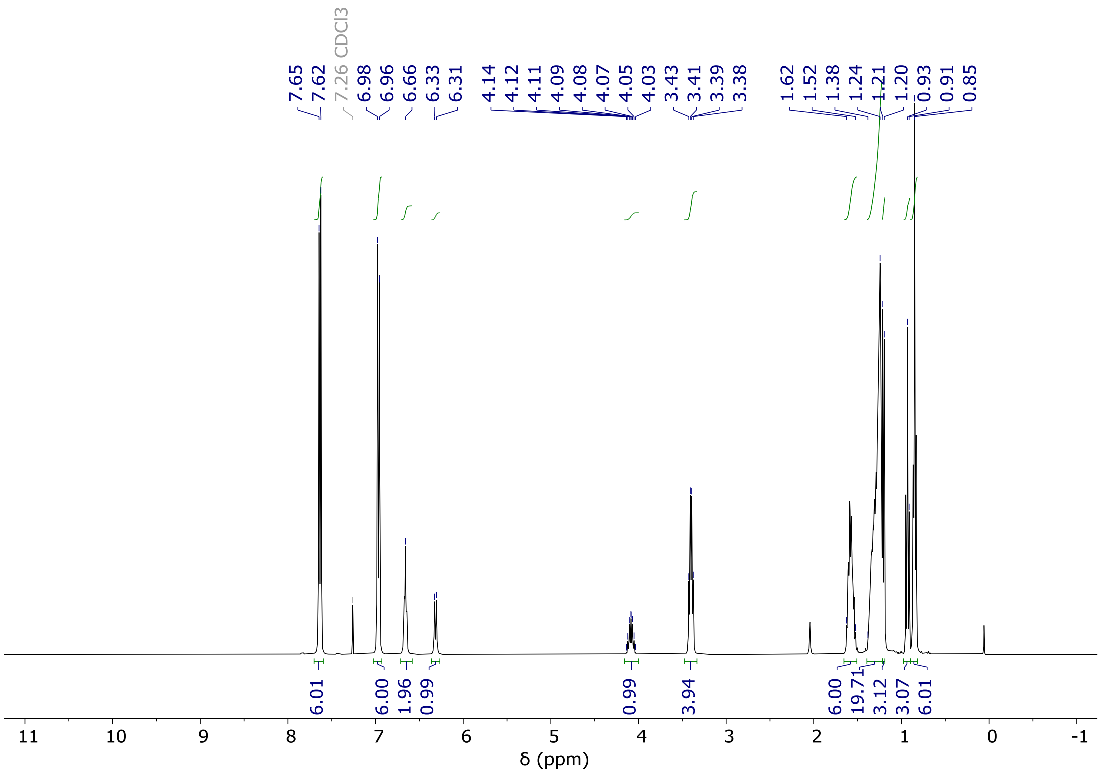

Figure S34. ^{1}H NMR ($CDCl_3$) of **(*S*)-TATA***.

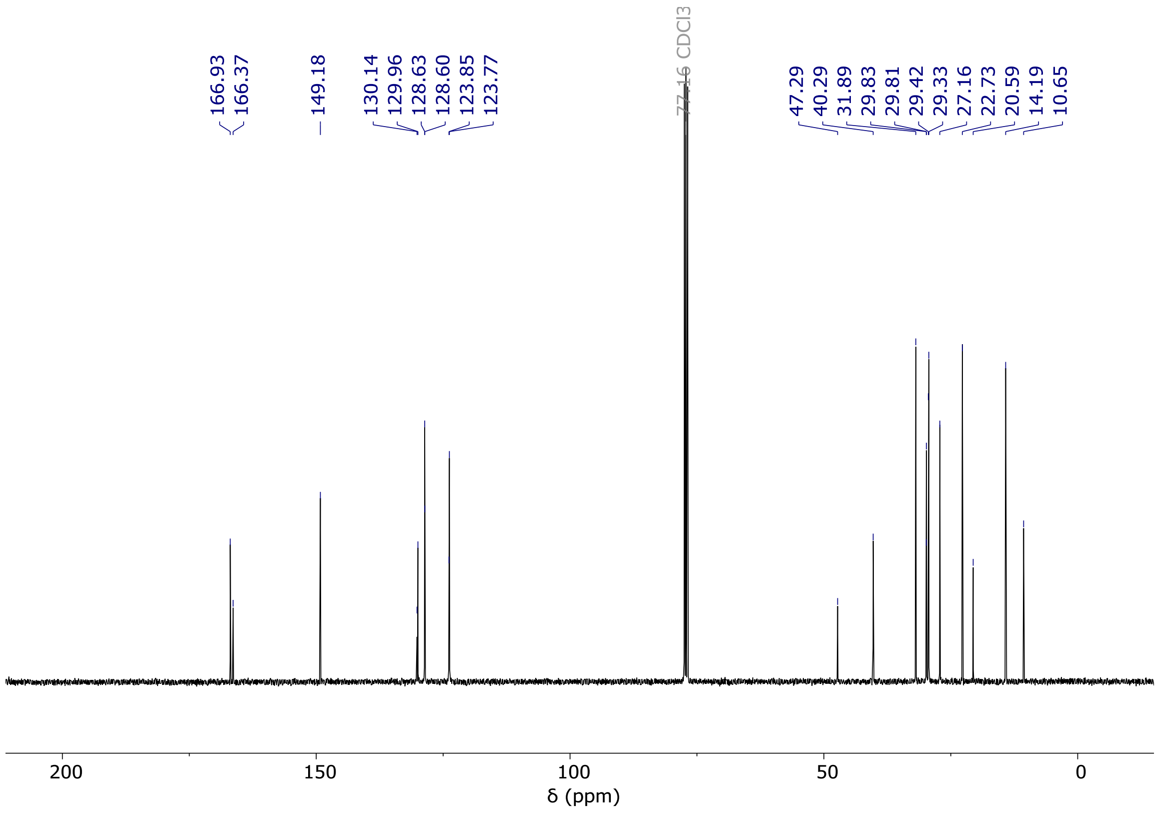

Figure S35. $^{13}C\{^{1}H\}$ NMR ($CDCl_3$) of **(*S*)-TATA***.

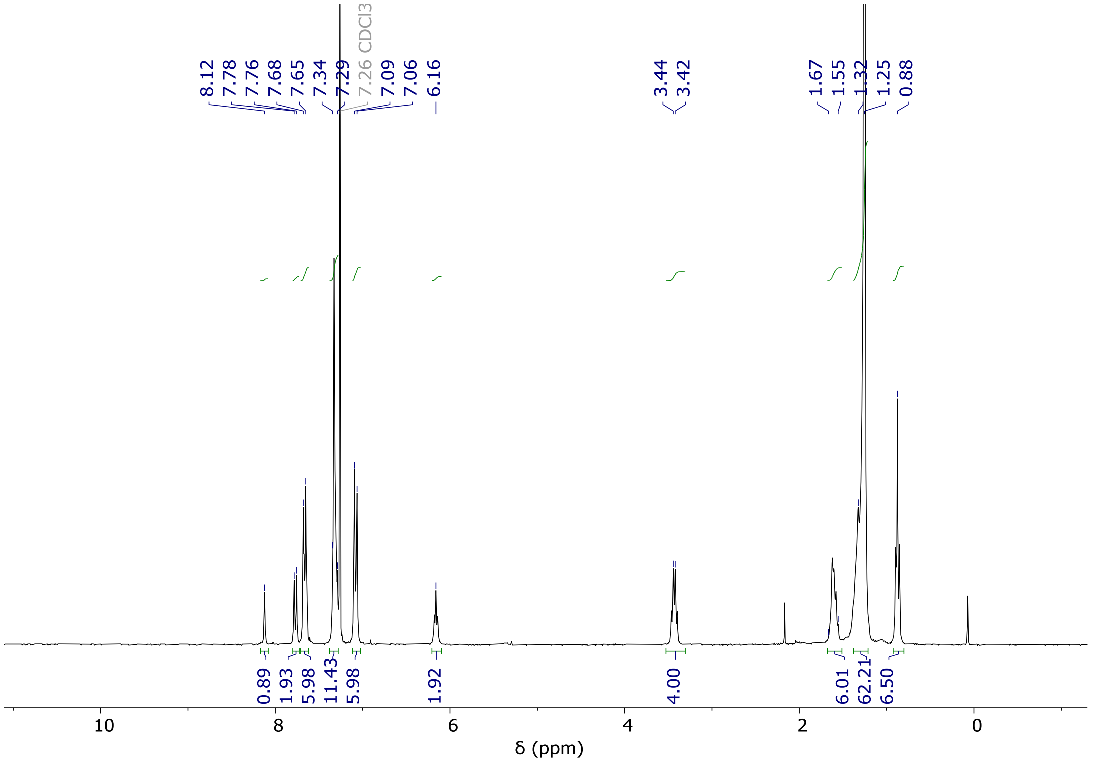

Figure S36. ^{1}H NMR ($CDCl_3$) of **TATA P**.

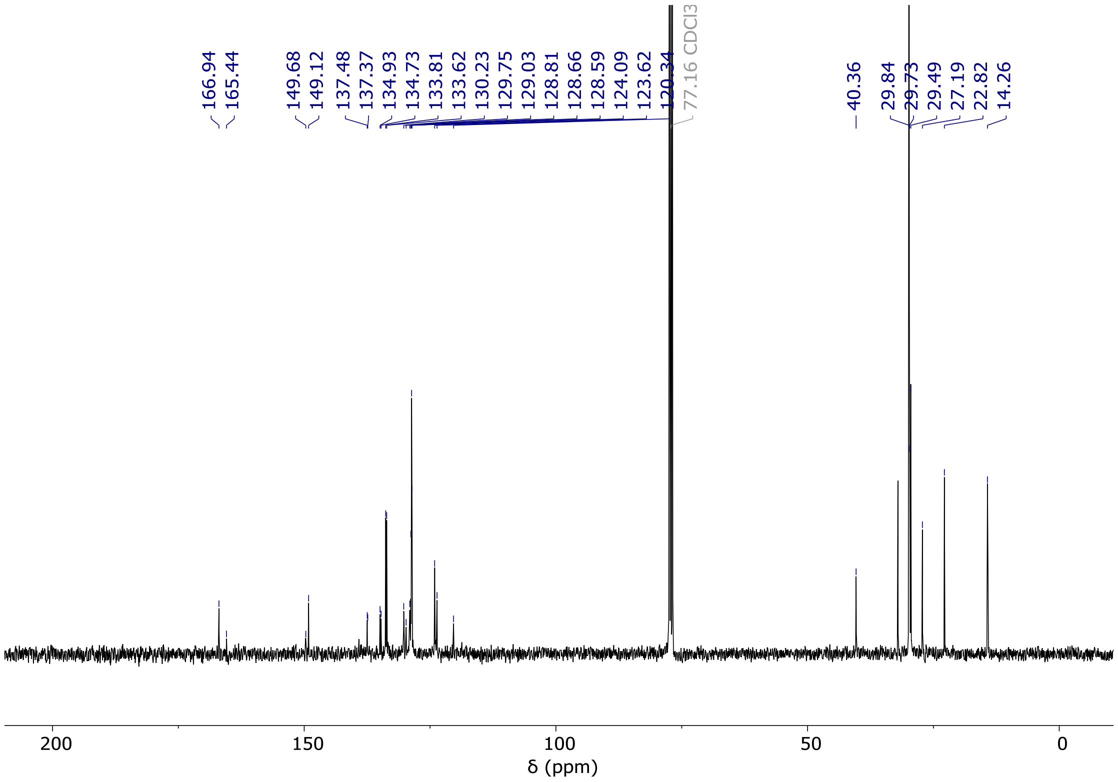

Figure S37. $^{13}C\{^{1}H\}$ NMR ($CDCl_3$) of **TATA P**.

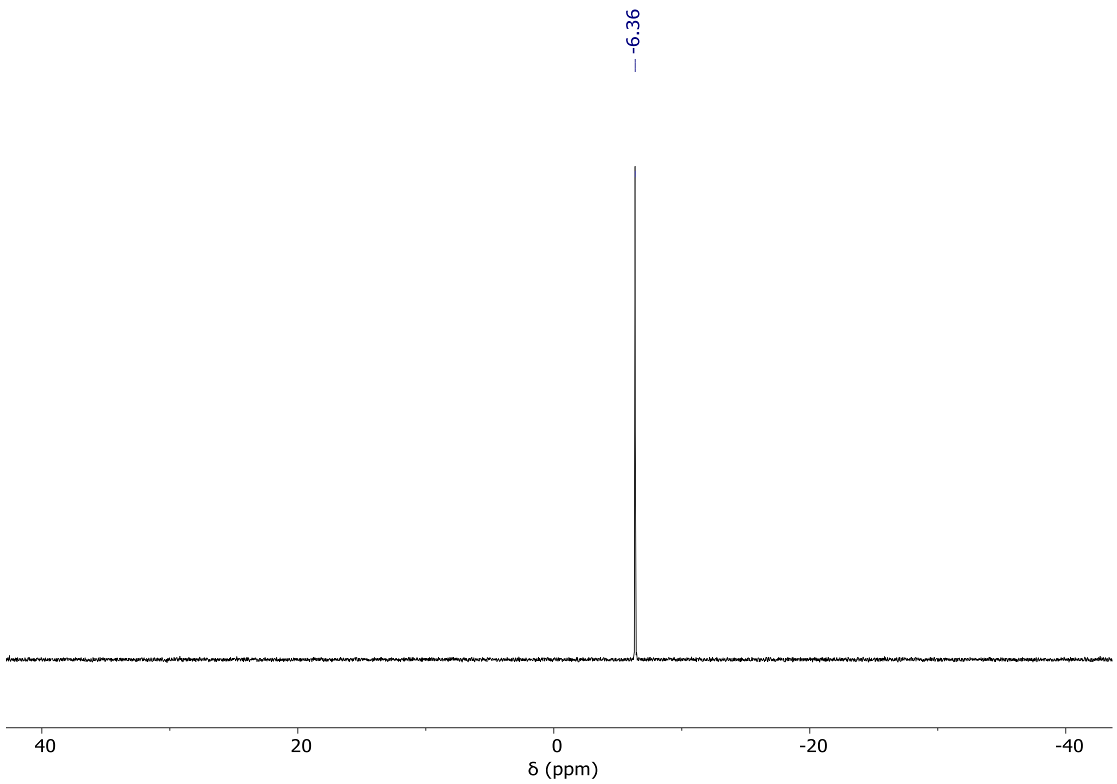

Figure S38. $^{31}P\{^{1}H\}$ NMR ($CDCl_3$) of **TATA P**.

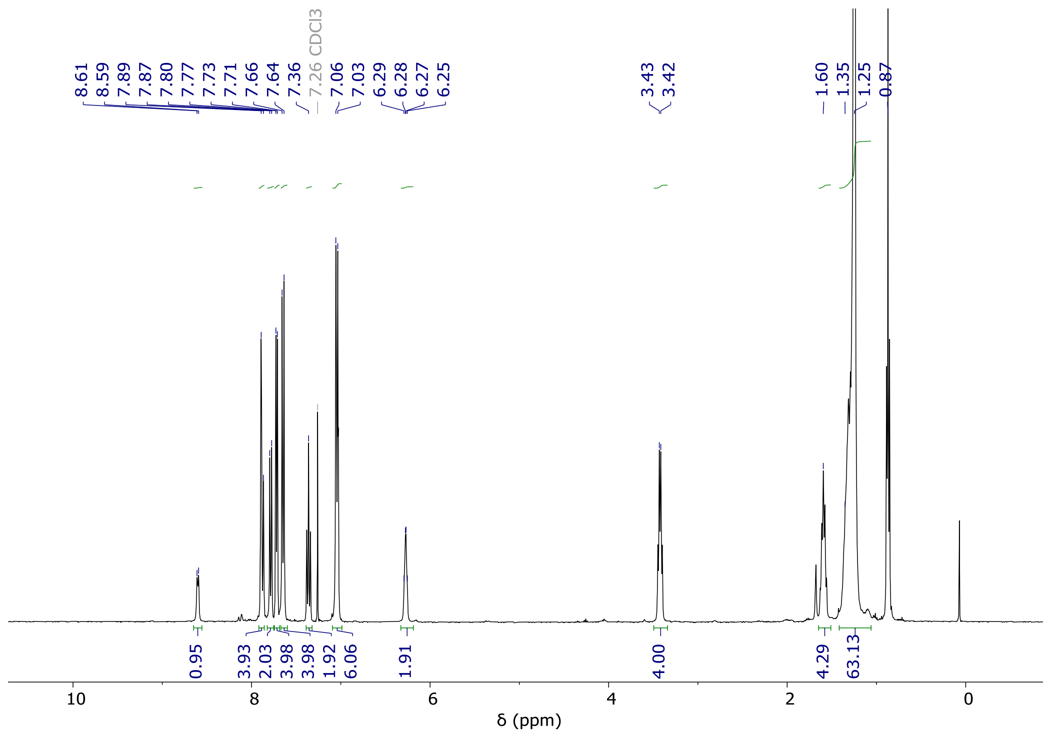

Figure S39. ^{1}H NMR ($CDCl_3$) of **TATA PCF3**.

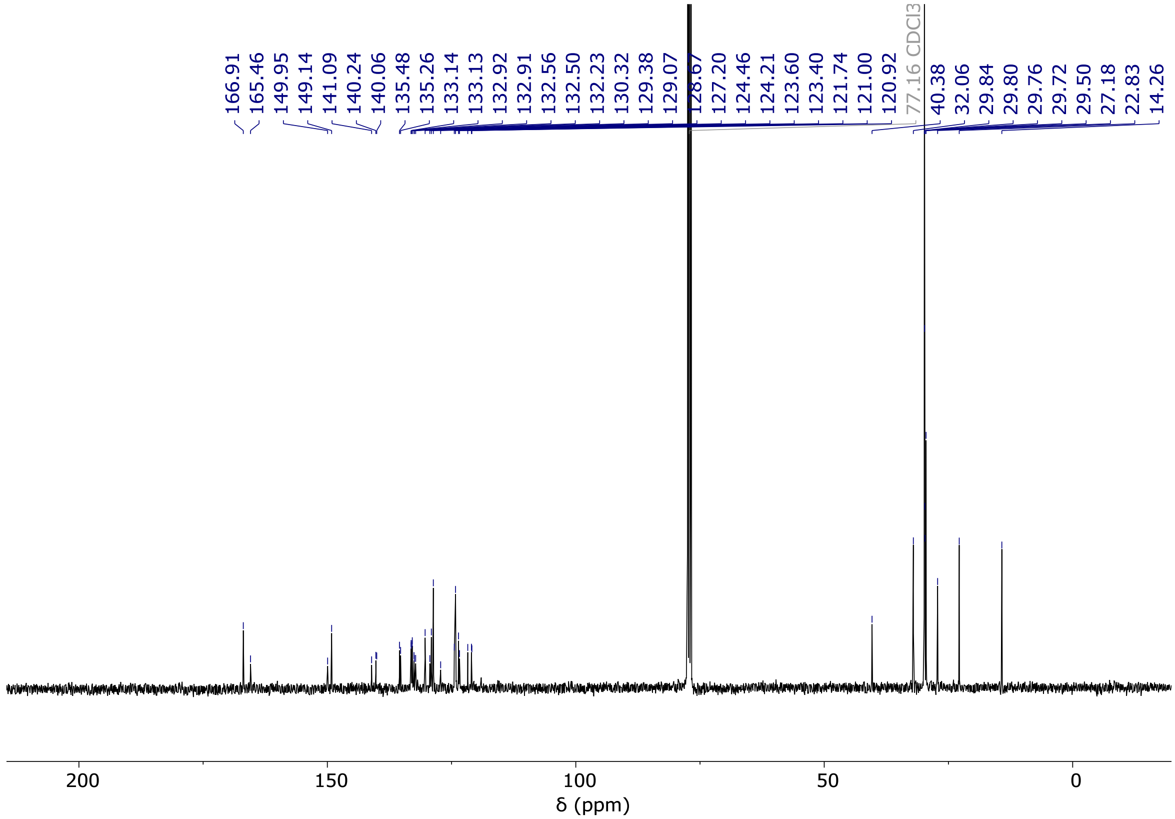

Figure S40. $^{13}C\{^{1}H\}$ NMR ($CDCl_3$) of **TATA PCF3**.

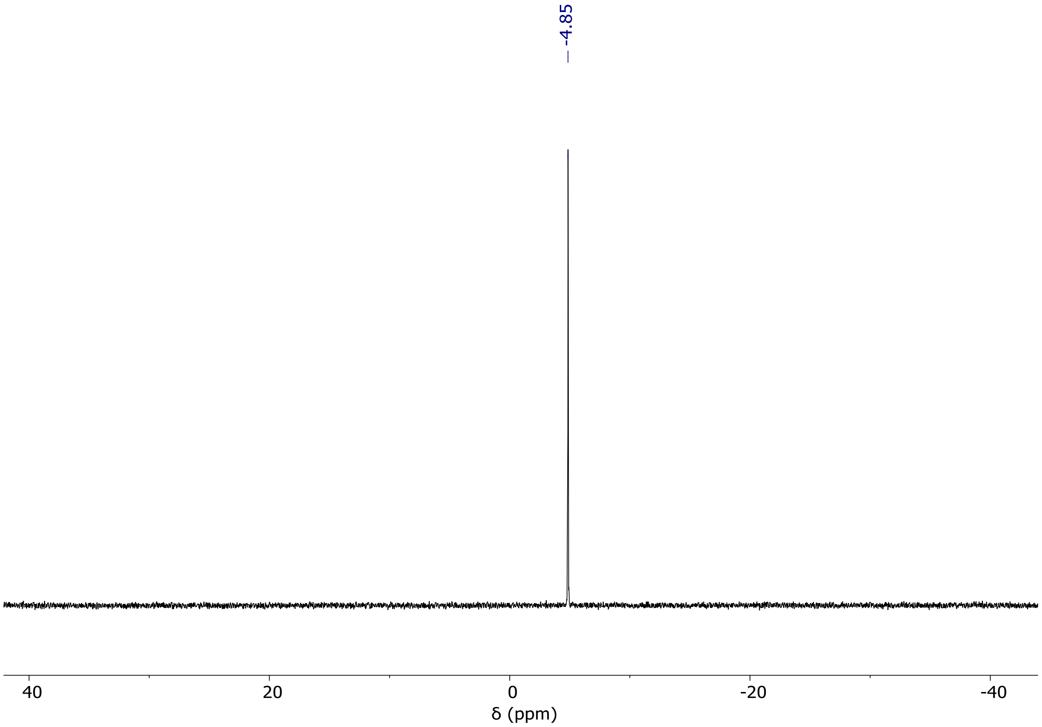

Figure S41. $^{31}P\{^1H\}$ NMR ($CDCl_3$) of **TATA PCF3**.

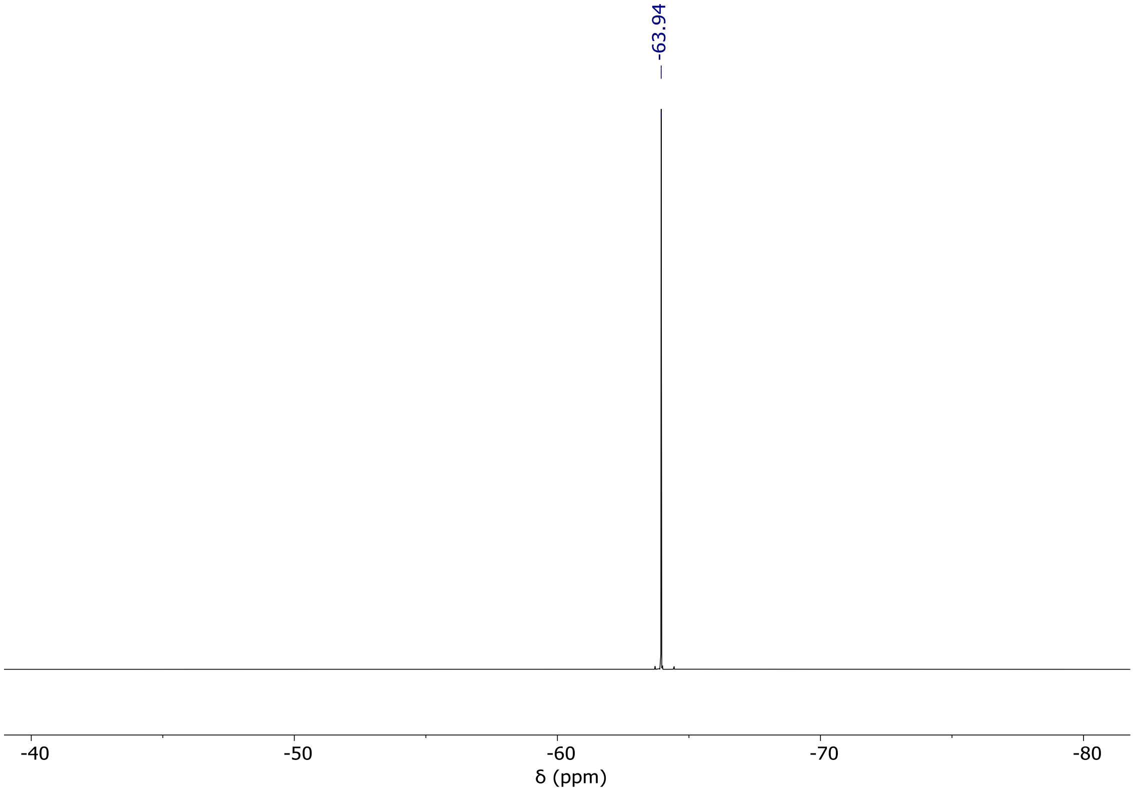

Figure S42. $^{19}F\{^1H\}$ NMR ($CDCl_3$) of **TATA PCF3**.

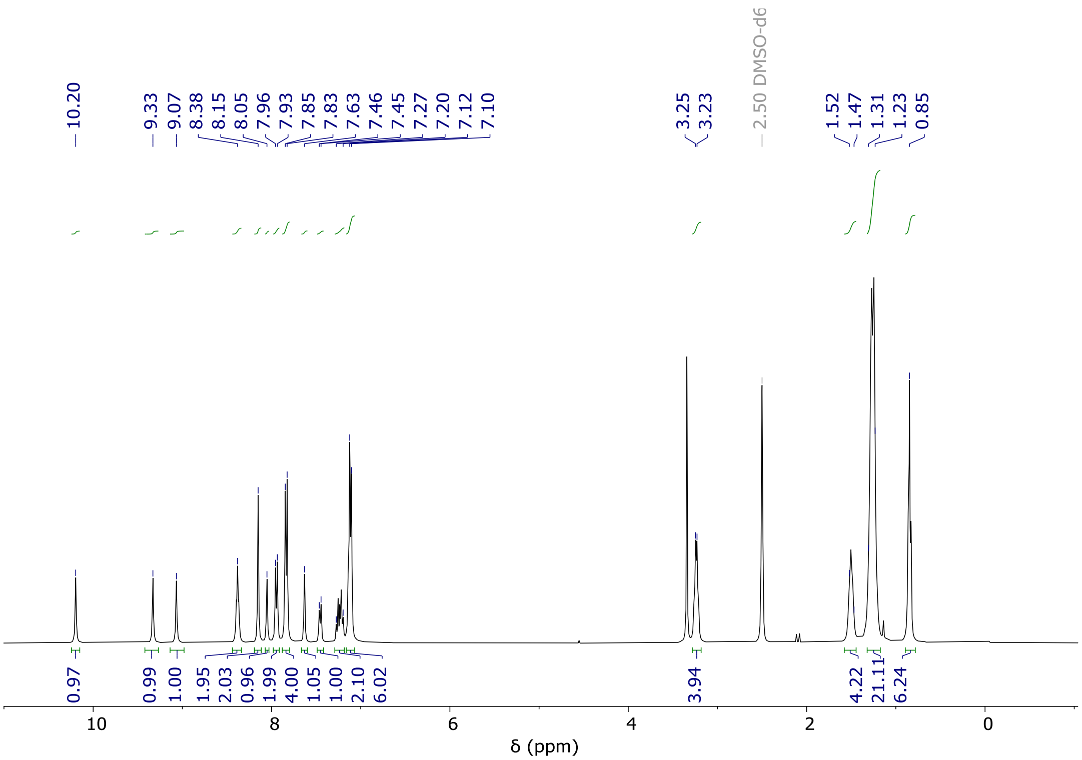

Figure S43. ^{1}H NMR (DMSO-d$_6$) of **TATA *m*-U**.

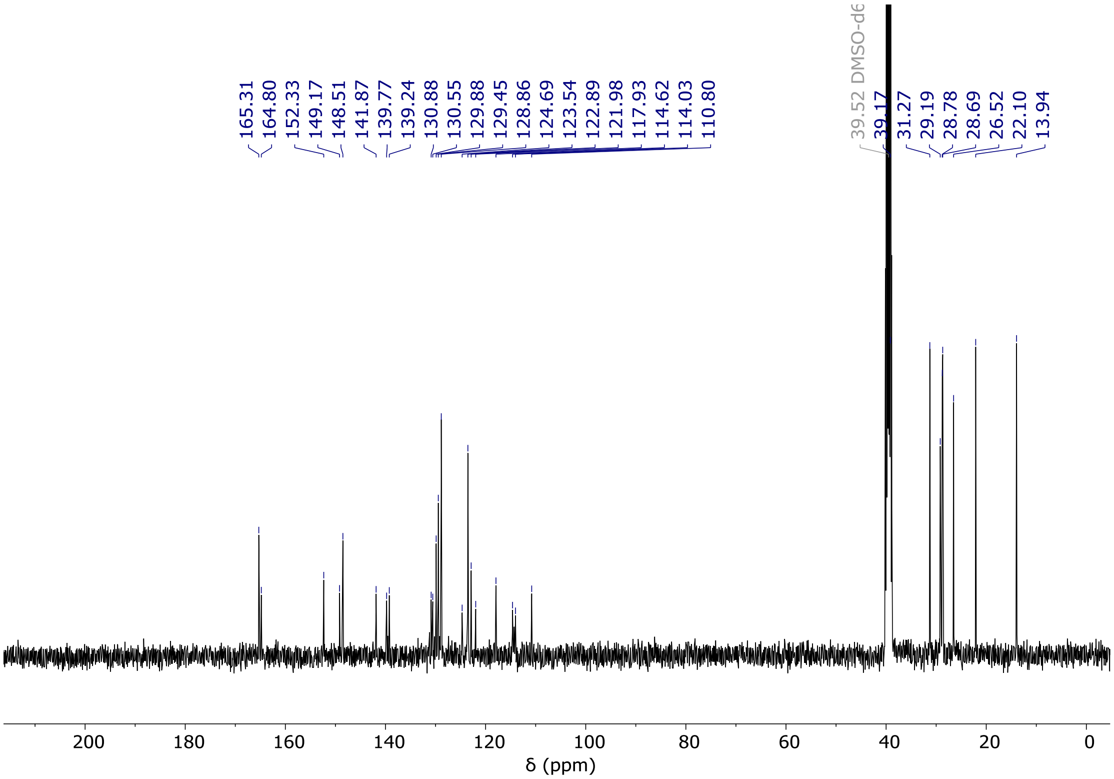

Figure S44. ^{13}C{^{1}H} NMR (DMSO-d$_6$) of **TATA *m*-U**.

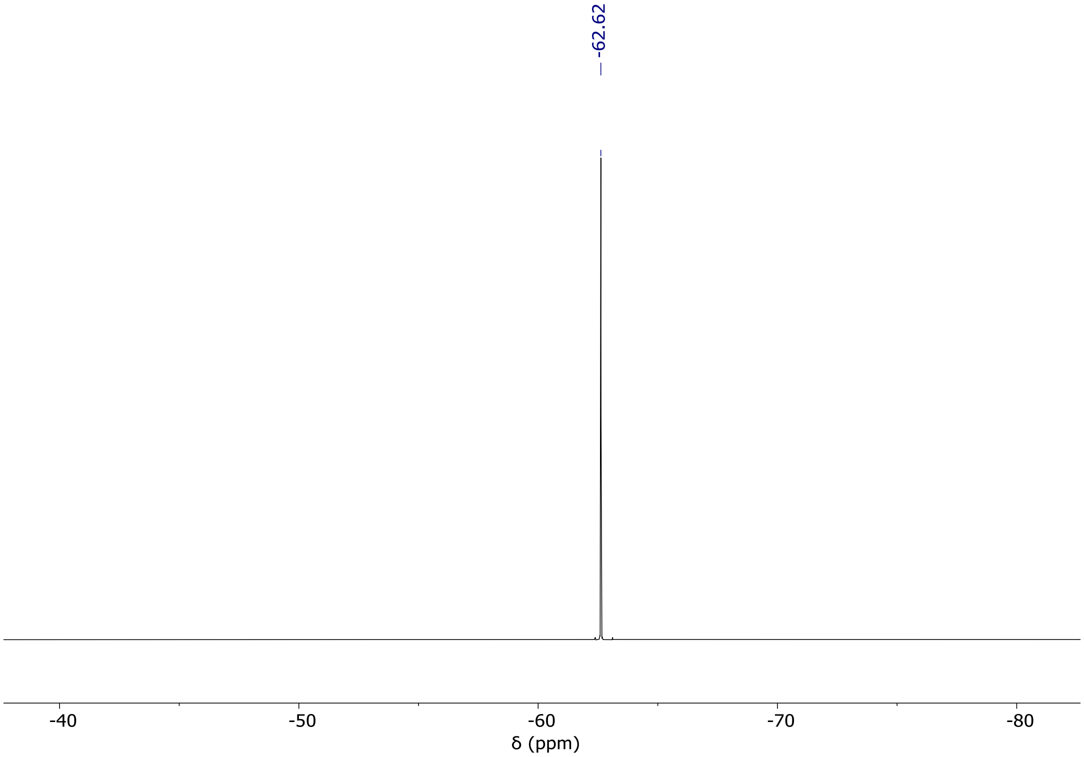

Figure S45. $^{19}F\{^{1}H\}$ NMR (DMSO-d_6) of **TATA *m*-U**.

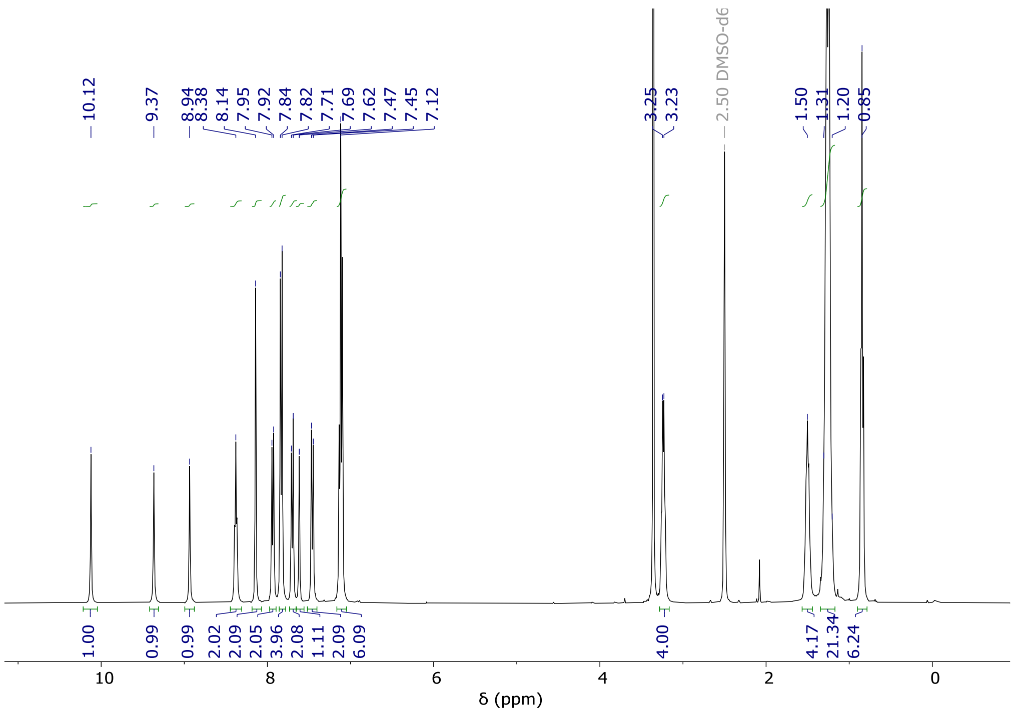

Figure S46. ^{1}H NMR (DMSO-d_6) of **TATA *p*-U**.

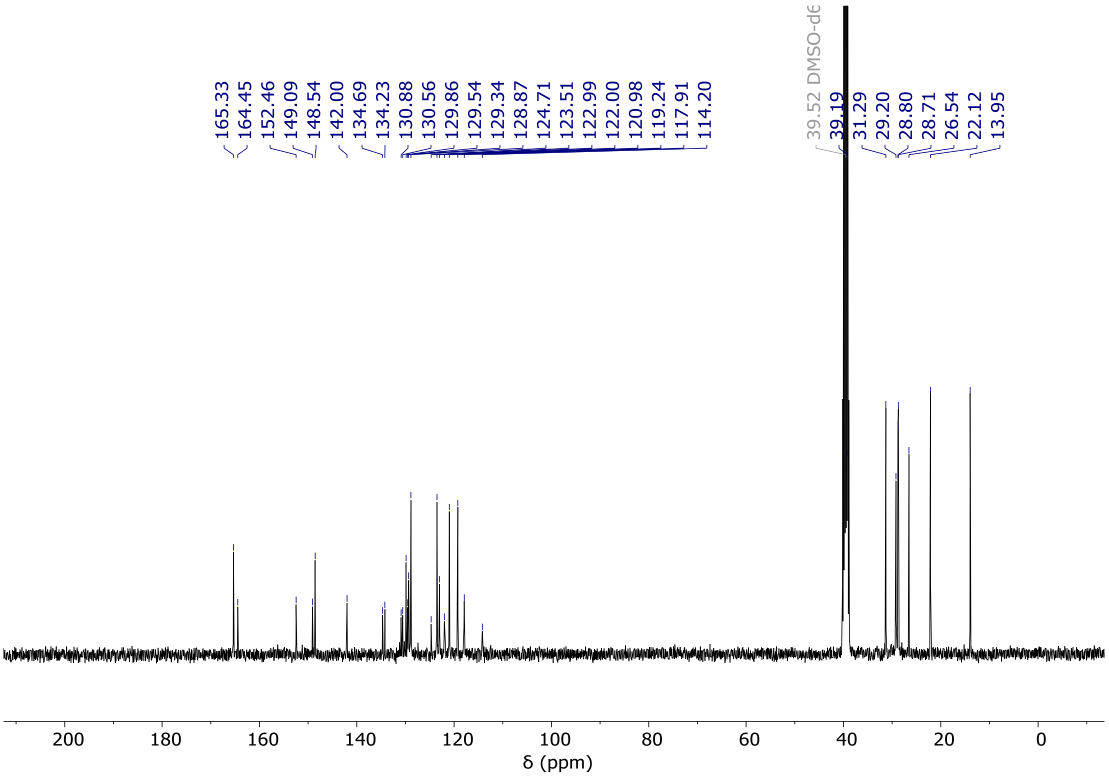

Figure S47. $^{13}C\{^{1}H\}$ NMR (DMSO-d_6) of **TATA *p*-U**.

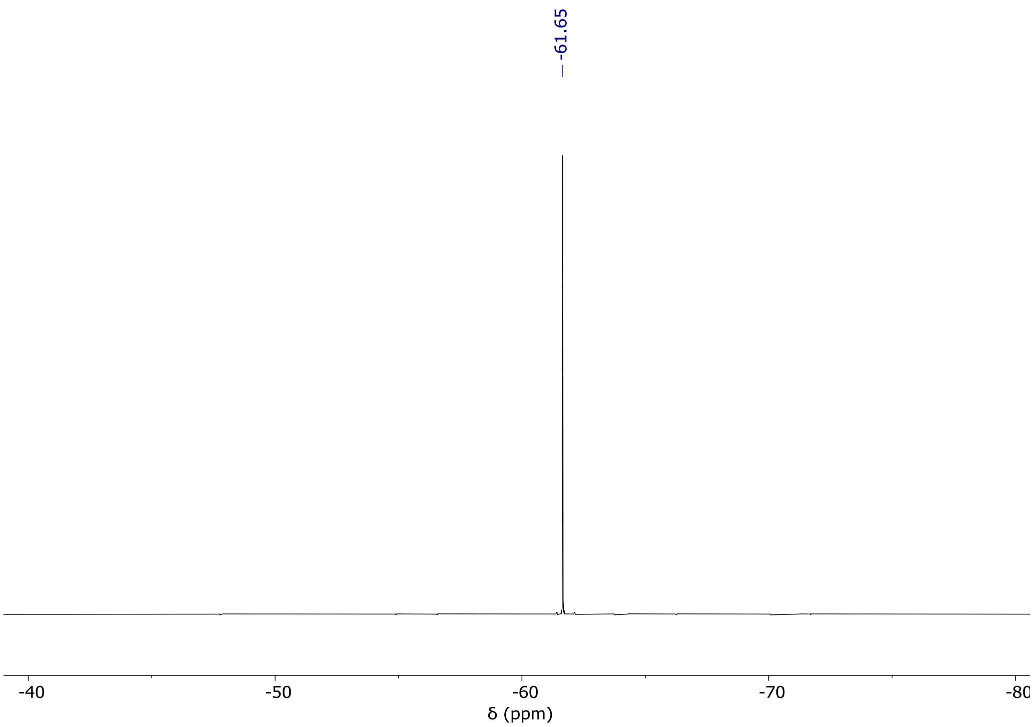

Figure S48. $^{19}F\{^{1}H\}$ NMR (DMSO-d_6) of **TATA *p*-U**.

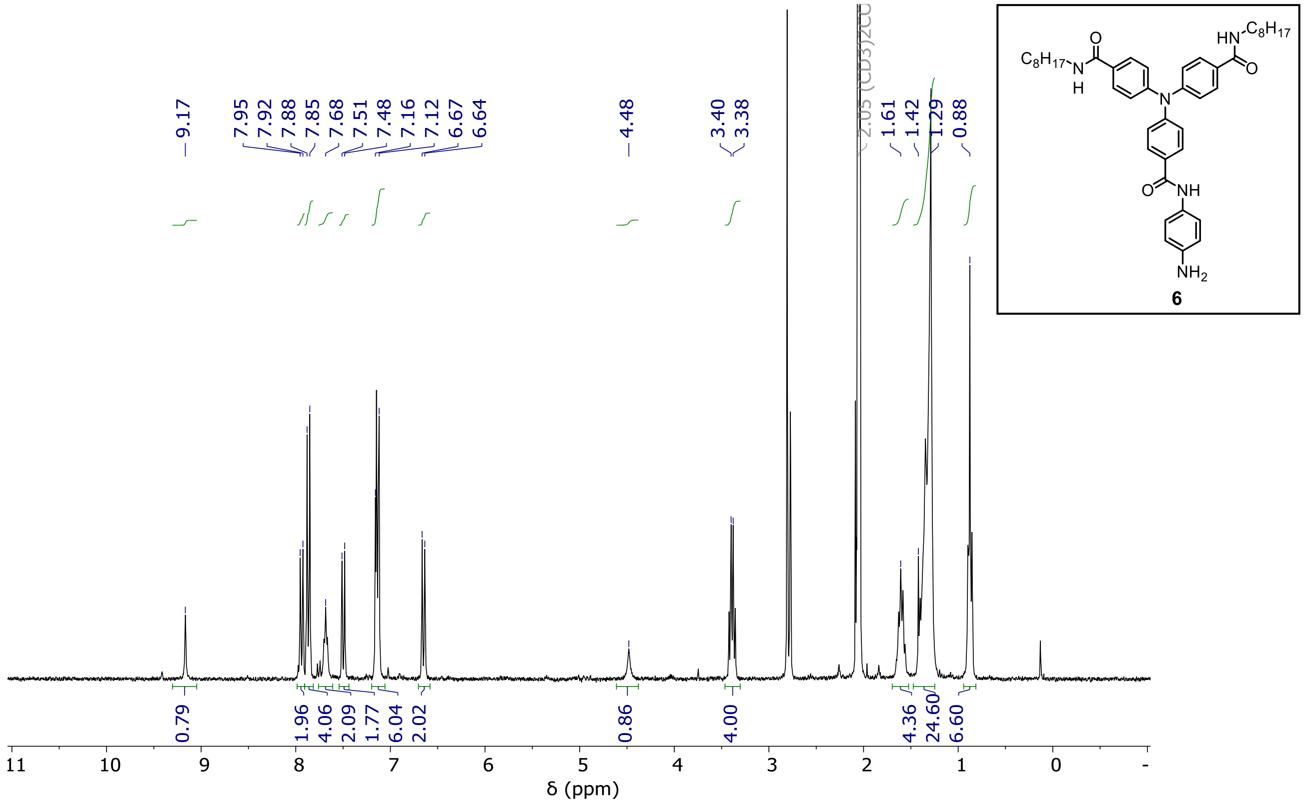

Figure S49. ^{1}H NMR (acetone-d_6) of compound **6**. The NH_2 group at δ= 4.48 ppm has been partially exchanged with D.

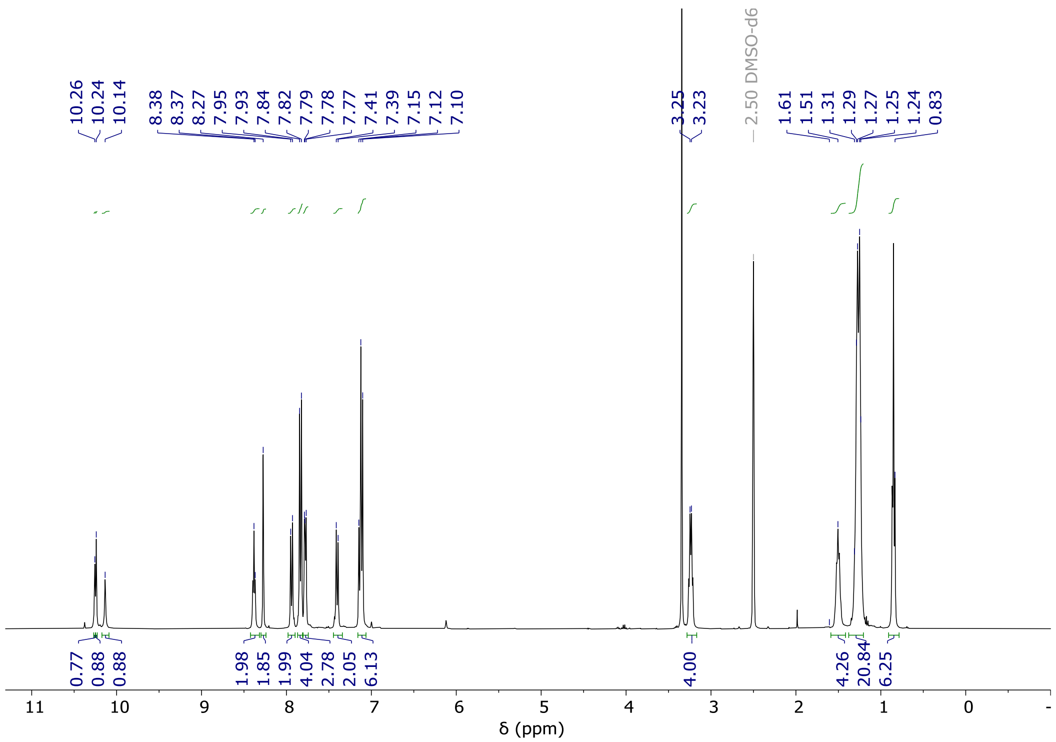

Figure S50. ^{1}H NMR (DMSO-d_6) of **TATA TU**.

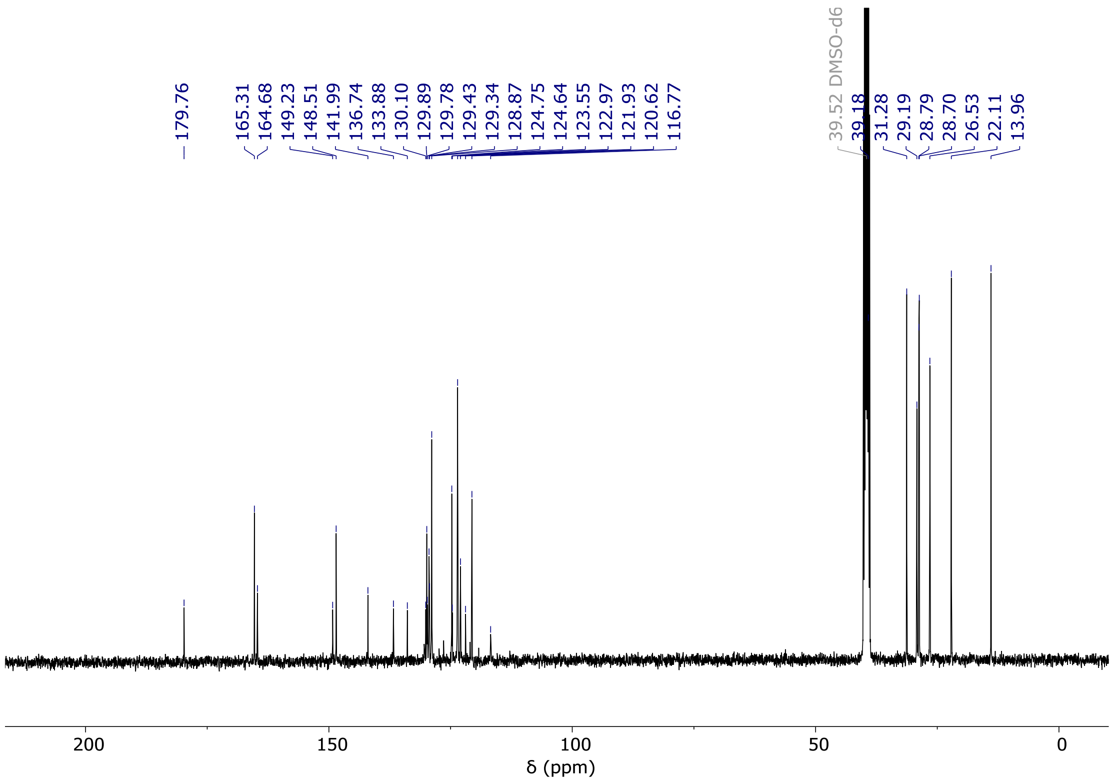

Figure S51. $^{13}C\{^{1}H\}$ NMR (DMSO-d_6) of **TATA TU**.

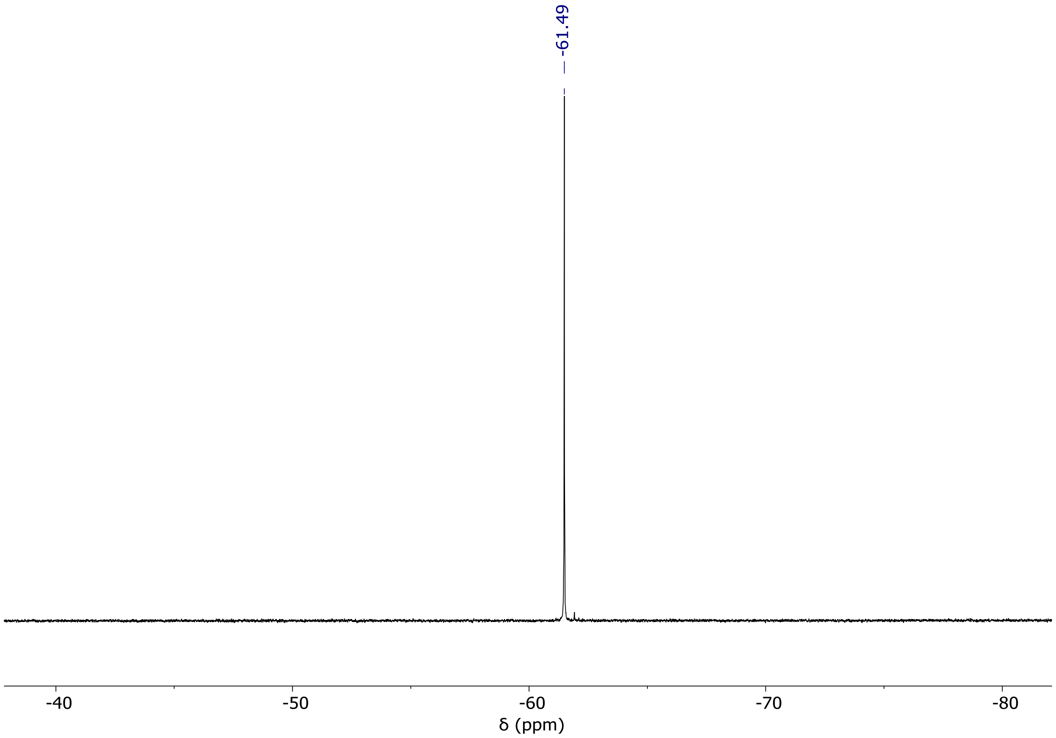

Figure S52. $^{19}F\{^{1}H\}$ NMR (DMSO-d_6) of **TATA TU**.

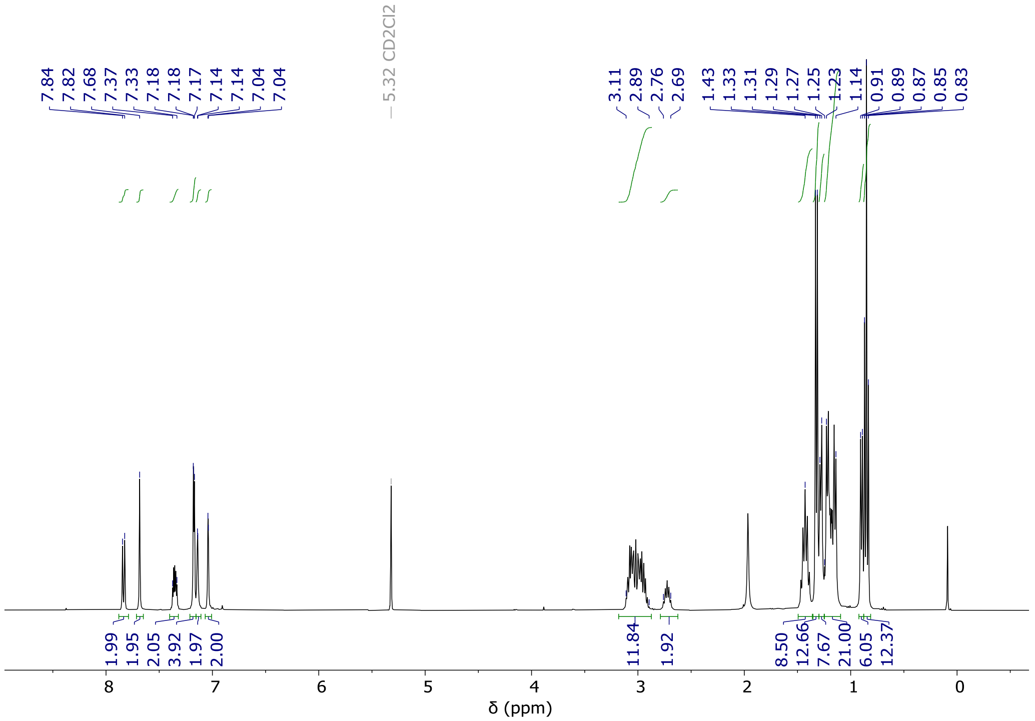

Figure S53. ^{1}H NMR (CD_2Cl_2) of **(*S*)-TRIP•NBu$_4$.** Signal at δ= 1.82 ppm is assumed to be residual water from CD_2Cl_2

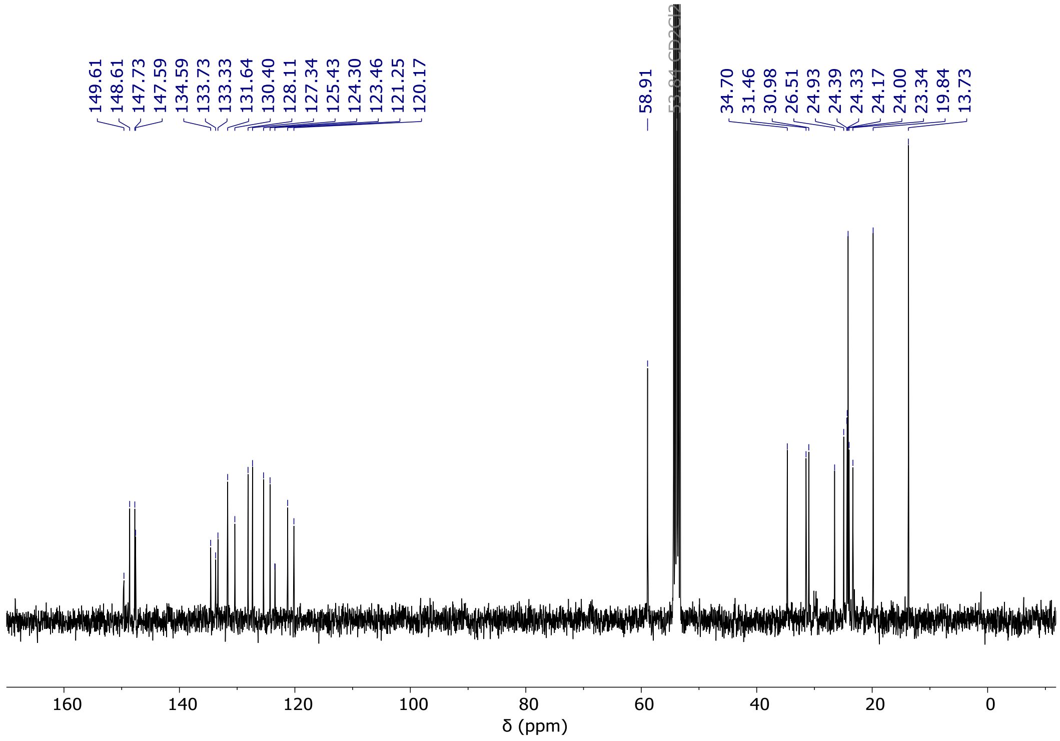

Figure S54. $^{13}C\{^1H\}$ NMR (CD_2Cl_2) of **(*S*)-TRIP•NBu$_4$.**

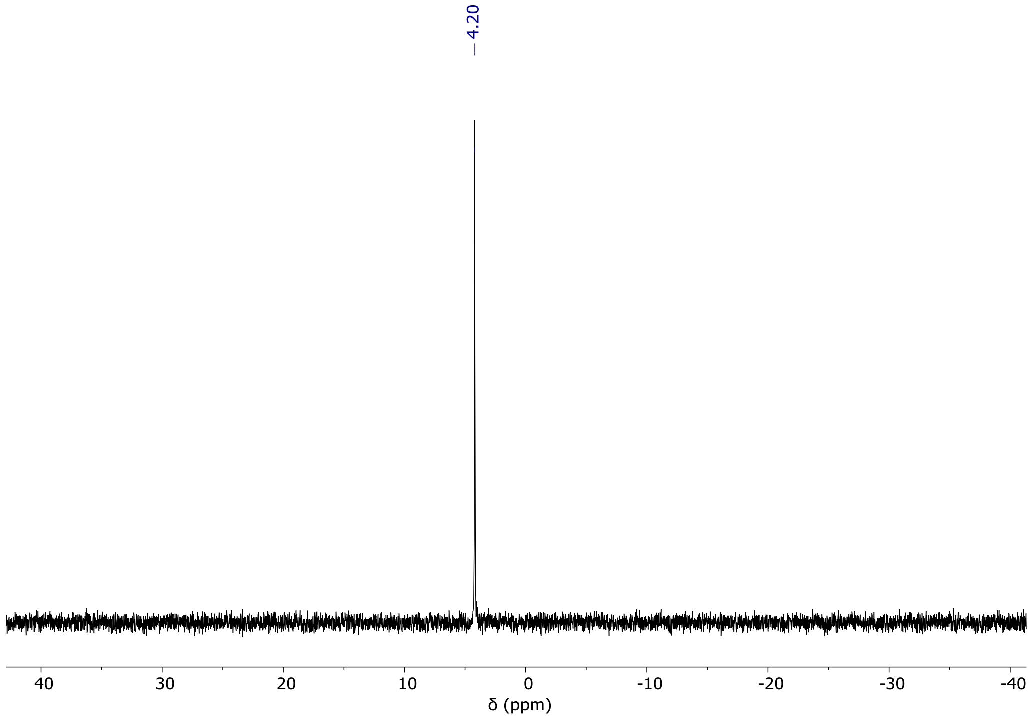

Figure S55. ^{13}P{^{1}H} NMR (CD_2Cl_2) of ***(S)*-TRIP•NBu₄**.

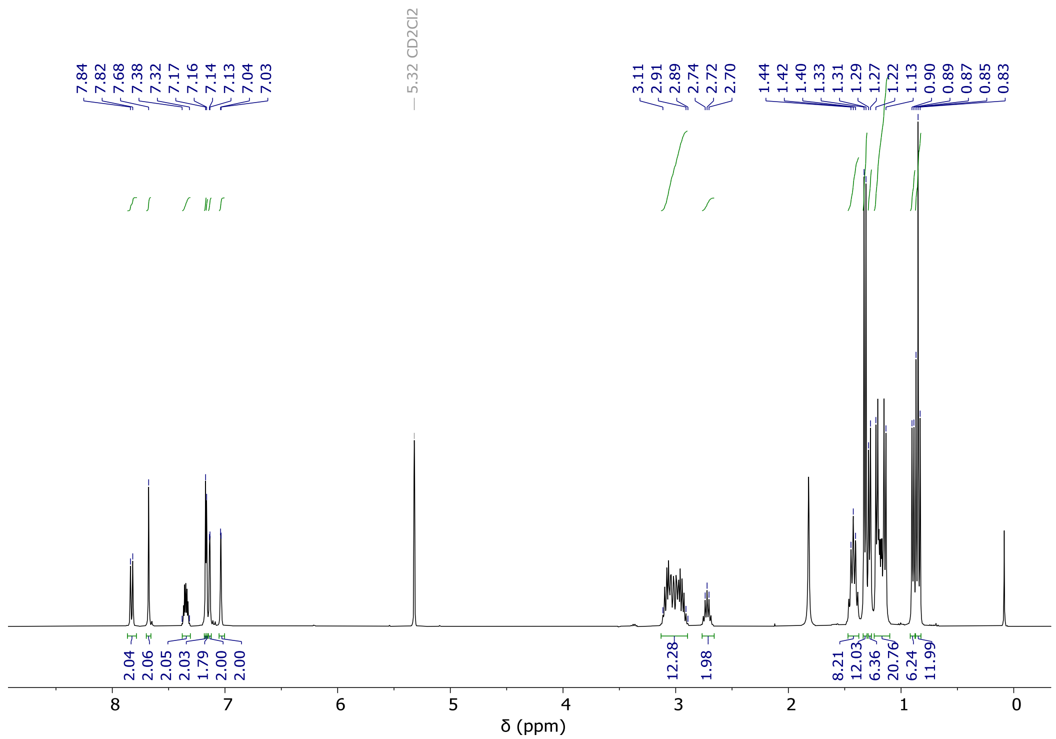

Figure S56. ^{1}H NMR (CD_2Cl_2) of **(*R*)-TRIP•NBu4**. Signal at δ= 1.82 ppm is assumed to be residual water from CD_2Cl_2

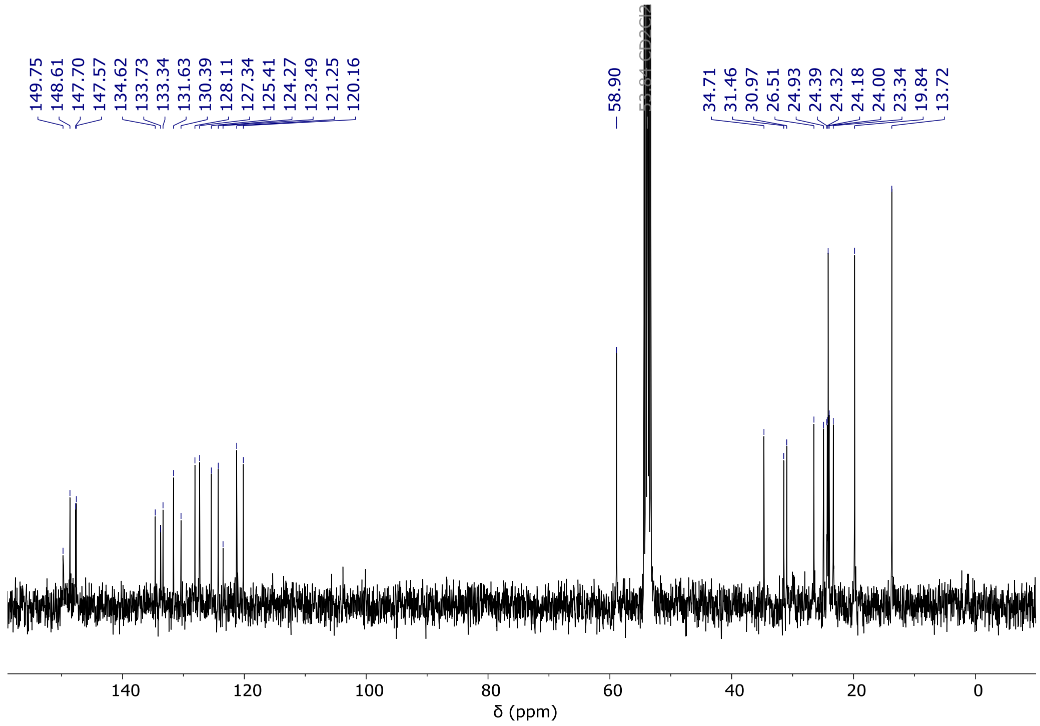

Figure S57. $^{13}C\{^{1}H\}$ NMR (CD_2Cl_2) of **(*R*)-TRIP•NBu4**.

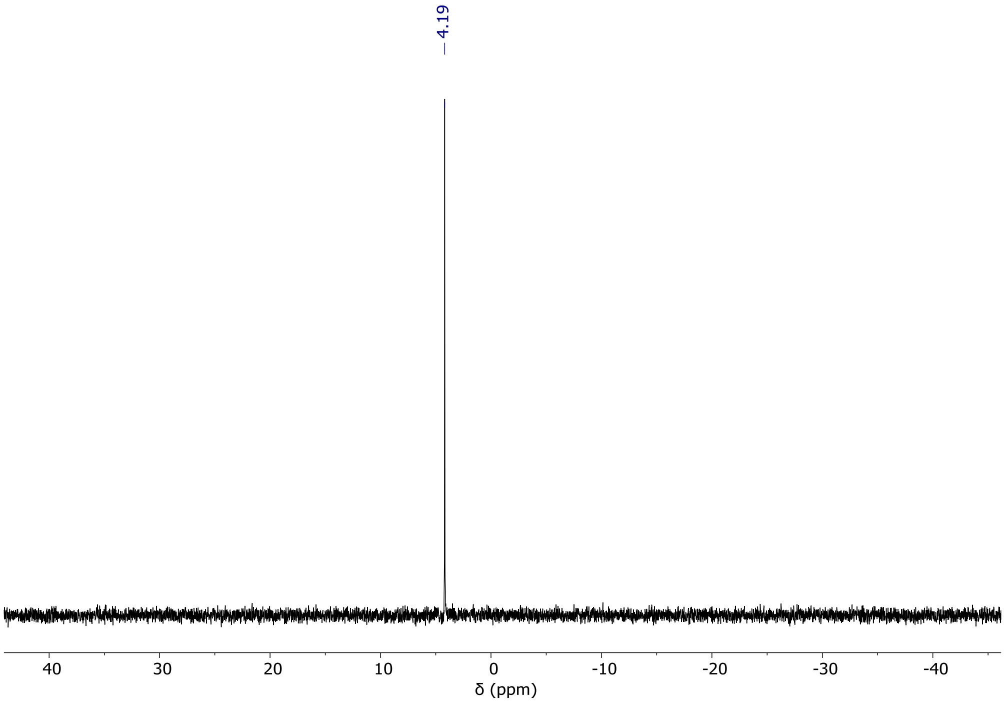

Figure S58. $^{13}P\{^{1}H\}$ NMR (CD_2Cl_2) of ***(R)*-TRIP•NBu$_4$**.

References

1. Q. Sallembien, P. Aoun, S. Blanchard, L. Bouteiller and M. Raynal, *Chem. – Eur. J.*, 2023, **29**, e202203199.
2. B. Adelizzi, I. A. W. Filot, A. R. A. Palmans and E. W. Meijer, *Chem. – Eur. J.*, 2017, **23**, 6103–6110.
3. M. A. Martínez-Aguirre, Y. Li, N. Vanthuyne, L. Bouteiller and M. Raynal, *Angew. Chem. Int. Ed.*, 2021, **60**, 4183–4191.
4. H. Kong, A. Valverde-González, R. Maruchenko, L. Bouteiller and M. Raynal, *Angew. Chem. Int. Ed.*, **64**, e202421991.
5. N. J. van der Heijden, P. Hapala, J. A. Rombouts, J. van der Lit, D. Smith, P. Mutombo, M. Švec, P. Jelinek and I. Swart, *ACS Nano*, 2016, **10**, 8517–8525.
6. R. Walker, H. Audorff, L. Kador and H.-W. Schmidt, *Adv. Funct. Mat.*, 2009, **19**, 2630–2638.
7. D. Gelman, L. Jiang and S. L. Buchwald, *Org. Lett.*, 2003, **5**, 2315–2318.
8. P. Aoun, A. Hammoud, M. A. Martínez-Aguirre, L. Bouteiller and M. Raynal, *Catal. Sci. Technol.*, 2022, **12**, 834–842.
9. Q. Zhang, H. Zhou, Arylurea compound, intermediate and use thereof, WO2012089137A1, 2012.
10. W. Tang, S. Johnston, J. A. Iggo, N. G. Berry, M. Phelan, L. Lian, J. Bacsa and J. Xiao, *Angew. Chem. Int. Ed.*, 2013, **52**, 1668–1672.
